# Synthesis, Structural Characterization, and In Vitro and In Silico Antifungal Evaluation of Azo-Azomethine Pyrazoles (PhN_2_(PhOH)CHN(C_3_N_2_(CH_3_)_3_)PhR, R = H or NO_2_)

**DOI:** 10.3390/molecules26247435

**Published:** 2021-12-08

**Authors:** Dorancelly Fernandez, Andrés Restrepo-Acevedo, Cristian Rocha-Roa, Ronan Le Lagadec, Rodrigo Abonia, Susana A. Zacchino, Jovanny A. Gómez Castaño, Fernando Cuenú-Cabezas

**Affiliations:** 1 Laboratorio de Química Inorgánica y Catálisis, Programa de Química, Universidad del Quindío, Carrera 15, Calle 12 Norte, Armenia 630004, Colombia; dfernadezm@uqvirtual.edu.co; 2 Instituto de Química UNAM, Circuito Exterior s/n, Ciudad Universitaria, Ciudad de México 04510, Mexico; acrestrepoa@uqvirtual.edu.co (A.R.-A.); ronan@unam.mx (R.L.L.); 3 Grupo GEPAMOL, Centro de Investigaciones Biomédicas, Universidad del Quindío, Carrera 15, Calle 12 Norte, Armenia 630004, Colombia; ccrochar@uqvirtual.edu.co; 4 Biophysics of Tropical Diseases, Max Planck Tandem Group, Universidad de Antioquia, Medellín 050010, Colombia; 5 Departamento de Química, Universidad del Valle, Calle 13 No. 100-00, A.A. 25360, Cali 76001, Colombia; rodrigo.abonia@correounivalle.edu.co; 6 Área Farmacognosia, Facultad de Ciencias Bioquímicas y Farmacéuticas, Universidad Nacional de Rosario (UNR), Suipacha 531, Rosario S2002LRK, Argentina; szaabgil@gmail.co; 7 Grupo Química-Física Molecular y Modelamiento Computacional (QUIMOL®), Facultad de Ciencias, Universidad Pedagógica y Tecnológica de Colombia (UPTC), Avenida Central del Norte, Tunja, Boyacá 050030, Colombia

**Keywords:** Schiff bases, azomethine compounds, *Candida albicans*, *Cryptococcus neoformans*, in silico studies, molecular docking, QTAIM-C, DFT

## Abstract

The azo-azomethine imines, R^1^-N=N-R^2^-CH=N-R^3^, are a class of active pharmacological ligands that have been prominent antifungal, antibacterial, and antitumor agents. In this study, four new azo-azomethines, R^1^ = Ph, R^2^ = phenol, and R^3^ = pyrazol-Ph-R’ (R = H or NO_2_), have been synthesized, structurally characterized using X-ray, IR, NMR and UV–Vis techniques, and their antifungal activity evaluated against certified strains of *Candida albicans* and *Cryptococcus neoformans*. The antifungal tests revealed a high to moderate inhibitory activity towards both strains, which is regulated as a function of both the presence and the location of the nitro group in the aromatic ring of the series. These biological assays were further complemented with molecular docking studies against three different molecular targets from each fungus strain. Molecular dynamics simulations and binding free energy calculations were performed on the two best molecular docking results for each fungus strain. Better affinity for active sites for nitro compounds at the “*meta*” and “*para*” positions was found, making them promising building blocks for the development of new Schiff bases with high antifungal activity.

## 1. Introduction

Azomethine compounds (Schiff bases), with the general formula RHC=NR’, are highly relevant ligands in pharmaceutical and medical chemistry. These structures are recognized for their high anticancer, antifungal, antiparasitic, antitubercular, cytotoxic, and herbicidal characteristics [1,2,3,4]. The substitution of the R and R′ groups with functionalized aromatic or heterocyclic moieties has been shown to be a promising pharmacological strategy to enhance the biological activity of azomethine ligands as anti-inflammatory [5], antitumor [6,7], antimicrobial [8], antidiabetic [9], antileishmaniacic [10], and antitubercular [11] agents, among others [12,13].

**Figure 1:**
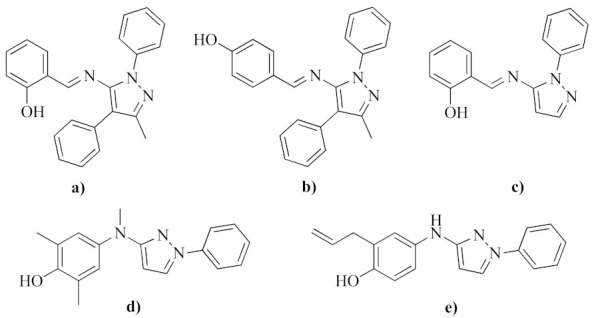
Examples of azomethine derivatives with functionalized pyrazole rings with broad and potent biological activity.

R′ substituted azomethine derivatives with functionalized pyrazole rings have revealed both potent and varied biological activities. Compounds such as 2- and 4-[(5-Methyl-2,4-diphenyl-2H-pyrazol-3-ylimino)-methyl]-phenol (structures (a) and (b) in Figure 1), have been evaluated as ligands in more than 700 biological assays each [14,15]. These compounds were revealed to have antileishmaniasis and antibacterial characteristics, inhibitors of CHOP to regulate the unfolded protein response to ER stress, binders of estrogen receptors, antagonist of Human D1 Dopamine Receptor: qHTS, molecule activators of BRCA1 expression, and inhibitors of KCNQ1 potassium channels, among others. Likewise, the compound 2-[(*E*)-(2-Phenylpyrazol-3-yl)iminomethyl]phenol (structure (c) in Figure 1), has been evaluated in multiple bioassays, thus identifying it as a potent inhibitor of the TEAD-YAP interaction [16]. Meanwhile, the reduced R-NR″-C_3_H_2_N_2_-Ph azomethine analogs (R = (HOPh)(Me)_2_- or CH_2_CHCH_2_-(PhOH)-, and R″ = H or Me, structures (d) and (e) in Figure 1) are well known to be effective anti-inflammatory agents [17,18].

**Figure 2:**
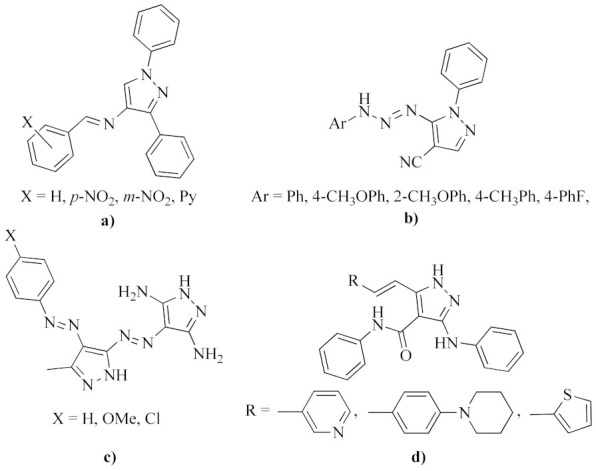
Representative azomethine derivatives with functionalized pyrazole rings with potent antifungal activity.

One of the most promising biological applications of azomethine pyrazole derivatives, as well as their azo analogs, has been their fungicidal activity. In this regard, Sreenivasa et al. recently carried out the synthesis of new Schiff bases by coupling a pyrazole nucleus through azomethine linkage with different aromatic amines [19]. The synthesized compounds were tested in vitro against *C. albicans* strains, showing significant antifungal activity compared to the standard drug fluconazole, especially for derivatives prepared from aromatic amines substituted with nitro groups in the *para* and *meta* positions (structure (a) in Figure 2). On the other hand, Al-Azmi recently synthesized a series of novel azopyrazole carbonitrile derivatives (structure (b) in Figure 2) which showed antifungal activities against *C. albicans* strains when the reference drug cycloheximide failed to do so with a concentration of 1 mg mL^−1^ [20]. Likewise, high antifungal effects have been reported against strains of *C. albicans* and *C. glabrata* for heterocyclic disazo dyes based on dipirazole rings, with electron-acceptor and electron-donor groups in their *ortho*, *meta* and *para* positions, (structure (c) in Figure 2) [21]. These dyes had a fungicidal effect like fluconazole against *C. albicans* strains and showed a lower active concentration than fluconazole against *C. glabrata* strains. Additionally, some Schiff bases of pyrazole-pyridine, pyrazole-thiophene and pyrazole-phenyl (structure (d) in Figure 2) have revealed effective antifungal activities against strains of *Aspergillus* mold species [22].

Due to the effective antifungal activity detected both for azomethine compounds containing pyrazole rings substituted with nitrated aromatics and for their azo derivatives [19,20,21,22], we have decided to design new Schiff bases constructed by assembling azo fragments functionalized with aromatic groups and aromatic pyrazoles substituted in *ortho*, *meta* or *para* positions by nitro groups. Therefore, this study has resulted in the synthesis, structural characterization (XRD, GC-MS, NMR, IR, and UV–Vis), and antifungal evaluation of four novel azo-pyrazole-azomethines with the general formula R-(PhOH)-(H)C=N-((C_3_HN_2_)(tert-butyl)(R’)) with R = Ph-N=N- and R′ = Ph- or -Ph-NO_2_ in the search for new azomethine Schiff bases derivatives [23,24]. Biological tests were performed against certified strains of *Candida albicans* (ATCC 10231) and *Cryptococcus neoformans* (ATCC 32264). Additionally, the antifungal assays were complemented with in silico studies using different target macromolecules.

## 2. Results and Discussion

### 2.1. Synthesis and Molecular Identification

**Figure 3:**
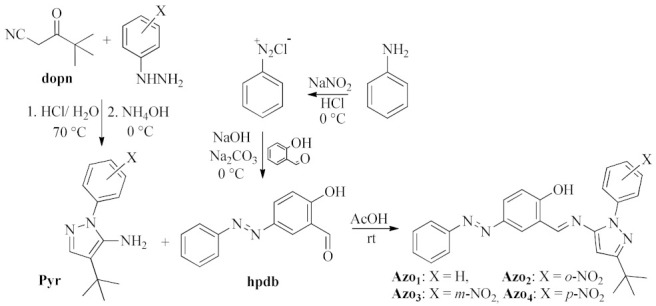
General synthetic scheme for the preparation of novel azoimine-pyrazole derivatives.

The new family of azo-pyrazole-azomethines derivatives (Schiff bases), labeled as Azo1 to Azo4 (Figure 3), were synthesized and resulted in a good yield (75–89%) using a simple and environmentally friendly technique that consisted of the direct condensation of pyrazole-amines (Pyr1 to Pyr4) and (*E*)-2-hydroxy-4-(phenyldiazenyl)benzaldehyde (hpdb) and mechanical maceration. Their molecular formula (C_26_H_25_N_5_O for Azo1 and C_26_H_24_N_6_O_3_ for Azo2 to Azo4) were initially identified by elemental analysis (Section 3.1.3) and further confirmed by GC–MS spectrometry. The mass fragmentation spectra and fragmentation patterns for compounds Azo1 to Azo4 are presented in the Supplementary Materials (see Figures S1–S5).

### 2.2. Spectroscopy Characterization

#### 2.2.1. Fourier Transform Infrared (FTIR) Spectroscopy

The experimental and theoretical IR spectra of compounds Azo1 to Azo4 are presented in Figure S6 in the Supplementary Materials, while the corresponding vibrational frequencies are listed and assigned in Table 1. High correlation coefficients (R^2^) of 0.990 (Azo1)**,** 0.998 (Azo2), 0.997 (Azo3), and 0.998 (Azo4) were obtained from the linear correlation curves between the calculated and experimental frequencies (see Figure S7 in Supplementary Materials). As can be observed in Table 1 and Figure S6, the O-H stretching mode was observable only in the IR spectrum of the Azo1 compound, as a low intensity band centered at 3440 cm^−1^. According to our spectroscopic results (FT-IR, NMR and UV–Vis) the Azo1–Azo4 molecules were found in their enol-imine form. Consequently, a proton transfer phenomenon was ruled out as the possible source of the absence of OH signals in the Azo2 to Azo4 series, since neither the N-H stretching band (expected around 3000 cm^−1^) nor the C=O stretching band (expected around 1800 cm^−1^) were observed in their FTIR spectra. Instead, the lack of this signal in the FTIR spectra of the Azo2 to Azo4 compounds was consistent with the formation of a stronger hydrogen bond (HB) between the hydrogen of the hydroxyl group and the nitrogen of the azomethine group [1,25], which is favored by the attractive electronic effects generated by the presence of the NO_2_ group on the aromatic ring of these three structures. Theoretical values for O-H vibration of the Azo1 to Azo4 series were calculated at 3152, 3159, 3157, and 3169 cm^−1^, respectively. The trend in the O-H···N=C HB strength in the series was also supported by the frequency order of the C=N stretching mode [1,26], which was observed as a strong signal at 1598 (Azo1), 1604 (Azo2), 1604 (Azo3), and 1605 (Azo4) cm^−1^, with estimated theoretical values at 1583, 1583, 1580, and 1580 cm^−1^, respectively.

#### 2.2.2. Nuclear Magnetic Resonance (NMR) Spectroscopy

**Figure 4:**
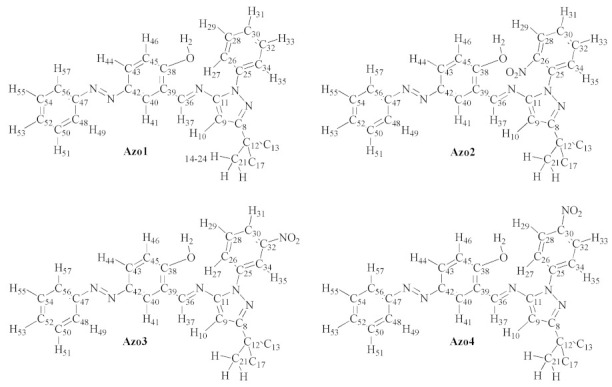
Atomic labeling used for NMR chemical shift assignment in the Azo1 to Azo4 series.

The assignment of chemical shifts (in ppm) in the ^1^H-NMR spectra of the Azo1–Azo4 series is presented in Table 2, which was carried out considering the nuclei labeling as shown in Figure 4.

The ^1^H-NMR spectra of the Azo1 and Azo4 compounds (Figures S8–S11 in Supplementary Materials) are characterized by the presence of 13 and 12 signals, which correspond to a total of 25 and 24 protons, respectively, as expected. However, the ^1^H-NMR spectra of the compounds Azo2 and Azo3 each show 13 signals that integrate for 23 protons, i.e., one proton less than expected. As noted in Table 2, the missing signal in the ^1^H-NMR spectra of the Azo2 and Azo3 compounds corresponds to the hydrogen (H-2) of the hydroxyl group, the absence of which suggests the formation of strong intermolecular interactions, e.g., O-H···O=S<, between the solvent molecules (dmso-*d6*) and this group. Figure S23 (in Supplementary Materials) shows the correlation coefficients (R^2^) plots obtained between the experimental and theoretical ^1^H chemical shifts, in all cases with values very close to 1.0.

The ^13^C-NMR spectrum of the Azo1 compound (Figure S8 in Supplementary Materials) showed 20 signals, while 22 signals were observed in each of the ^13^C-NMR spectra of the Azo2 to Azo4 compounds (Figures S9–S11 in Supplementary Materials). In all cases, the signals correspond to a total of 26 carbons, as expected. The assignments of these signals are presented in Table 3, which were made based on the analysis of the two-dimensional (DEPT, HSQC, HMBC, and COSY) NMR spectra (Figures S12–S22 in Supplementary Materials) and supported by the numerical values predicted by the DFT calculations. The correlation coefficient (R^2^) plots between the experimental and theoretical ^13^C chemical shifts are presented in Figure S24 (see Supplementary Materials), with values ranging between 0.96–0.99.

#### 2.2.3. Absorption Electronic UV–Visible Spectroscopy

**Figure 5:**
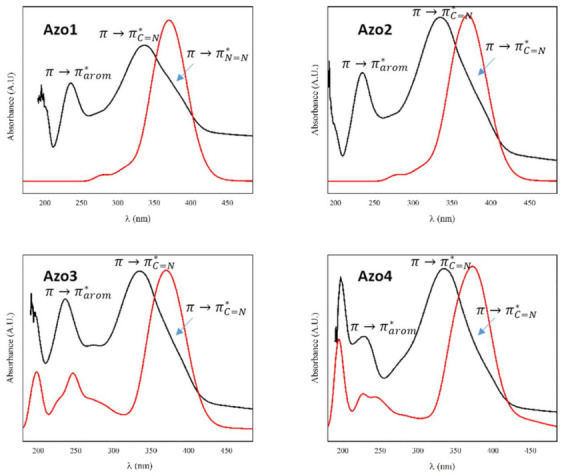
Experimental (in black) and theoretical (in red) UV–Vis spectra for compounds Azo1 to Azo4.

The experimental and calculated UV–Vis spectra of compounds Azo1 to Azo4 are presented in Figure 5, whereas the respective log ε value, excitation energy, and orbital contributions for each absorption are shown in Table S1 in Supplementary Materials. All compounds presented an experimental absorption band around 234 nm that is consistent with a typical π→π* transition in aromatic compounds [24,25,26,28]. Theoretical calculations indicate that this band is associated with the transition HOMO→LUMO+4 (77%, logε 4.461), HOMO→LUMO+3 (26%, logε 4.623), HOMO-3→LUMO+3 (79%, logε 4.715), and HOMO-1→LUMO+6 (78%, logε 5.051) for compounds Azo1 to Azo4, respectively, which is further associated with the pyrazole and the phenyl rings [1]. On the other hand, the absorption band observed at 335 nm in the UV–Vis spectra of this series was attributed to the HOMO-2→LUMO (75%, logε 4.575), HOMO→LUMO+2 (87%, logε 4.798), HOMO→LUMO+2 (86%, logε 4.832), and HOMO→LUMO+2 (86%, logε 5.369) transitions, respectively, and further related to the azomethine group -C=N [23,25]. Finally, the shoulder at around 362 nm in these spectra was assigned to the π→π* transition related to the azo group (N=N), linked to the aromatic groups.

### 2.3. Frontier Molecular Orbitals

**Figure 6:**
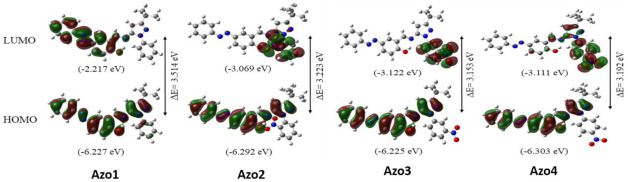
Molecular surfaces and energies for the HOMO and LUMO frontier orbitals in the gas phase molecules Azo1 to Azo4, calculated using the B3LYP/6-311++g(d,p) approximation level.

Figure 6 shows the molecular surfaces, energies, and energy gaps (∆E) for the HOMO and LUMO orbitals in the isolated molecules Azo1 and Azo2 calculated using the B3LYP/6-311++G(d,p) level of approximation. The spatial distribution of the HOMO orbitals in all compounds (Figure 6) is related to an extensive *pi* delocalization coplanar molecular system that involves the phenyl ring of the azo group, the phenol ring, the imine group, and the pyrazole ring. These fragments correspond to electron donor sites in this series of molecules. On the other hand, the LUMO orbital showed significant differences between the compound Azo1 and its nitro congeners (Azo2 to Azo4). In Azo1, the LUMO orbital is related to a *pi* delocalization system that encompasses the phenyl azo ring, the phenol ring, and the imine group, while in the Azo2 to Azo4 compounds the LUMO orbital is centered on the nitro-substituted aromatic ring. HOMO-LUMO orbital gaps (∆E) also show differences between the Azo1 compound and the Azo2 to Azo4 set. Nitro derivatives (Azo2–Azo4) are characterized by a lower ∆E value (3.153–3.223 eV) compared to Azo1 ∆E (3.514 eV). A lower ∆E gap together with the opposite spatial distribution of the HOMO and LUMO orbitals enables an intramolecular charge transfer that favors the action of nucleophilic attacks on the Azo2–Azo4 series. The extension of the pi delocalization system in the Azo1 to Azo4 series is schematized using resonance structures in Figure S25 (in Supplementary Materials).

### 2.4. Crystalline Structural Analysis

#### 2.4.1. Single Crystal X-ray Diffraction

All compounds of the Azo1 to Azo4 series formed individual yellow (Azo1, Azo3, and Azo4) and brown (Azo2) crystals as the toluene solvent slowly evaporated. Details of the crystallographic data and refinement parameters of these solids are summarized in Table S2 (in Supplementary Materials).

**Figure 7:**
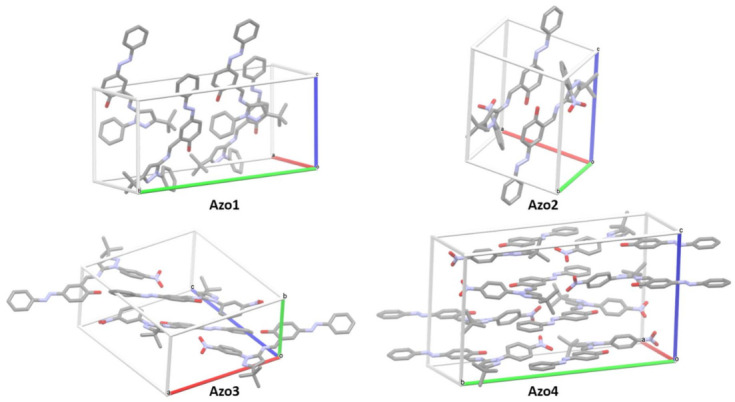
Molecular packing diagrams in the unit cell of the Azo1 to Azo4 crystals. Hydrogen atoms are not shown.

The compounds Azo1, Azo3, and Azo4 crystallized in monoclinic lattices with symmetries corresponding to the space groups *Cc*, *P*2_1_/c, and *P*2_1_/c respectively, while the compound Azo2 crystallized in a triclinic lattice according to the space group P1. The crystals from the Azo1 and Azo3 compounds each presented four molecules per unit cell, with cell lengths of *a* = 10.4561 (9), *b* = 21.462 (2), and *c* = 10.3280 (9) Å, β= 96.416(3)° and a cell volume of 2303.18 Å^3^ for the Azo1, meanwhile Azo3 crystal presented values of *a* = 17.0887(15), *b* = 7.3346(7), and *c* = 19.741(3) Å, β = 102.358(8)° and a cell volume of 2416.98 Å^3^. The Azo2 compound had two molecules per unit cell, with cell values of *a* = 9.5873(8), *b* = 11.2315(9), and *c* = 12.4374(10) Å, α = 82.489(2)°, β = 73.168(2)°, γ = 75.204(2)° and a cell volume of 1237.04 Å^3^. The Azo4 crystal was the only one in the series that showed two crystallographically distinct molecular units and a total of eight molecules per unit cell. The cell parameters of the Azo4 crystal were: *a* = 14,217(2), *b* = 23,397(4), and *c* = 15,917 (2) Å, β = 112,515(3)°, and a cell volume of 4891 Å^3^. Molecular representations of crystal packing in Azo1 to Azo4 compounds are presented in Figure 7.

In the Azo1 crystal, two of the four molecular units project semi-parallel into the cell along the *c* direction (Figure 7). These two molecules interact with each other through mutually intercalated H-O···H-C hydrogen bonds (HB) with a distance of 2.692 Å, formed between the hydroxyl hydrogen of one unit and an ortho hydrogen in the phenolic ring of the other unit. The other two molecules of the Azo1 crystal lie on opposite sides in the *cb* plane of the unit cell (see Figure 7). Each of these molecules forms infinite chains along the *c* direction that are held together through intermolecular N=N···H-C interactions (*d* = 2.679 Å), formed between an azo nitrogen (that is linked to the aromatic ring) of one unit and an ortho aromatic hydrogen of the next unit. This same interaction is also present throughout the infinite chains formed by each of the two molecules located within the unit cell. Additionally, the chains formed by the molecules of the *cb* plane interact with each other by means of H-O···H-C HBs (*d* = 2.692 Å), formed between an oxygen and the ortho hydrogen of their phenol rings.

The two molecules of the Azo2 crystal in the unit cell are packed in the *a* direction in an antiparallel fashion (see Figure 7). Both molecules interact with each other through two identical C-H···N=N HBs (*d* = 2.667 Å), formed between the ortho hydrogen of the derivative-nitro ring and the azo nitrogen closest to the aromatic ring. Each molecule forms infinite chains that propagate along the *c* direction by means of H-O···H-C HBs (*d* = 2.525 Å), formed between the hydroxyl hydrogen and a meta hydrogen of the aromatic ring and pi-stacking interactions between the aromatic rings (*d* = 2.854 Å). The interactions between pairs of molecules propagate along the *a* direction through mutual hydrogen bonds N=O···H-C = C (*d* = 2.413 Å), formed between an oxygen of the nitro group and the imido hydrogen. The second oxygen of the nitro group has distances of 3.011 and 3.177 Å with a methyl hydrogen and the hydrogen of the pyrazole ring of the next molecule located in *a* direction, respectively.

Crystalline packing in Azo3 is characterized by the stacking along the *b* direction in pairs of antiparallel molecules located in the center of the unit cell, while two other diametrically opposite molecules are located centered on the origin vertex (abc), see Figure 7. The pairs of antiparallel molecules are stabilized by mutual pi-stacking interactions between the aromatic ring and the nitro-derived aromatic ring. Each nitro group forms three different types of HBs: one nitro oxygen forms an HB (*d* = 2.705 Å) with the ortho hydrogen of the phenol of a second unit, while the second nitro oxygen forms an HB (*d* = 2.635 Å) with the meta hydrogen of phenol and an HB (*d* = 2.675 Å) with the imino hydrogen of a third unit. Meanwhile, the oxygen of the hydroxyl group forms an HB (*d* = 2.694 Å) with the meta hydrogen of the aromatic ring of another unit.

The eight molecules in the unit cell in the Azo4 crystal stack in the *c* direction to form four layers, each layer propagating infinitely in the *b* direction (see Figure 7). Inside each layer, the molecules interact with each other through N=O···H-C HBs (*d* = 2.569 and 2.650 Å), formed from the same nitro oxygen with the imido hydrogen and a meta hydrogen of the phenol group, respectively. The layers interact with each other through pi-stacking interactions formed between the nitro-aromatic ring and the phenol ring (2.895 Å) or the aromatic ring (2.900 Å).

#### 2.4.2. Topological Analysis on Electron Density (ρ)

**Figure 8:**
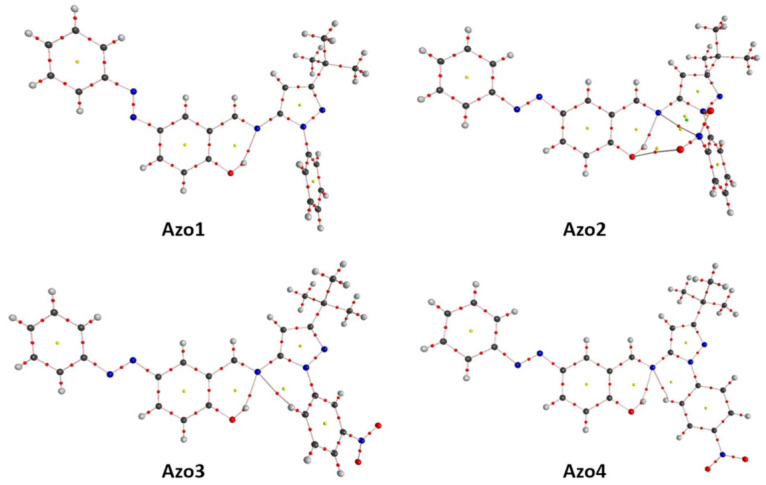
Molecular graphs for the Azo1 to Azo4 molecules as they appear in crystalline structures. Bond paths (pink lines), BCPs (red dots), RCPs (yellow dots), and CCPs (green dots).

The application of QTAIM [29] to the isolated molecules extracted from the Azo1 to Azo4 crystals allowed the detection of 123, 135, 129, and 129 critical points (CPs), respectively, which were classified according to their rank (ω) and signature (σ) as presented in Table 4. In all cases, the Poincaré–Hopf relationship (PHR) was satisfied (*n_NCP_* − *n_BCP_* + *n_RCP_* − *n_CCP_* = 1), thus verifying the completeness of these characteristic sets. The molecular graph for each of the molecules from Azo1 to Azo4 are presented in Figure 8, while the main topological properties of the BCPs found for these isolated molecular systems are presented in Tables S3–S6 (in Supplementary Materials).

The 61 BCPs (3,-1) detected in the Azo1 molecule topology comprised the 60 expected covalent BCPs and one closed-shell BCP. The former was related to a bond path (distance = 1.90 Å, ϱ=0.034 au, ε=0.029, and $\nabla^{2}=0.124$ au) between the hydroxyl hydrogen and the nitrogen of the imine moiety (Figure 8). As observed in Figure 8, the five retrieved RCP (3,+1) are associated to: the two aromatic rings, the phenol ring, the pyrazole ring, and the six-membered pseudo-ring formed as a result of the intramolecular O-H···N=C HB.

Apart from the expected 62 covalent BCPs (3,−1), the ρ topology of Azo2 molecule presents four closed-shell BCPs. The latter revealed the formation of a long O-O bond path (distance = 3.48 Å, $\varrho =2.96\times 10^{-3}$ au, ε=1.488, and $\nabla^{2}=3.13\times 10^{-3}$ au) between a nitro oxygen and the hydroxyl oxygen, an N-N bond path (distance = 2.94 Å, $\varrho =9.24\times 10^{-3}$ au, ε=1.183, and $\nabla^{2\;}=9.41\times 10^{-3}$ au) between nitro nitrogen and imine nitrogen, a O-N bond path (distance = 2.84 Å, ϱ=0.013 au, ε=7.511, and $\nabla^{2}=0.014$ au) between the second nitro oxygen and one of the pyrazole nitrogens, and a OH···N=C bond path (distance = 1.90 Å, ϱ=0.033 au, ε=0.034, and $\nabla^{2}=0.127$ au) similar to that observed in the Azo1 molecule. As a consequence of the formation of four RCPs (3,+1) related to the nitro group in *ortho* position, a CCP (3,+3) was detected for the Azo2 molecule (Figure 8).

For each of the crystalline molecules from the Azo3 and Azo4 compounds, 64 BCPs (3,-1) were found which comprise the expected 62 covalent BCPs and two closed-shell BCPs. The latter revealing the formation of two intramolecular bonding paths with the nitrogen of the imino group; one with the hydroxyl hydrogen O-H···N=C (distance = 1.85 Å, ρ=0.037 au, ε=0.033, and $\nabla^{2}=0.129$ au for Azo3, and distance = 1.91 Å, ρ=0.033 au, ε=0.029, and $\nabla^{2\;}=0.103$ au for Azo4) and the other with the hydrogen in the ortho position of the nitro-aromatic ring C-H···N=C (distance = 2.56 Å, ϱ=0.013 au, ε=0.325, and $\nabla^{2\;}=0.044$ au for Azo3 and 2.49 Å, ϱ=0.013 au, ε=0.240, and $\nabla^{2\;}=0.047$ au for Azo4) as seen in Figure 8. Consequently, the six RCPs (3,+1) retrieved for each of these molecules are related to: the aromatic, nitro-aromatic, phenol, and pyrazole rings, as well as the two pseudo-rings derived from the formation of the two intramolecular bond paths.

### 2.5. In Vitro and In Silico Studies on the Antifungal Activity

#### 2.5.1. Antifungal Activity Evaluation

**Figure 9:**
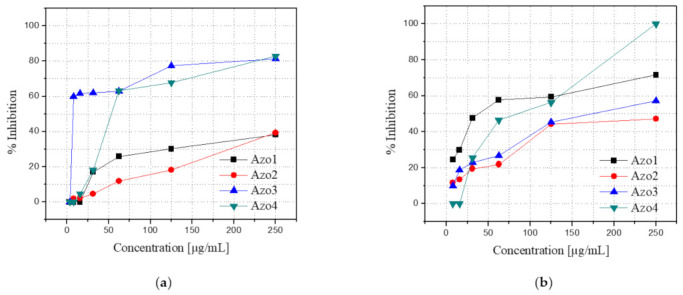
Percentages of inhibition of the compounds Azo1 to Azo2 as a function of the concentration against strains of fungi *C. albicans* (**a**) and *C. neoformans* (**b**).

Each Azo1–Azo4 compound was dissolved in RPMI-1640 at concentrations of 3.9, 7.8, 15.6, 31.2, 62.5, 125, and 250 μg/mL and its antifungal activity was evaluated against certified strains of *C. albicans* (ATCC 10231) and *C. neoformans* (ATCC 32264) using the standardized microplate method of the Clinical and Laboratory Standard Institute (CLSI) [30]. In these assays, amphotericin B was used as a positive control, for which 100% inhibition was achieved for both strains in the entire range of concentrations used. The results of the in vitro antifungal evaluation for the Azo1–Azo4 series are graphically presented in Figure 9.

As observed in Figure 9a, the Azo3 compound exhibited the highest inhibitory activity against the *C. albicans* strain, for which an inhibition of 59.8% was recorded using a concentration of 7.8 μg/mL. On the other hand, the compound Azo1, Figure 9b, exhibited the best activity against the *C. neoformans* strain. For this compound, an inhibition of 47.6% was recorded using a concentration of 31.2 μg/mL. In contrast, the compound Azo2 was the agent in the series that revealed the lowest antifungal activity against both strains. The compound Azo4 was the only member of the series to exhibit 100% inhibition against the *C. neoformans* strain, using a concentration of 250 μg/mL. At this same concentration, the Azo4 compound exhibited the highest inhibition (82.9%) of the entire series against the *C. albicans* strain, closely followed by an 81.4% inhibition exhibited by the Azo3 compound.

#### 2.5.2. Molecular Docking Modelling

**Figure 10:**
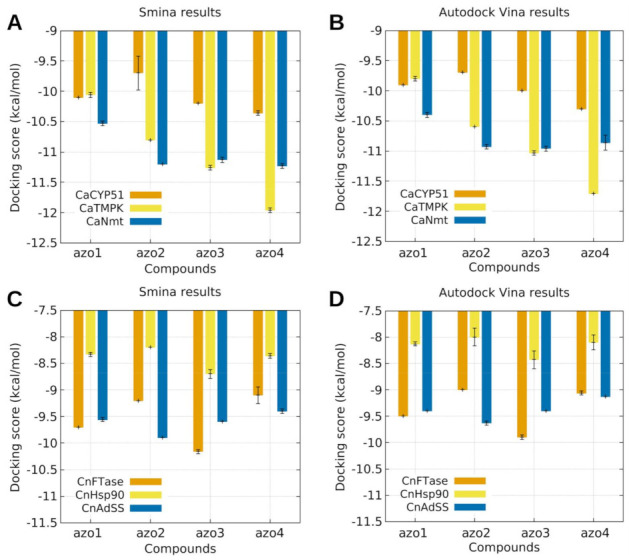
Docking score values obtained for azo1–azo4 ligand series evaluated against the molecular targets *Ca*CYP51, *Ca*TMPK, and *Ca*Nmt (top row), and *Cn*FTase, *Cn*Hsp90, and *Cn*AdSS (bottom row), using the Autodock Vina (left) and Smina (right) programs.

The in silico antifungal evaluation of the azoimine-pyrazole ligands (Azo1 to Azo4) was approached through molecular docking studies against three different molecular targets for each of the selected fungus strains (i.e., *C. albicans* and *C. neoformans*). In the case of the *C. albicans* strain, the targets were the proteins sterol 14-alpha demethylase (*Ca*CYP51), thymidylate kinase (*Ca*TMPK), and N-myristoyltransferase (*Ca*Nmt). Likewise for the *C. neoformans* strain, the molecular targets were the farnesyltransferase protein (*Cn*FTase), the Hsp90 nucleotide binding domain (*Cn*Hsp90), and the adenylosuccinate synthetase protein (*Cn*AdSS). Figure 10 presents the scoring function values obtained from these molecular docking calculations using both the Autodock Vina and Smina programs.

As can be seen in the upper graphs of Figure 10, the Azo1–Azo4 ligand series showed a better docking affinity with the *Ca*NMT and *Ca*TMK proteins. A systematic increase in affinity towards the *Ca*TMK target was observed in the order Azo1 < Azo2 < Azo3 < Azo4, while the nitrated ligands (Azo2–Azo4) exhibited a better docking response with the *Ca*NMT protein than the Azo1 ligand. These results indicate that both the presence and the position of the nitro group in these azo-imine-pyrazole compounds plays a fundamental role in the inhibitory activity of these two proteins.

On the other hand, the results of molecular docking obtained using the *Cn*FTase, *Cn*Hsp90, and *Cn*AdSS proteins from the *C*. *neoformans* strain (graphs below, Figure 10) show that the presence and position of the nitro group in the Azo1–Azo4 series does not generate a marked influence or trend with the corresponding active sites; however, a better protein–ligand interaction is observed for the *Cn*Ftase and *Cn*AdSS proteins with respect to the interaction observed with the *Cn*Hsp90 protein.

#### 2.5.3. Molecular Dynamics (MD) Simulation

To dive into the ligand–protein binding modes, MD simulations and binding free energy (∆G_bind_) calculations were performed for the best molecular docking results. Table 5 shows the free binding energies calculated for the two best ligand–protein couplings per fungus species for each of the compounds Azo1 to Azo4.

**Figure 11:**
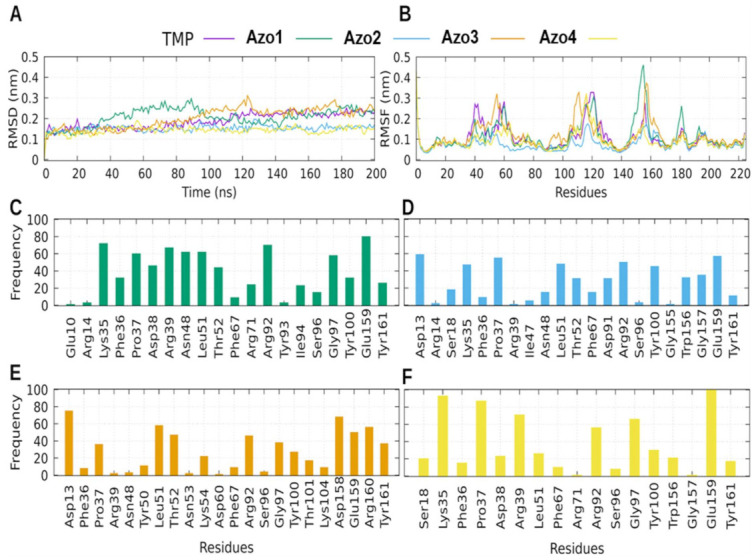
MD results for *Ca*TMPK complexes. (**A**) Protein backbone RMSD as a function of the time for each *Ca*TMPK-ligand complex. (**B**) RMSF for each simulated *Ca*TMPK-ligand complex. Contact frequencies are shown for Azo1 in green (**C**), for Azo2 in blue (**D**), for Azo3 in orange (**E**), and for Azo4 in yellow (**F**).

Figure 11 shows the analysis of the MD trajectories of each protein–ligand complex formed with the *Ca*TMPK protein. This protein represents a promising target for drug design because it presents regions that are unique for *Candida* species and differentiates them from human TMPK. It is also important because of its relevant role in the life cycle of the cell, since it is involved in the synthesis of thymidine which is necessary for DNA replication [31,32].

All the *Ca*TMPK complexes showed low RMSD values throughout 200 ns of MD simulations. Values less than ~0.25 nm suggested that the protein was stable in the simulation. Complexes for Azo2 and Azo4 showed even lower RMSD values than the TMP control (Figure 11A). Among the regions with the greatest flexibility were the Ca-loop (His108 to Asn122) and the LID region (Arg153 to Leu163). It has been reported that the flexibility of Ca-loop does not alter the global topology of the protein; however, the flexibility of the LID region has been reported to alter the catalytic activity of the protein [31].

**Figure 12:**
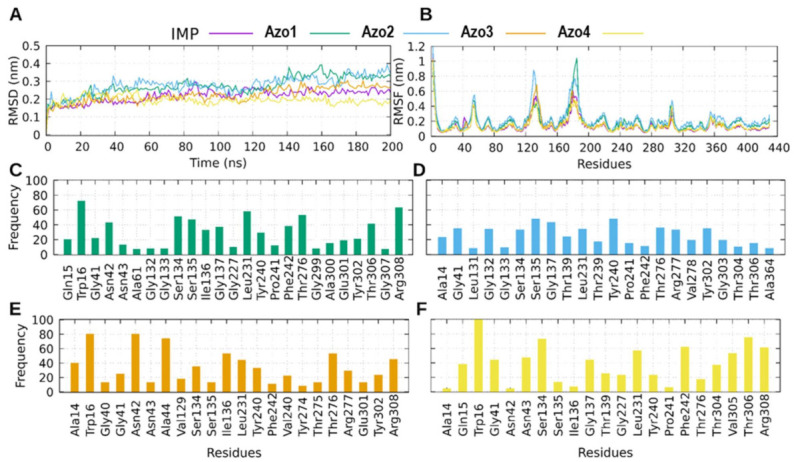
MD results for *Cn*AdSS complexes. (**A**) Protein backbone RMSD as a function of the time for each *Cn*AdSS-ligand complex. (**B**) RMSF for each simulated *Cn*AdSS-ligand complex. Contact frequencies are shown for Azo1 in green (**C**), for Azo2 in blue (**D**), for Azo3 in orange (**E**), and for Azo4 in yellow (**F**).

In the case of ΔG_bind_ calculations for *C. neoformans*, it was observed that neither of the two simulated proteins (*Cn*FTase and *Cn*AdSS) represented the trend of our experimental results. However, it was also observed that the values for ΔG_bind_ were better for the *Cn*AdSS protein, which is why the MD simulations of the protein-ligand complexes formed with this protein were analyzed (Figure 12). AdSS is an important enzyme for the cycle of a cell since it is directly involved with the biosynthesis of ATP [33]. Its inhibition in *C. neoformans* has been shown to result in complete loss of virulence in the murine model [33]. Figure 12A shows the RMSD as a function of time for the complexes with the *Cn*AdSS protein. In all cases the protein behaved in a stable way, since the RMSD reaches values lower than 0.4 nm and behaved homogeneously during the simulations. It is noteworthy to mention that the complexes with Azo4 and Azo3 were the complexes that presented the least variation in RMSD, on average ~0.2 nm and ~0.3 nm for Azo4 and Azo3, respectively. Interestingly, this behavior was preserved when analyzing the flexibility of the protein residues by means of the RMSF graph (orange and yellow lines in Figure 12B). The protein in the presence of Azo4 and Azo3, presented the smallest increases in the flexibility of the residues which were very similar to the behavior obtained for the natural substrate inosine monophosphate (IMP). Conversely, compounds Azo1 and Azo2 presented the highest RMSD (Figure 12A) and RMSF (Figure 12B) values. Three regions with high flexibility have been described in the *Cn*AdSS protein, which in our simulations preserved its flexible character, showing RMSF values between approximately 1 and 0.5 nm (Figure 12B). These regions are from amino acids Arg51 to Thr58, the second from Ile122 to Lys141, and the third from Arg171 to Met194. Of these three regions, our compounds only interacted with the region that goes from Ile122 to Lys141.

Regarding the protein–ligand contacts with the *Cn*AdSS, the compounds shared interactions with residues such as Gly41, Ser134, Ser135, Leu231, Tyr240, Phe242, Thr276, and Arg308, of which Gly41, Ser134, Leu231, and Phe242 are residues highly involved in stabilization of the natural substrate IMP, suggesting that if the protein-inhibitor interaction occurs, our compounds (especially Azo4 and Azo3) could hinder the *Cn*AdSS-IMP interaction. Azo4 presented the best value for the ΔG_bind_. This is related to the fact that their interaction at the IMP binding site was more stable than that of the other compounds as they presented higher contact frequencies (for example with Trp16, Thr306, and Ser134). Additionally, Azo4 presented a unique interaction of almost 60% with the Val305 residue, which in the human orthologue changes to a Threonine. This could favor its binding with respect to the other compounds and allows us to think about specific interactions in *C. neoformans.*

## 3. Materials and Methods

### 3.1. Synthesis

#### 3.1.1. Synthesis of Aminepyrazole Derivatives

The precursor pyrazoles (Pyr in Figure 3) were synthesized following the methodology reported in the literature [34,35]. A measure of 35.18 mmol of 4,4-dimethyl-3-oxopentanenitrile (dopn in Figure 3) and 35.18 mmol of phenylhydrazine (2-nitrophenylhydrazine, 3-nitrophenylhydrazine, 4-nitrophenylhydrazine) were mixed in an Erlenmeyer flask. Then 4 mL of concentrated hydrochloric acid and 32 mL of distilled water were added, and the resulting mixture was heated to 70 °C with constant stirring. After reacting for one hour, 4 mL of concentrated hydrochloric acid was added, and the mixture was heated for an additional hour. The reaction was cooled to room temperature, then placed in an ice bath and neutralized with the careful addition of ammonium hydroxide until a precipitate formed. The solids formed were vacuum filtered and washed initially with water (5 × 50 mL) and then with hexane (2 × 50 mL).

#### 3.1.2. Synthesis of (*E*)-2-hydroxy-4-(phenyldiazenyl)benzaldehyde

(*E*)-2-hydroxy-4-(phenyldiazenyl)benzaldehyde (hpdb in Figure 3) was synthesized by following the reported methodologies [36,37]. Measures of 1.0 g of aniline and 5 mL of a 2:1 mixture of concentrated HCl and water were added to a 100 mL beaker and the resulting solution was stirred at room temperature. After 10 min, the mixture was placed in an ice bath until reaching a temperature close to 0 °C. NaNO_2_ (0.891 g) was then dissolved in distilled water and added dropwise. The reaction mixture was stirred for 30 min to generate (in situ) the diazonium salt. To this solution, an aqueous solution formed by salicylaldehyde (1.313 g), sodium hydroxide (0.430 g), and sodium carbonate (5.703 g) was added dropwise until the formation of a brown precipitate. The mixture was left in an ice bath for 1 h and then filtered under vacuum. Then 5 mL of a mixture of HCl and water (2:1) was added to the solid obtained while stirring for 10 min and then filtered to obtain the hpdb compound.

#### 3.1.3. Synthesis and Physicochemical Properties of Azo-imine-pyrazoles

Azo-imine-pyrazole derivatives Azo1 to Azo4 were synthesized by following reported methodologies [25]. A measure of 0.41 mmol of the corresponding aminepyrazole, 50 mg of compound hpdb (0.41 mmol), and 3 drops of glacial acetic acid were vigorously macerated with a micro spatula until the complete reaction of its precursors. The solids obtained were washed with a cold water/ethanol mixture (5 × 20 mL). Then, the solids were vacuum filtered until dry. Single crystals suitable for X-ray diffraction were obtained by slowly evaporating the solution at room temperature from a solution in toluene for all compounds.

2-((*E*)-((3-(tert-butyl)-1-phenyl-1H-pyrazol-5-yl)imino)methyl)-4-((*E*)-phenyldiazenyl)phenol (**Azo1**). Yellow. Yield: 89%. Anal. Calc. for C_26_H_25_N_5_O: %C 73.74, %H 5.95, %N 16.54, %O 3.78 found: %C 73.68, %H 5.84, %N 16.66, %O 3.65, m.p. 196–198 °C. MS (70 eV) *m/z* (%) 423 [*M*^+^] (100), 424 [*M*^+^ +1] (20.33), 425 [*M*^+^ +2] (3.72), 408 [*M*^+^ -15] (9.66), 366 [*M*^+^ −57] (2.15)). FT-IR ATR (cm^−1^) ν(O-H) 3440, ν(C-H) 3127 (pyrazole), ν(C-H) 3063 (aromatic), ν(C-H) 3005 (imine), ν_as_(C-H) 2960 (*t*-butyl), ν_s_ (C-H) 2862 (*t*-butyl), ν(C=N) 1598 (imine), ν(C=C) 1576 (aromatic), ν(N=N) 1503 (azo), δ_as_(C-H) 1454 (t-butyl), δ_s_(C-H) 1358 (*t*-butyl), ν(C-O) 1281. ^1^H-NMR (300 MHz, acetone-*d_6_*, δ ppm) 1.39 (s, 9H, *t*Bu-H), 6.75 (s, 1H, H-10), 7.09 (d, 1H, ^3^*J* = 8.85 Hz, H-46), 7.45 (t, 1H, ^3^*J* = 7.40 Hz, H-31), 7.54 (m, 1H, H-53), 7.56 (m, 4H, H-29), 7.59 (m, 4H, H-51), 7.69 (d, 2H, ^3^*J* = 7.45 Hz, H-27), 7.91 (d, 2H, H-49), 8.06 (dd, 1H, ^3^*J* = 8.84 Hz, ^4^*J* = 2.32 Hz, H-44); 8.25 (d, 1H, ^4^*J* = 2.25 Hz, H-41), 9.26 (s, 1H, H-37), 12.58 (s, 1H, OH). ^13^C-NMR (75 MHz, acetone-*d*_6_, δ ppm) 29.78 (*t*Bu-C), 34.68 (C-12), 91.12 (C-9), 117.76 (C-45), 119.17 (C-39), 122.44 (C-48), 124.95 (C-26), 127.49 (C-43), 127.62 (C-30), 128.42 (C-40), 129.02 (C-50), 129.21 (C28), 130.54 (C-52), 139.33 (C-25), 145.69 (C-42), 147.35 (C-11), 152.87 (C-47), 162.09 (C-8), 163.12 (C-38), 164.03 (C-36). The atoms were numbered according to Figure 4. UV–Vis, MeCN, λ max nm, (log ε): λ_1_ 200 (4.36), λ_2_ 234 (4.46), λ_3_ 335, λ_4_ 362 (4.44).

2-((*E*)-((3-(tert-butyl)-1-(2-nitrophenyl)-1H-pyrazol-5-yl)imine)methyl)-4-((*E*)-phenyldiazenyl)phenol (**Azo2**). Brown. Yield: 75%. Anal Calc for C_26_H_24_N_6_O_3_: %C 66.65, %H 5.16, %N 17.90, %O 10.24, found: %C 66.74, %H 5.07, %N 18.08, %O 10.13, m.p. 180-182 °C. MS (70 eV) *m/z* (%) 468 [*M*^+^] (82.71), 469 [*M*^+^ +1] (27.72), 470 [*M*^+^ +2] (4.52), 453 [*M*^+^ -15] (3.55), 391 [*M*^+^ −77] (0.77), 77 (100), 57 (42.98). FT-IR ATR (cm^−1^) ν(C-H) 3122 (pyrazole), ν(C-H) 3064 (aromatic), ν(C-H) 3011 (aromatic), ν_as_(C-H) 2964 (*t*-butyl), ν_s_(C-H) 2861 (*t*-butyl), ν(C=N) 1604 (imine), ν(C=C) 1586, ν_as_(NO_2_) 1528, ν(N=N) 1478 (azo), δ_as_(C-H) 1459 (*t*-butyl), δ_s_(C-H) 1362(*t*-butyl), ν_s_(NO_2_) 1352, ν(C-O) 1285. ^1^H-NMR (300 MHz, DMSO-*d_6_*, δ ppm) 1.25 (s, 9H, *t*Bu-H), 6.70 (s, 1H, H-28), 7.05 (s, 1H, H-24), 7.52 (d, 3H, H-43), 7.55 (d, 3H, H-45), 7.68 (t, 1H, H-35), 7.77 (m, 4H, ^3^*J* = 7.12 Hz, H-37), 7.80 (m, 4H, H-39), 7.86 (m, 4H, ^3^*J* = 7.62 Hz, H-32), 7.93 (d, 1H, ^4^*J* = 1.46 Hz, H-26), 8.08 (d, 1H, ^3^*J* = 7.80 Hz, H-30), 8.19 (s, 1H, H-20), 9.16 (s, 1H, H-22). ^13^C-NMR (75 MHz, DMSO-*d_6_*, δ ppm) 30.26 (*t*Bu-C), 32.40 (C-33), 91.58 (C-27), 118.09 (C-23), 120.59 (C-13), 122.68 (C-38), 125.39 (C-29), 125.68 (C-34), 125.92 (C-19), 128.00 (C-25), 129.48 (C-36), 129.89 (C-44), 134.06 (C-31), 131.53 (C-42), 145.12 (C-14), 145.38 (C-11), 149.27 (C-12), 151.92 (C-15), 153.51 (C-18), 161.18 (C-21), 162.29 (C-17), 163.17 (C-16). The atoms were numbered according to Figure 4. UV–Vis MeCN, λ max nm, (log ε): λ_1_ 296 (4.52), λ_2_ 233 (4.62), λ_3_ 335 (4.79), λ_4_ 362 (4.64).

2-((*E*)-((3-(tert-butyl)-1-(3-nitrophenyl)-1H-pyrazol-5-yl)imino)methyl)-4-((*E*)-phenyldiazenyl)phenol (**Azo3**). Yellow. Yield: 80%. Anal. Calc. for C_26_H_24_N_6_O_3_: %C 66.65, %H 5.16, %N 17.90, %O 10.24, found: %C 66.71, %H 5.14, %N 17.87, %O 10.25, m.p. 184-186 °C. MS (70 eV) *m/z* (%) 468 [*M*^+^] (100), 469 [*M*^+^ +1] (29.24), 470 [*M*^+^ +2] (5.19), 453 [*M*^+^ −15] (3.21), 391 [*M*^+^ −77] (2.13), 57 (32.04). FT-IR ATR (cm^−1^) ν(C-H) 3123 (pyrazole), ν(C-H) 3061 (aromatic), ν(C-H) 3011 (aromatic), ν_as_(C-H) 2965 (*t*-butyl), ν_s_(C-H) 2861 (*t*-butyl), ν(C=N) 1604 (imine), ν(C=C) 1586, ν_as_(NO_2_) 1529, ν(N=N) 1478 (azo), δ_as_(C-H) 1460 (*t*-butyl), δ_s_(H-C) 1363 (*t*-butyl), ν_s_(NO_2_) 1352, ν(C-O) 1286. ^1^H-NMR (300 MHz, DMSO-*d_6_*, δ ppm) 1.32 (s, 9H, *t*Bu-H), 6.75 (s, 1H, H-10), 7.15 (d, 1H, ^3^*J* = 8.84 Hz, H-43), 7.55 (m, 3H, H-22), 7.57 (m, 3H, H-51), 7.78 (m, 3H, H-24), 7.80 (m, 3H, H-53), 7.96 (dd, 1H, ^3^*J* = 8.82 Hz, ^4^*J* = 2.44 Hz, H-45), 8.18 (dd, 2H, ^4^*J* = 2.11 Hz, H-20), 8.21 (dd, 2H, H-55), 8.37 (d, 1H, ^4^*J* = 2.43 Hz, H-48), 8.63 (t, 1H, ^4^*J* = 2.08 Hz, H-17), 9.21 (s, 1H, H-39). ^13^C-NMR (75 MHz, DMSO-*d_6_*, δ ppm) 30.32 (*t*Bu-C), 32.87 (C-25), 92.77 (C-9), 118.23 (C-42), 118.25 (C-16), 120.96 (C-40), 121.44 (C-19), 125.21 (C-47), 128.24 (C-44), 129.84 (C-54), 129.94 (C-50), 130.89 (C-52), 130.95 (C-23), 131.41 (C-21), 139.76 (C-15), 145.08 (C-46), 147.97 (C-18), 149.66 (C-12), 151.93 (C-49), 160.14 (C-41), 162.20 (C-38), 162.79 (C-8). The atoms were numbered according to Figure 4. UV–Vis MeCN, λ max nm, (log ε): λ_1_ 196 (4.38), λ_2_ 234 (4.71), λ_3_ 275 (4.49), λ_4_ 335 (4.83), λ_5_ 362 (4.67).

2-((*E*)-((3-(tert-butyl)-1-(4-nitrophenyl)-1H-pyrazol-5-yl)imino)methyl)-4-((*E*)-phenyldiazenyl)phenol (**Azo4**). Yellow. Yield 89%. Anal. Calc. for C_26_H_24_N_6_O_3_: %C 66.65, %H 5.16, %N 17.90, %O 10.24, found: %C 66.62, %H 5.11, %N 17.88, %O 10.17, m.p: 182–184 °C. MS (70 eV) *m/z* (%) 468 [*M*^+^] (100), 469 [*M*^+^ +1] (30.26), 470 [*M*^+^ +2] (5.27), 453 [(*M*^+^ −15] (4.49), 411 [*M*^+^ −57] (0.82), 391 [*M*^+^ −77] (1.87). FT-IR ATR (cm^−1^) ν(C-H) 3120 (pyrazole), ν(C-H) 3063 (aromatic), ν(C-H) 3011 (aromatic), ν_as_(C-H) 2959 (*t*-butyl), ν_s_(C-H) 2863 (*t*-butyl), ν(C=N) 1605 (imine), ν(C=C) 1592, ν_as_(N-O) 1514 (-NO_2_), ν(N=N) 1500 (azo), δ_as_(C-H) 1461 (*t*-butyl), δ_s_(C-H) 1367 (*t*-butyl), ν_s_(N-O) 1333 (-NO_2_), ν(C-O) 1279. ^1^H-NMR (300 MHz, DMSO-*d_6_*, δ ppm) 1.35 (s, 9H, *t*Bu-H), 6.77 (s, 1H, H-24), 7.15 (d, 1H, H-17), 7.85 (d, 2H, ^3^*J* = 6.99 Hz, H-51), 7.53 (m, 3H, H-55), 7.56 (m, 3H, H-53), 7.99 (dd, 3H, ^4^*J* = 2.45 Hz, H-15), 8.03 (dd, 3H, ^3^*J* = 9.16 Hz, H-28), 8.36 (d, 3H, ^4^*J* = 2.45 Hz, H-12), 8.37 (d, 3H, ^3^*J* = 9.14 Hz, H-30), 9.23 (s, 1H, H-19), 11.87 (s, 1H, OH). ^13^C-NMR (75 MHz, DMSO-*d_6_*, δ ppm) 30.43 (*t*Bu-C), 32.87 (C-36), 93.54 (C-23), 118.21 (C-16), 121.41 (C-10), 122.82 (C-50), 124.10 (C-27), 125.07 (C-29), 125.62 (C-11), 128.35 (C-14), 129.90 (C-52), 131.55 (C-54), 144.44 (C-26), 145.49 (C-31), 145.64 (C-13), 150.35 (C-25), 152.35 (C-49), 161.25 (C-18), 162.56 (C-9), 163.67 (C-22). The atoms were numbered according to Figure 4. UV–Vis MeCN, λ max nm, (log ε): λ_1_ 196 (5.27), λ_2_ 234 (5.05), λ_3_ 275 (4.90), λ_4_ 335 (5.36), λ_5_ 362 (5.19).

### 3.2. Analytical and Physicochemical Measurements

All chemicals and solvents used (analytical grade) were purchased from Sigma-Aldrich and Across without further purification. The melting points were determined on a Büchi melting point apparatus. The excitation and emission spectra were obtained in a JASCO 8600 fluorescence spectrophotometer with a FMP-825 microplate reader. Infrared spectra were measured in a Perkin Elmer FT 2000 series spectrophotometer using KBr disks. The NMR spectra were recorded on a Bruker Avance 400 spectrophotometer operating at 400 MHz for ^1^H and at 100 MHz for ^13^C, using tetramethylsilane as the internal standard. The mass spectra were obtained on a SHIMADZU-GCMS 2010-DI-2010 spectrometer equipped with a direct input probe operating at 70 eV. The UV–Vis absorption spectra were obtained in a range of 200–600 nm using a Shimadzu UV–Vis 160 spectrophotometer. Microanalyses were performed on an Agilent CHNS elemental analyzer.

### 3.3. X-ray Diffraction Analysis

Single-crystals suitable for X-ray diffraction of compounds Azo1, Azo2, Azo3, and Azo4 were obtained by slowly evaporating the solutions in toluene at room temperature. Data for the compounds were collected at room temperature (298 K) on a Bruker Apex-II CCD diffractometer using monochromatic graphite MoKα (0.71073Å) radiation. The determination of the cell and the final cell parameters were obtained on all the reflections using the Bruker SAINT software included in the APEX 2 software package. The integration and scaling of the data were carried out using the Bruker SAINT software.

The crystalline structures were solved by direct methods by using the Olex2 program [38] and the models obtained were refined by full–matrix least squares on F2 (SHELXTL–97) [39]. All the hydrogen atoms were placed in calculated positions and refined with fixed individual displacement parameters [Uiso(H) = 1.2Ueq or 1.5Ueq] according to the riding model (C–H bond lengths of 0.93Ǻ and 0.96Ǻ, for methyl and aromatic hydrogen, respectively). There was an exception made for the hydrogen atom from the hydroxyl group which was located farther away from the electronic density. Molecular representations were generated by Diamond [40] and MERCURY 3.9 [41]. Crystallographic data for Azo1, Azo2, Azo3 and Azo4 were deposited at the Cambridge Crystallographic Data Centre with deposition numbers CCDC 2115770 for Azo1, CCDC 2115791 for Azo2, 2115792 for Azo3 and 2115793 for Azo4.

### 3.4. Computational Studies

#### 3.4.1. DFT Calculations

Molecular optimizations and harmonic vibration frequencies were calculated in gas phase in the Gaussian09 suite [42] using the B3LYP hybrid functional in combination with the basis set 6-311++G**. The calculated frequencies were corrected with the scale factor 0.960461 [43] and then interpreted by means of potential energy distributions (PEDs) using the VEDA 4 program [27].

^1^H and ^13^C nuclear magnetic shielding tensors (in ppm) relative to the TMS standard were calculated using the gauge-independent atomic orbital (GIAO) method and the implicit continuum model (PCM) and utilizing the same theoretical optimization levels. Vertical electronic transitions were calculated by the time-dependent density functional theory (TD-DFT) at the same level of theory. For the percentage contribution of the boundary orbitals for each vertical transition the GaussView software was used [44].

Using Koopman’s theorem [45,46,47], the calculated energy of the HOMO and LUMO orbitals was used to determine the molecular descriptors of ionization potential (*I*), electron affinity (*A*), hardness (*η*), smoothness (*σ*), electronegativity (*χ*), potential chemistry (*µ*) and the global electrophilicity index (ω). The mathematical expression for these descriptors together with the corresponding values calculated for the Azo1–Azo4 series are presented in Table S7 (in Supplementary Materials).

#### 3.4.2. Topological Analysis

The study of the topology of the electron density (ρ) in the isolated molecules extracted from the crystals of the compounds Azo1 to Azo4 was carried out in the AIM2000 program [48] using wavefunctions calculated in the Gaussian09 program [42] at the theoretical level B3LYP/6-311++g(2df.2pd). This was performed according to the Quantum Theory of Atoms in Molecules and Crystals (QTAIM-C) developed by Richard Bader and his coworkers [29]. The calculation of critical points (CPs) on the electron density surface was performed using Newton’s method with a maximum number of 120 iterations and a step-size factor of 0.5. The molecular graphs of the isolated molecules were obtained from the calculation of uphill paths from (3,−1) CPs, downhill paths from (3,+1) CPs, and paths connecting (3,−1) and (3,+1) CPs. For each molecule, the completeness of the characteristic set of CPs was verified by means of the Poincaré–Hopf relationship (*n_NCP_* − *n_BCP_* + *n_RCP_* − *n_CCP_* = 1).

#### 3.4.3. Molecular Docking Calculations

For the in silico antifungal evaluation of azoimine-pyrazole derivatives (Azo1–Azo4) against strains of *C. albicans* and *C. neoformans*, molecular docking studies were carried out on six different target receptors extracted from these fungal species. For *C. albicans* we used the sterol 14-alpha demethylase (*Ca*CYP51) protein with PDB code 5FSA [31], the thymidylate kinase protein (*Ca*TMPK) with PDB code 5UIV [49], and the N-myristoyltransferase protein (*Ca*Nmt) with PDB code 1IYL [50]. For C. neoformans we used the farnesyltransferase protein (*Cn*FTase) with PDB code 3SFX [51], the Hsp90 nucleotide binding domain (*Cn*Hsp90) with PDB code 7K9S [52], and the adenylosuccinate synthetase protein (*Cn*AdSS) with PDB code 5I34 [53].

Ligand and receptor preparations were made using Autodock Tools v1.5.6 software [54]. For receptors, the preparation consisted of the elimination of ligands and ions co-crystallized, the addition of polar hydrogens, and the assignment of Kollman charges. In some receptors its main cofactors were conserved in their binding sites, i.e., the P450 heme in CYP51 and the Guanosine-5’-diphosphate (GDP) in Adenylosuccinate synthetase. For ligands, the three-dimensional structure from DFT calculations were used for addition of polar hydrogens and the assignment of Gasteiger charges and torsions.

Molecular docking calculations were performed in triplicate and carried out using the Autodock Vina [55] and Smina [56] software. All the search boxes used had a dimension of 26Å × 26Å × 26Å and were centered on the co-crystallized ligands. A value of 15 for the exhaustiveness parameter was used. To validate our calculations, we did a re-docking of the co-crystallized ligands in each receptor (Figure S26 in Supplementary Materials).

#### 3.4.4. Molecular Dynamics Simulations

To study the behavior of protein-ligand complexes, we performed molecular dynamics (MD) simulations using the Gromacs 2019 package [57]. For this, we use the ligands with the best binding score from the molecular docking calculations. For the protein, the amber99sb-ildn force field was used [58] and the ligands were parameterized using the General Amber Force Field [59] and the ACPYPE tool [60]. All the complexes were solvated in a dodecahedron box using the water model TIP3P [61]. Na^+^ and Cl^−^ ions were added to neutralize the charges of the system and an excess of ions was added to reach a NaCl concentration of 0.15M. Energy minimization and a series of equilibration stages under NVT and NPT conditions (number of particles, volume, and constant temperature) using a temperature of 310 K was carried out by 250 ps applying position restrictions of 1000 kJ mol nm^2^. Then, 4 equilibration stages under NPT conditions (number of particles, pressure, and constant temperature) using a pressure of 1 bar by 250 ps each one, applying gradually decreased position restrictions (1000, 100, 10, 1 kJ mol nm^2^, respectively) were carried out. A total time of 1.25 ns was implemented in our equilibration stage. Finally, a production stage of 200 ns without restrictions and using a time step of 2 fs was carried out. The temperature was controlled using the V-rescale thermostat [62] and a time constant of 0.1 ps. The pressure was controlled using the Parrinello–Rahman barostat [63] and a time constant of 2.0 ps. The non-bonded list was updated every 10 steps. Long-range electrostatic interactions were calculated using the particle mesh Ewald (PME) method and a cutoff radius of 10 Å. Van der Waals interactions were calculated using a Lennard–Jones potential (6–12) using a cut-off radius of 10 Å.

To perform the analysis, the root mean square deviation (RMSD) of the backbone of each protein was calculated using the gmx_rms tool. The root mean square fluctuation (RMSF) of the Cα atoms of each protein was also calculated using the gmx_rmsf tool. Additionally, we calculated the main contacts/interactions between the proteins and each ligand. To do this, we measured the distances between the ligand and all protein residues as a function of time using the gmx_mindist tool. A contact/interaction was considered if the distance was at least 2.5 Å. Then each contact was tracked over time and its frequency was calculated. If the frequency was 100%, it meant that the contact was fully conserved over time. For this analysis, the last 100 ns was used. Finally, to obtain a thermodynamic insight of the protein–ligand interactions, we calculated the binding free-energy (ΔG_bind_) using the molecular mechanics/Poisson–Boltzmann surface area (MM/PBSA) method, available in the gmx_MMPBSA tool [64] which is based on AMBER’s MMPBSA.py [65]. The ΔG_bind_ between a protein and ligand to form a complex can be calculated as seen in the following equations (Equations (1)–(3)) [66]:

ΔG_bind_ = ΔH − TΔS ≈ ΔEMM + ΔG_solvation_ − TΔS (1)

ΔE_MM_ = ΔE_internal_ + ΔE_electrostatic_ + ΔE_van der Waals_ (2)

ΔG_solvation_ = ΔG_PB/GB_ + ΔG_SA_ (3)

where ΔE_MM_ represents the contribution of molecular mechanics energy given by the system in the gas phase, and it can be obtained from the force field implemented in the MD simulations. ΔG_solvation_ represents the solvation energy given by the sum of the polar contribution (ΔG_PB/GB_) and nonpolar contribution (ΔG_SA_). ΔG_PB/GB_ can be obtained using the Poisson–Boltzmann or generalized-Born models, while ΔG_SA_ can be calculated by solvent accessible surface area (SASA). Thus, the sum of ΔE_MM_ and ΔG_solvation_ represents the enthalpic contribution (ΔH) to ΔG_bind_ [66,67]. TΔS represents the conformational entropy upon binding and can be added to refine the obtained values. For ΔG_bind_ calculations, frames from the 100 ns to the end were taken each 100 ps, for a total of 1000 frames per trajectory.

### 3.5. Antifungal Assays

The antifungal activity was determined using the microdilution methods in broth M27-A3 for yeasts and M38-A2 for filamentous fungi following the guidelines of CLSI [30]. The antifungal drug Amphotericin B (Sigma Chemical Co., St Louis, MO, USA) was included as positive control. Azo1-Azo4 compounds and the reference drug were tested against the microorganisms *Candida albicans* ATCC 10231 and *Cryptococcus neoformans* ATCC 32264 from the American Type Culture Collection, (ATCC, Rockville, MD, USA). The inocula of cells were adjusted to 1–5 × 10^3^ cells with colony forming units (CFU)/mL according to CLSI [30].

For minimum inhibitory concentration (MIC) determination, Broth microdilution techniques were performed in 96-well microplates according to the guidelines of the Clinical and Laboratory Standards Institute for yeasts (M27-A3) [30]. For the assay, compound test wells (CTWs) were prepared with stock solutions of each compound in DMSO (maximum concentration ≤ 1%), diluted with RPMI-1640 to final concentrations of 250–3.9 μg/mL. An inoculum suspension (100 μL) was then added to each well (final volume in the well = 200 μL). A growth control well (GCW) (containing medium, inoculum, and the same amount of DMSO used in a CTW, but compound-free) and a sterility control well (SCW) (sample, medium, and sterile water instead of inoculum) were included for each fungus tested. Microtiter trays were incubated in a moist, dark chamber at 30°C for 48 h for both yeasts. The microplates were read in a VERSA Max microplate reader (Molecular Devices, Sunnyvale, CA, USA). Amphotericin B was used as positive control and tests were performed in triplicate. The reduction of growth for each compound concentration was calculated as follows: % of inhibition = 100 − (OD 405 CTW − OD 405 SCW)/(OD 405 GCW − OD 405 SCW). The means ± SEM were used for constructing the dose-response curves representing % inhibition vs. concentration of each compound. Dose-response curves were constructed with SigmaPlot 11.0 software.

MIC50 was defined as the lowest concentration of a compound that showed 50% reduction of the fungal growth control.

## 4. Conclusions

The first four members of a new family of azo-azomethine imines (Schiff bases) with the general formula R^1^-N=N-R^2^-CH=N-R^3^ (R^1^ = Ph, R^2^ = phenol, and R^3^ = pyrazole derivative) were obtained with a high yield (75–89%) by a straightforward and environmentally friendly synthesis method. They were further characterized by means of GC-MS, DRX, UV–Vis, FTIR, and NMR (one- and two-dimensional) physicochemical techniques, as well as by both gas phase and periodical quantum-mechanical electronic DFT methods. These four derivatives, labeled as Azo1 (R^3^ = -((C_3_HN_2_)(*tert*-butyl)(Ph)), Azo2 (R^3^ = -((C_3_HN_2_)(*tert*-butyl)(Ph-NO_2_(*ortho*))), Azo3 (R^3^ = -((C_3_HN_2_)(*tert*-butyl)(Ph-NO_2_(*meta*))), and Azo4 (R^3^ = -((C_3_HN_2_)(*tert*-butyl)(Ph-NO_2_(*para*))), were biologically evaluated as antifungal agents against certified strains of *Candida albicans* (ATCC 10231) and *Cryptococcus neoformans* (ATCC 32264) and demonstrated a moderate-to-high fungicidal activity that was observed to be dependent on the molecular electrophilic character related to the presence and position of the nitro group. In both bioassays, the nitro compound Azo2 (*ortho*-NO_2_ derivative), showed an antifungal activity of less than 50% inhibition, while the compounds Azo3 (*meta*-NO_2_ derivative) and Azo4 (*para*-NO_2_ derivative), in the case of *C. albicans*, and **Azo1** (derivative without NO_2_) and Azo4, in the case of *C. neoformans*, showed inhibitions in ranges of 60–90% and 50–100%, respectively. Antifungal in vitro results were computationally complemented by means of molecular docking and molecular dynamics simulations. In silico studies revealed a high affinity of these azo ligands with two proteins extracted from *C. albicans* (*Ca*TMPK and *Ca*Nmt) and two proteins extracted from *C. neoformans* (*Cn*FTase and *Cn*AdSS). For both *Ca*TMPK and *Ca*Nmt proteins, the best affinity for the azo-azomethine compounds follows the trend of the position of the nitro group as *para* > *meta* > *ortho* > *not-substituted*, which is close to the results observed in the in vitro assays using the *C. albicans* strain. The higher affinity for compounds Azo4 and Azo3 was associated to the fact that the nitro group was more exposed to interact through hydrogen bonds with protein residues located inside the active site. In consensus, modifications of the nitro group on the *ortho* and *meta* positions in azo-pyrazole-azomethines derivatives appear to be a promising strategy in the search for Schiff base compounds with potential antifungal activity.

## Figures and Tables

**Table 1:** Selected experimental and calculated (B3LYP/6-311++g(d,p)) vibrational frequencies in the infrared spectra of new azoimine-pyrazole derivatives Azo1 to Azo4.

Vibrational Mode	Azo1			Azo2			Azo3			Azo4		
	Exp	Theo	%PED ^1^	Exp	Theo	%PED ^1^	Exp	Theo	%PED ^1^	Exp	Theo	%PED ^1^
ν(OH)	3440	3152	99	–	3159	99	–	3157	99	–	3169	99
ν(CH)_pyrazole_	3127	3124	99	3122	3124	99	3123	3125	99	3120	3126	99
ν(CH)_aromatic_	3063	3068	98	3064	3067	98	3065	3068	98	3063	3068	98
ν _asym_(CH_3_)	2960	2968	98	2964	2974	97	2965	2965	99	2959	2966	93
ν_sym_(CH_3_)	2862	2903	97	2861	2914	96	2861	2903	98	2863	2900	99
ν(C=N)_imine_	1598	1583	59	1604	1583	45	1604	1580	57	1605	1580	41
ν_asym_(NO_2_)	–			1528	1523	57	1529	1525	75	1514	1515	55
ν(N=N)_azo_	1478	1477	52	1478	1483	57	1478	1483	65	1481	1483	33
ρ_asym_(CH_3_)	1454	1441	79	1459	1440	84	1460	1445	81	1461	1461	83
ν(CC)_aromatic_	1435	1414	34	1432	1421	26	1433	1436	12	1435	1435	30
ρ_sym_(CH_3_)	1358	1345	59	1362	1367	44	1363	1340	58	1367	1374	24
ν_sym_(NO_2_)	–			1352	1320	67	1352	1315	56	1333	1307	46
ν(CO)	1281	1264	42	1285	1263	77	1286	1259	57	1279	1262	60

^1^ Potential energy distribution percentages (%PDE) calculated using the VEDA 4 program [27].

**Table 2:** Experimental and theoretical (B3LYP/6-311++g(d,p)-SCRF) chemical shifts (in ppm) assignments for the ^1^H-NMR spectra of azoimine-pyrazole derivatives in acetone-*d6* (Azo1) or DMSO-*d6* (Azo2–Azo4) measured at room temperature.

Atom ID	Azo1		Azo2		Azo3		Azo4	
	Exp	Theo ^1^	Exp	Theo ^2^	Exp	Theo ^2^	Exp	Theo ^2^
H-2	12.58	12.47	–	12.10	–	12.56	11.87	12.47
H-10	6.75	6.65	6.70	6.61	6.75	6.71	6.77	6.57
H-tert-butyl	1.39	1.72	1.25	1.61	1.32	1.64	1.35	1.60
H-tert-butyl	1.39	1.11	1.25	1.59	1.32	1.62	1.35	1.55
H-tert-butyl	1.39	1.46	1.25	1.58	1.32	1.60	1.35	1.55
H-tert-butyl	1.39	1.44	1.25	1.54	1.32	1.55	1.35	1.53
H-tert-butyl	1.39	1.31	1.25	1.31	1.32	1.39	1.35	1.36
H-tert-butyl	1.39	1.27	1.25	1.27	1.32	1.37	1.35	1.32
H-tert-butyl	1.39	1.16	1.25	1.25	1.32	1.19	1.35	1.16
H-tert-butyl	1.39	1.14	1.25	1.22	1.32	1.19	1.35	1.16
H-tert-butyl	1.39	1,12	1.25	1.07	1.32	1.16	1.35	1.14
H-27	7.69	7.72	NA	NA	8.63	8.19	8.03	7.85
H-29	7.56	7.65	8.08	8.29	7.55	7.79	8.37	8.65
H-31	7.45	7.69	7.77	7.91	8.18	8.49	NA	NA
H-33	7.56	7.87	7.68	8.16	NA	NA	8.37	8.69
H-35	7.69	7.88	7.86	8.05	7.78	8.89	8.03	8.36
H-37	9.26	9.20	9.16	9.07	9.21	9.25	9.23	9.20
H-41	8.25	8.43	8.19	8.28	8.37	8.35	8.36	8.35
H-44	8.06	8.34	7.93	8.34	7.96	8.41	7.99	8.37
H-46	7.09	7.20	7.05	7.10	7.15	7.14	7.15	7.19
H-49	7.91	8.28	7.80	8.43	7.57	8.33	7.85	8.34
H-51	7.59	7.84	7.55	7.69	7.80	7.74	7.56	7.73
H-53	7.54	7.78	7.52	7.68	8.21	7.79	7.53	7.75
H-55	7.59	7.77	7.55	7.83	7.80	7.92	7.56	7.87
H-57	7.91	8.30	7.80	8.29	7.57	8.27	7.85	8.33

^1^ Values calculated using the PCM implicit solvent model in acetone-d6. ^2^ Values calculated using the PCM implicit solvent model in DMSO-d6. NA: not applicable.

**Table 3:** Experimental and theoretical (B3LYP/6-311++g(d,p)-SCRF) chemical shifts (in ppm) for the ^13^C-NMR spectra of azoimine-pyrazole derivatives in acetone-*d6* (Azo1) or DMSO-*d6* (Azo2–Azo4) measured at room temperature.

Atom ID	Azo1		Azo2		Azo3		Azo4	
	Exp	Theo ^1^	Exp	Theo ^2^	Exp	Theo ^2^	Exp	Theo ^2^
C-8	162.09	169.08	163.17	172.14	162.79	170.24	163.67	171.60
C-9	91.12	95.52	91.58	97.91	92.77	96.94	93.54	99.21
C-11	147.35	123.63	151.92	156.13	149.66	155.75	150.35	158.97
C-12	34.68	38.80	32.40	39.24	32.87	39.38	32.87	39.18
C-13	29.78	27.91	30.26	27,28	30.32	21.77	30.43	27.29
C-17	29.78	30.39	30.26	31.32	30.32	31.01	30.43	31.10
C-21	29.78	32.82	30.26	31.94	30.32	32.23	30.43	31.38
C-25	139.33	146.53	145.38	139.78	139.76	147.23	144.44	152.68
C-26	124.95	129.85	149.27	153.7	130.95	137.24	124.1	127.14
C-28	129.21	132.82	125.39	131.70	131.41	134.17	125.07	130.11
C-30	127.62	132.08	129.48	133.15	121.44	126.65	145.49	152.17
C-32	129.21	134.15	125.68	141.07	147.97	155.75	125.07	131.04
C-34	124.95	129.68	134.06	134.19	118.25	124.88	124.1	128.04
C-36	164.03	167.11	161.18	170.19	162.2	167.77	161.25	169.36
C-38	163.12	171.70	162.29	172.04	160.14	172.24	162.56	172.60
C-39	119.17	124.62	120.59	123.07	120.96	123.90	121.41	123.46
C-40	128.42	122.93	125.92	145.62	125.21	145.37	125.62	145.85
C-42	145.69	151.65	145.12	152.22	145.08	152.04	145.64	152.17
C-43	127.49	145.25	128.00	125.64	128.24	125.43	128.35	125.86
C-45	117.76	121.24	118.09	122.05	118.23	122.33	118.21	122.56
C-47	152.87	158.53	153.51	159.13	151.93	158.73	152.35	158.97
C-48	122.44	139.40	122.68	117.85	129.94	117.32	122.82	117.48
C-50	129.02	134.21	129.89	134.27	130.89	133.73	129.9	134.09
C-52	130.54	137.40	131.53	137.18	129.84	137.48	131.55	137.42
C-54	129.02	133.53	129.89	134.55	130.89	134.24	129.9	134.09
C-56	122.44	117.47	122.68	139.78	129.94	139.21	122.82	117.48

^1^ Values calculated using the PCM implicit solvent model in acetone-d6.^2^ Values calculated using the PCM implicit solvent model in DMSO-d6.

**Table 4:** Amount and type (ω,σ) of critical points detected on the ρ topology of the Azo1 to Azo4 molecules as they appear in the crystalline package and their corresponding Poincaré–Hopf relationship (PHR).

Molecule	(3,−3)	(3,−1)	(3,+1)	(3,+3)	Total	PHR
**Azo1**	57	61	5	0	123	1
**Azo2**	59	66	9	1	135	1
**Azo3**	59	64	6	0	129	1
**Azo4**	59	64	6	0	129	1

**Table 5:** ΔG_bind_ (kcal/mol) for the best couplings ligand–protein using Azo1 to Azo4 derivatives and molecular targets obtained from *C. albicans* and *C. neoformans* fungus species.

Molecule	*C. albicans*		*C. neoformans*	
	*Ca*TMPK	*Ca*NmT	*Cn*FTase	*Cn*AdSS
**Azo1**	−35.882	−33.111	−15.183	−31.599
**Azo2**	−37.317	−33.627	−15.484	−33.300
**Azo3**	−48.055	−43.832	−19.861	−41.357
**Azo4**	−37.658	−32.734	−12.993	−42.273

## Supplementary Materials

The following are available online. Figure S1: Fragmentation patterns of compound Azo1, Figure S2: Fragmentation patterns of compound Azo2, Figure S3: Fragmentation patterns of compound Azo3, Figure S4: Fragmentation patterns of compound Azo4, Figure S5: Correlation plots of calculated versus experimental frequencies (cm^−1^) for the compounds Azo1 to Azo4, Figure S6: The 1D-NMR spectra of compound Azo1 in acetone-d6: ^1^H and ^13^C, Figure S7: 1D-NMR spectra of compound Azo2 in DMSO-d6: ^1^H and ^13^C, Figure S8: The 1D-NMR spectra of compound Azo3 in DMSO-d6: ^1^H and ^13^C, Figure S9: The 1D-NMR spectra of compound Azo4 in DMSO-d6: ^1^H and ^13^C, Figure S10: DEP-135-NMR spectra of compound Azo1 in acetone-d6, Figure S12: DEP-135-NMR spectra of compound Azo4 in DMSO-d6, Figure S13: HSQC-NMR spectra of compound Azo1 acetone-d6, Figure S14: HMBC-NMR spectra of compound Azo1 in acetone-d6, Figure S15: HSQC-NMR spectra of compound Azo2 in DMSO-d6, Figure S16: HMBC-NMR spectra of compound Azo2 in DMSO-d6, Figure S17: HSQC-NMR spectra of compound Azo3 in DMSO-d6, Figure S18: HMBC-NMR spectra of compound Azo3 in DMSO-d6, Figure S19: HSQC-NMR spectra of compound Azo4 in DMSO-d6, Figure S20: HMBC-NMR spectra of compound Azo4 in DMSO-d6, Figure S21: Correlation plots of calculated versus experimental ^1^H-NMR chemical displacements (in ppm) for the compounds Azo1 to Azo4, Figure S22: Correlation plots of calculated versus experimental ^13^C-NMR chemical displacements (in ppm) for the compounds Azo1 to Azo4, Figure S23: Re-docking results, Table S1: Experimental and theoretical electronic absorption wavelengths and main orbital contributions for compounds Azo1 to Azo4, Table S2: Main crystallographic data and refinement parameters for the compounds Azo1, Azo2, Azo3 and Azo4, Table S3: Main BCPs topological properties in Azo1 molecule, Table S4: Main BCP topological properties in Azo2 molecule, Table S5: Main BCP topological properties in Azo3 molecule, Table S6: Main BCP topological properties in Azo4 molecule.

## Sample Availability

Samples of the compounds Azo1, Azo2, Azo3 and Azo4, as well as samples of their synthetic precursors (Pyr1 to Pyr4, and hpdb) are available from the authors.
