# Supplementary material for: Synthesis, Structural Characterization, and In Vitro and In Silico Antifungal Evaluation of Azo-Azomethine Pyrazoles (PhN2(PhOH)CHN(C3N2(CH3)3)PhR, R = H or NO2)
Source: Molecules. 2021 Dec 8;26(24):7435. doi: 10.3390/molecules26247435 (PMC8708670; doi:10.3390/molecules26247435)
Supplement: Supplementary file 1 [file molecules-26-07435-s001.zip › molecules-1444359-supplementary.pdf]

molecules

Article

Supporting information

Synthesis, structural characterization, and *in vitro* and *in silico* antifungal evaluation of azo-azomethine pyrazoles ($PhN_2(PhOH)CN(C_3N_2(CH_3)_3)PhR$, R = H or NO_2)

Dorancelly Fernandez [1], Andrés Restrepo-Acevedo [2], Cristian Rocha-Roa [3,4], Ronan Le Lagadec [2], Rodrigo Abonia [5], Susana A. Zacchino [6], Jovanny A. Gómez Castaño [7] and Fernado Cuenú-Cabezas [1,*]

1. Laboratorio de Química Inorgánica y Catálisis, Programa de Química, Universidad del Quindío, Carrera 15, Calle 12 Norte, Armenia, Colombia
2. Instituto de Química UNAM, Circuito Exterior s/n, Ciudad Universitaria, 04510 Ciudad de México, México
3. Grupo GEPAMOL, Centro de Investigaciones Biomédicas, Universidad del Quindío, Carrera 15, Calle 12 Norte, Armenia, Colombia
4. Biophysics of Tropical Diseases, Max Planck Tandem Group, Universidad de Antioquia, Medellín, Colombia
5. Departamento de Química, Universidad del Valle, Calle 13 No. 100-00, A.A. 25360, Cali, Colombia
6. Área Farmacognosia, Facultad de Ciencias Bioquímicas y Farmacéuticas, Universidad Nacional de Rosario (UNR), Suipacha 531, 2000 Rosario, Argentina
7. Grupo Química-Física Molecular y Modelamiento Computacional (QUIMOL®), Facultad de Ciencias, Universidad Pedagógica y Tecnológica de Colombia (UPTC), Avenida Central del Norte, Tunja, Boyacá, 050030, Colombia; grupo.quimol@uptc.edu.co

* Correspondence: fercuenu@uniquindio.edu.co

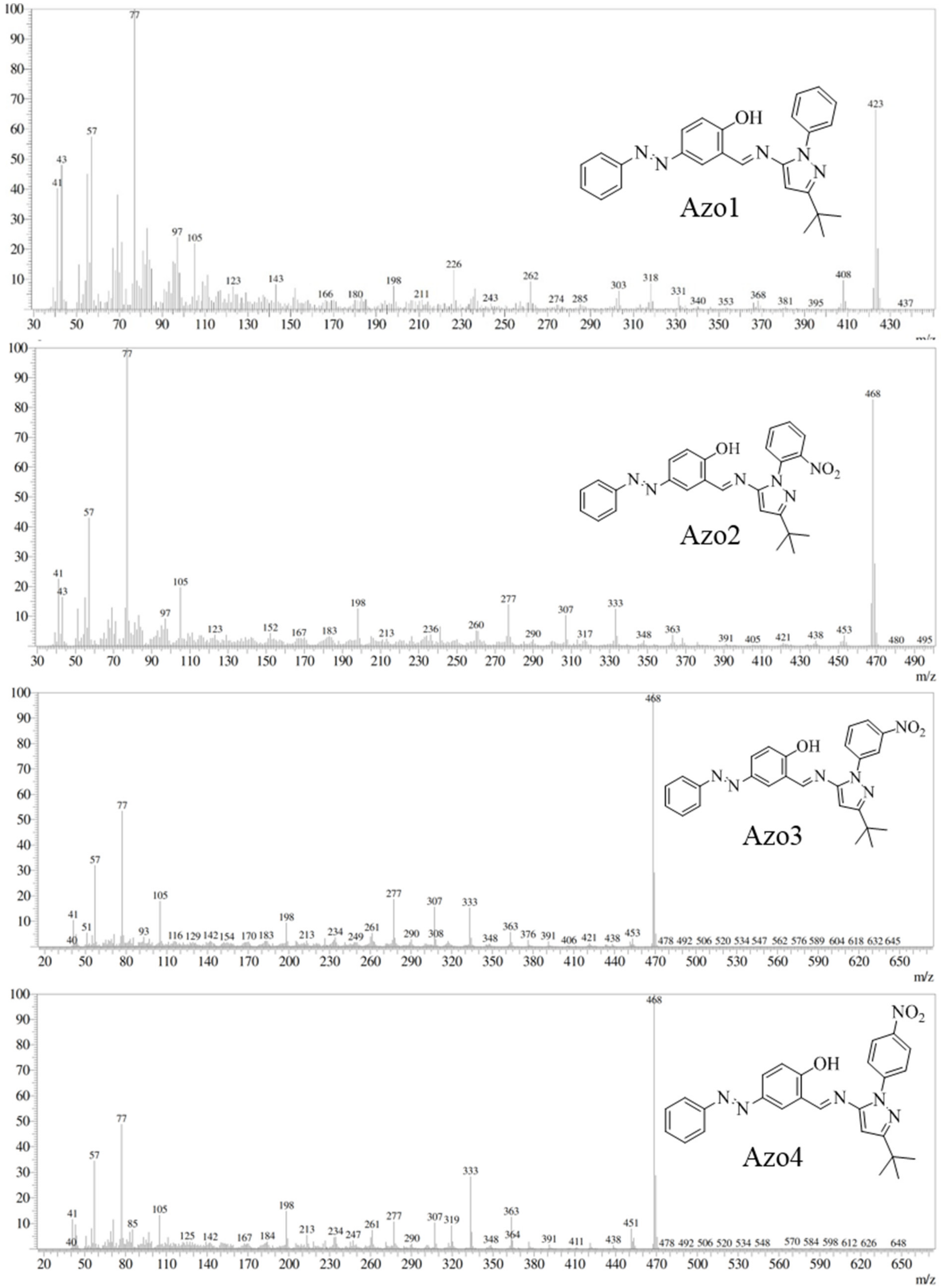

Figure S1. Electron-impact mass fragmentation spectra for azoimine-pyrazole derivatives.

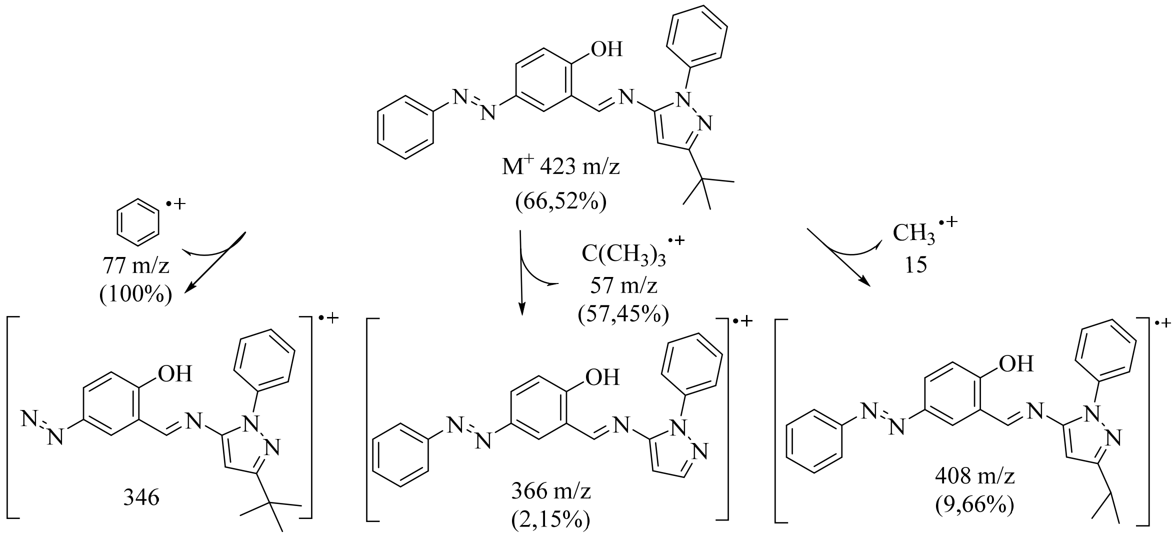

Figure S2. Fragmentation patterns of compound **Azo1**.

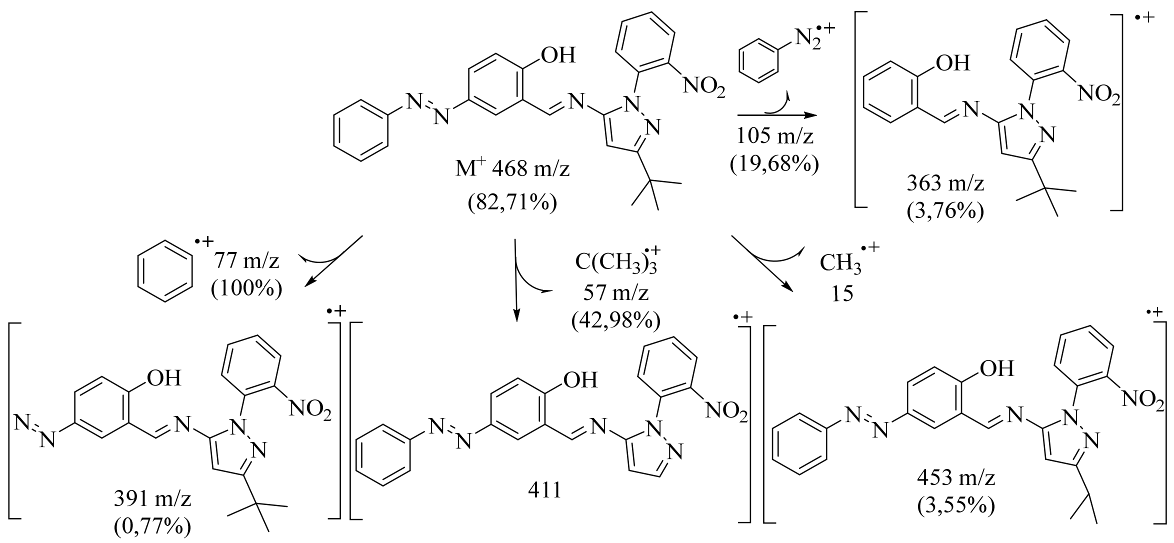

Figure S3. Fragmentation patterns of compound **Azo2**.

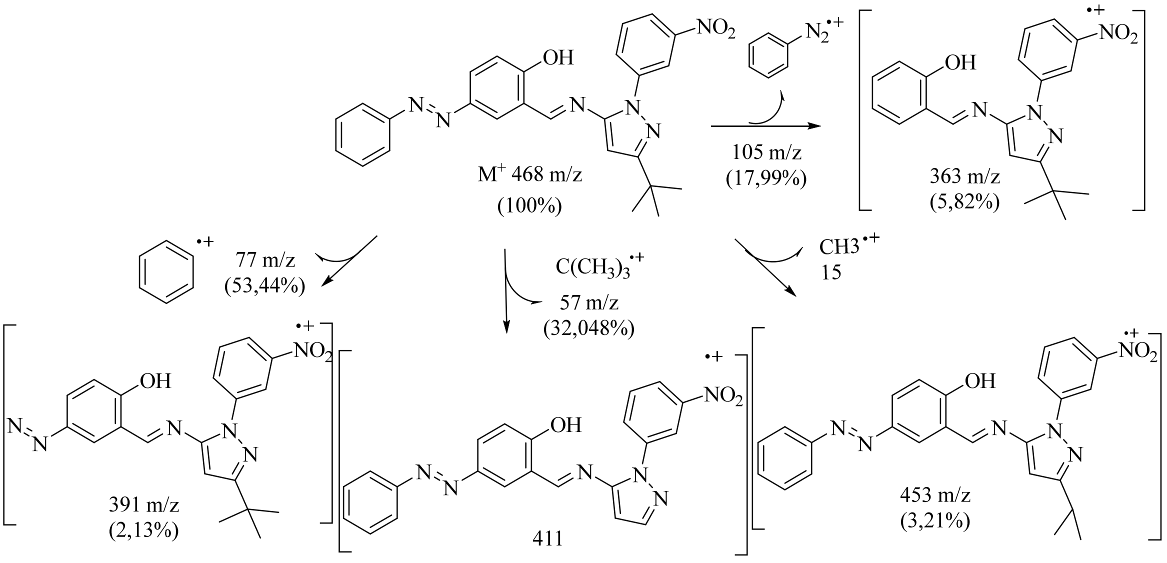

Figure S4. Fragmentation patterns of compound **Azo3**.

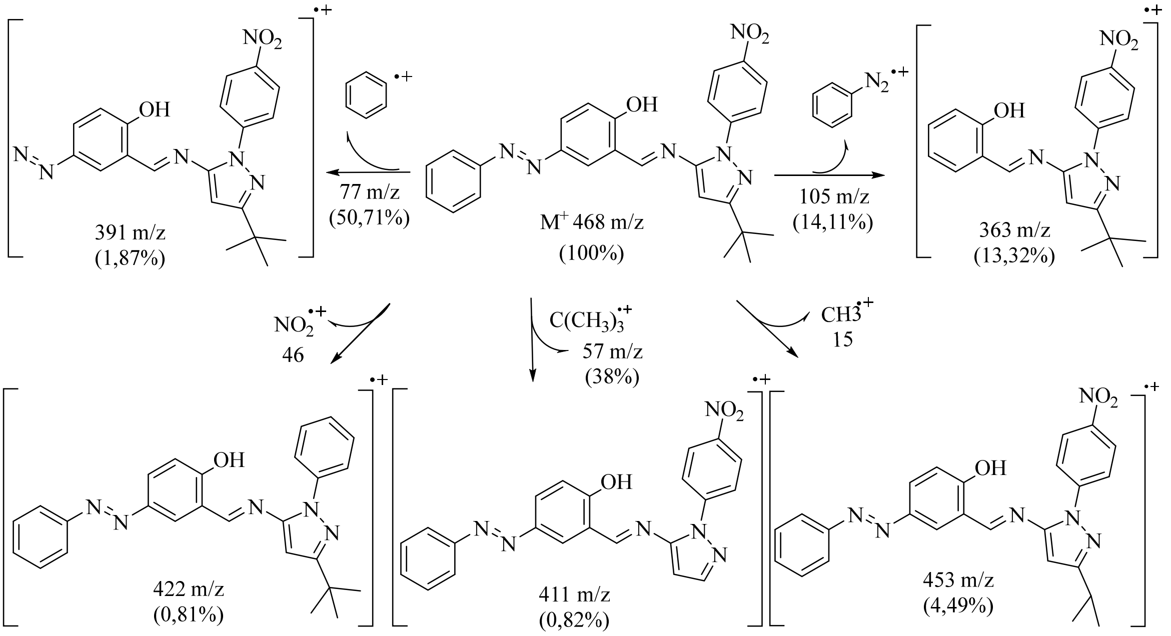

Figure S5. Fragmentation patterns of compound **Azo4**.

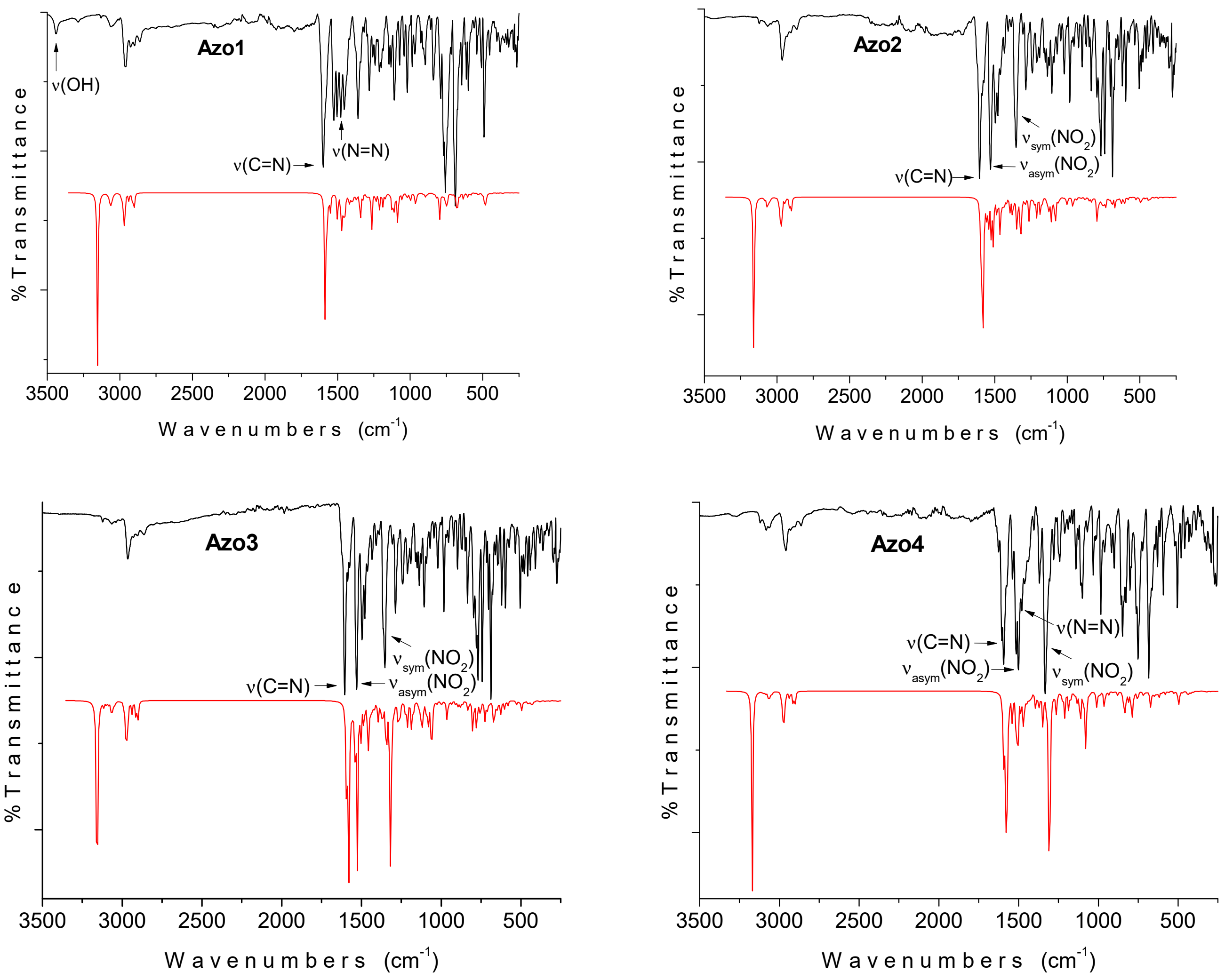

Figure S6. Infrared spectra for the derivative series **Azo1** to **Azo2** in the 3500-250 cm^{-1} range. In black: experimental FTIR spectra measured at a resolution of 4.0 cm^{-1}. In red: theoretical infrared spectra calculated at the B3LYP/6-311++g(d,p) approximation level

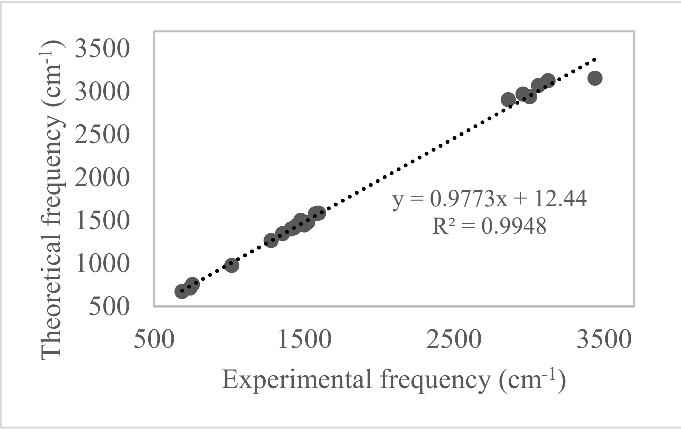

Azo1

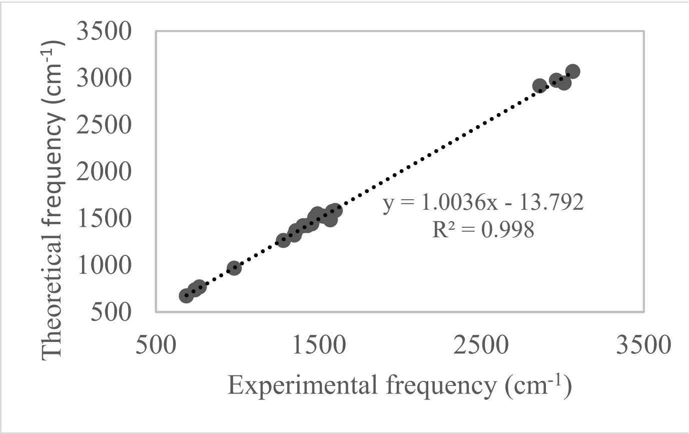

Azo2

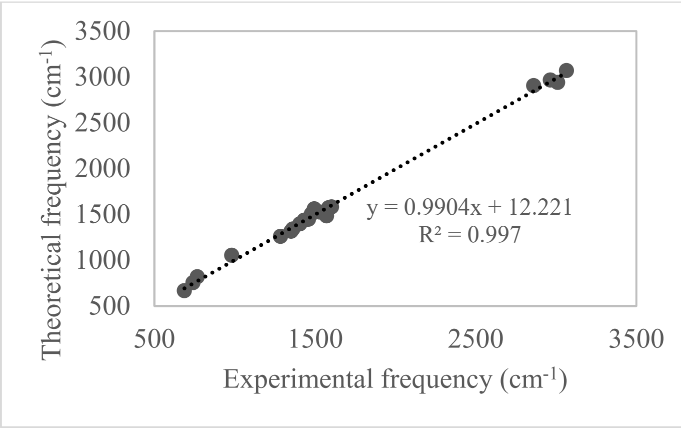

Azo3

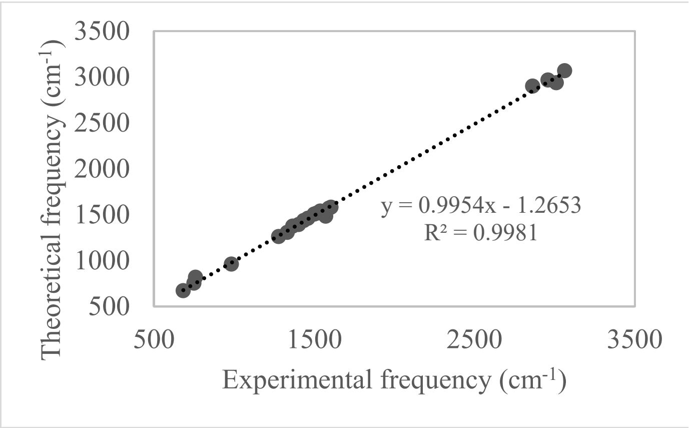

Azo4

Figure S7. Correlation plots of calculated versus experimental frequencies (cm^{-1}) for the compounds **Azo1** to **Azo4**.

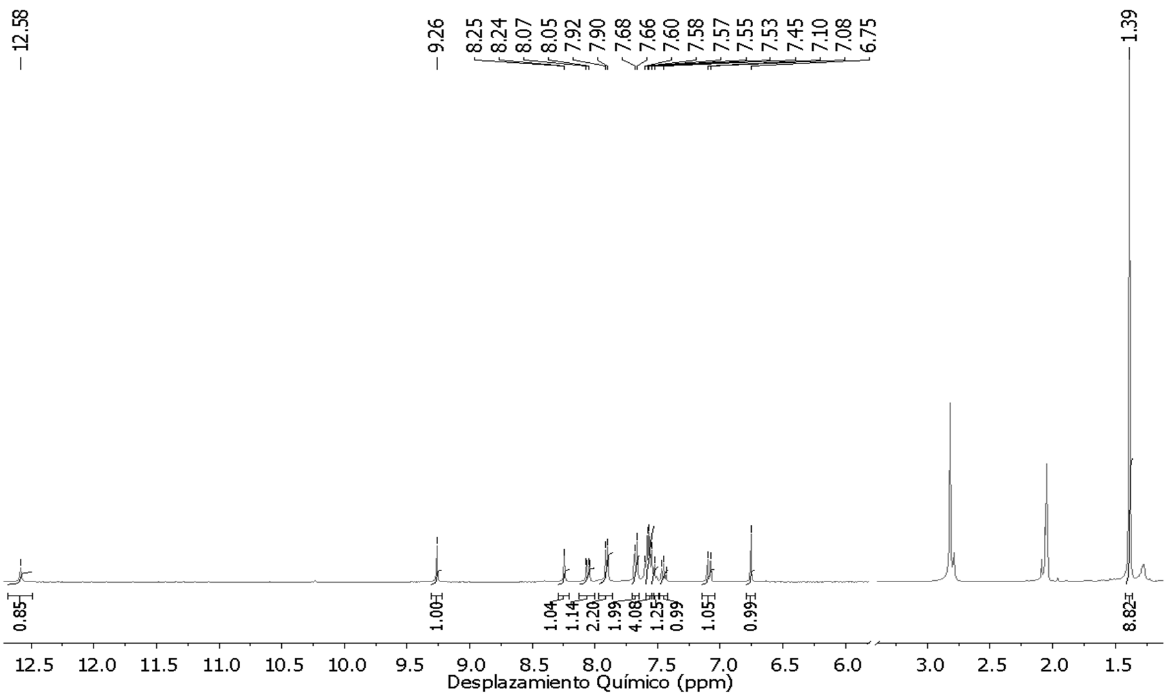

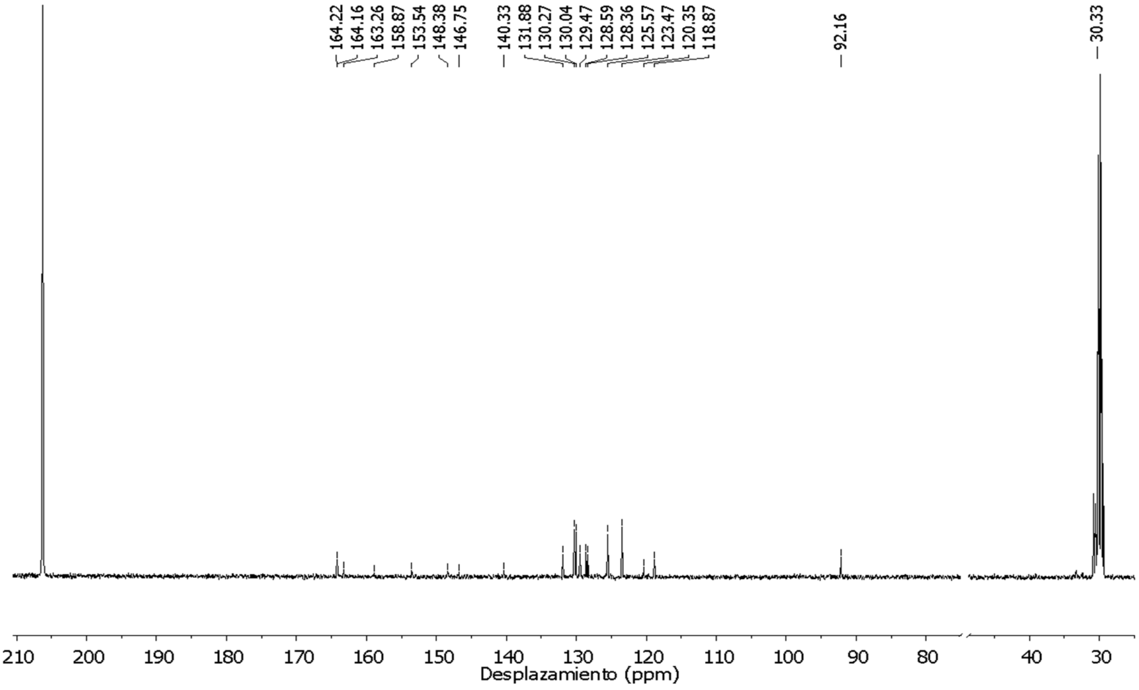

Figure S8. 1D-NMR spectra of compound **Azo1** in acetone-*d6*: ^{1}H (top) and ^{13}C (botton).

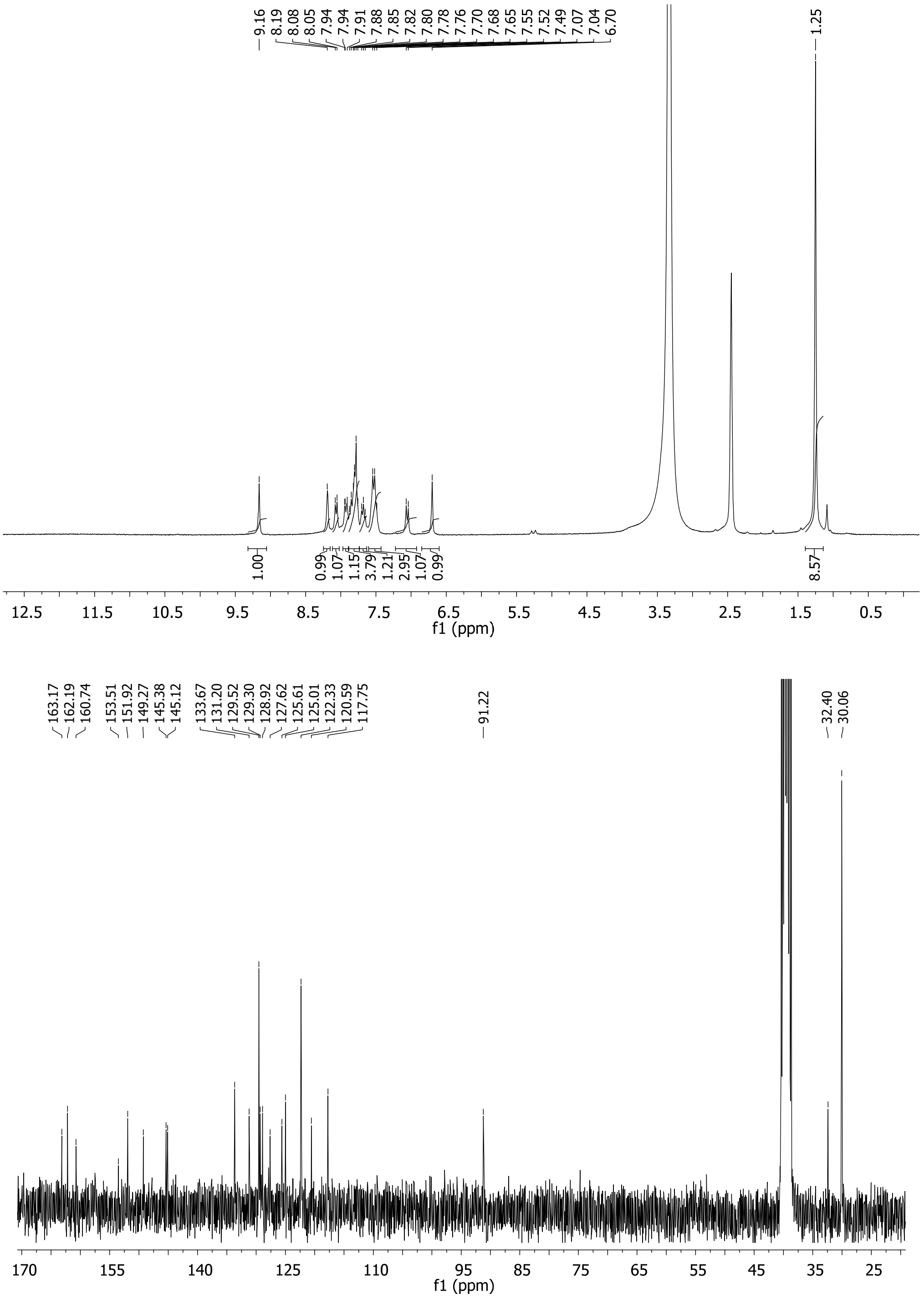

Figure S9. 1D-NMR spectra of compound **Azo2** in DMSO-*d6*: ^{1}H (top) and ^{13}C (botton).

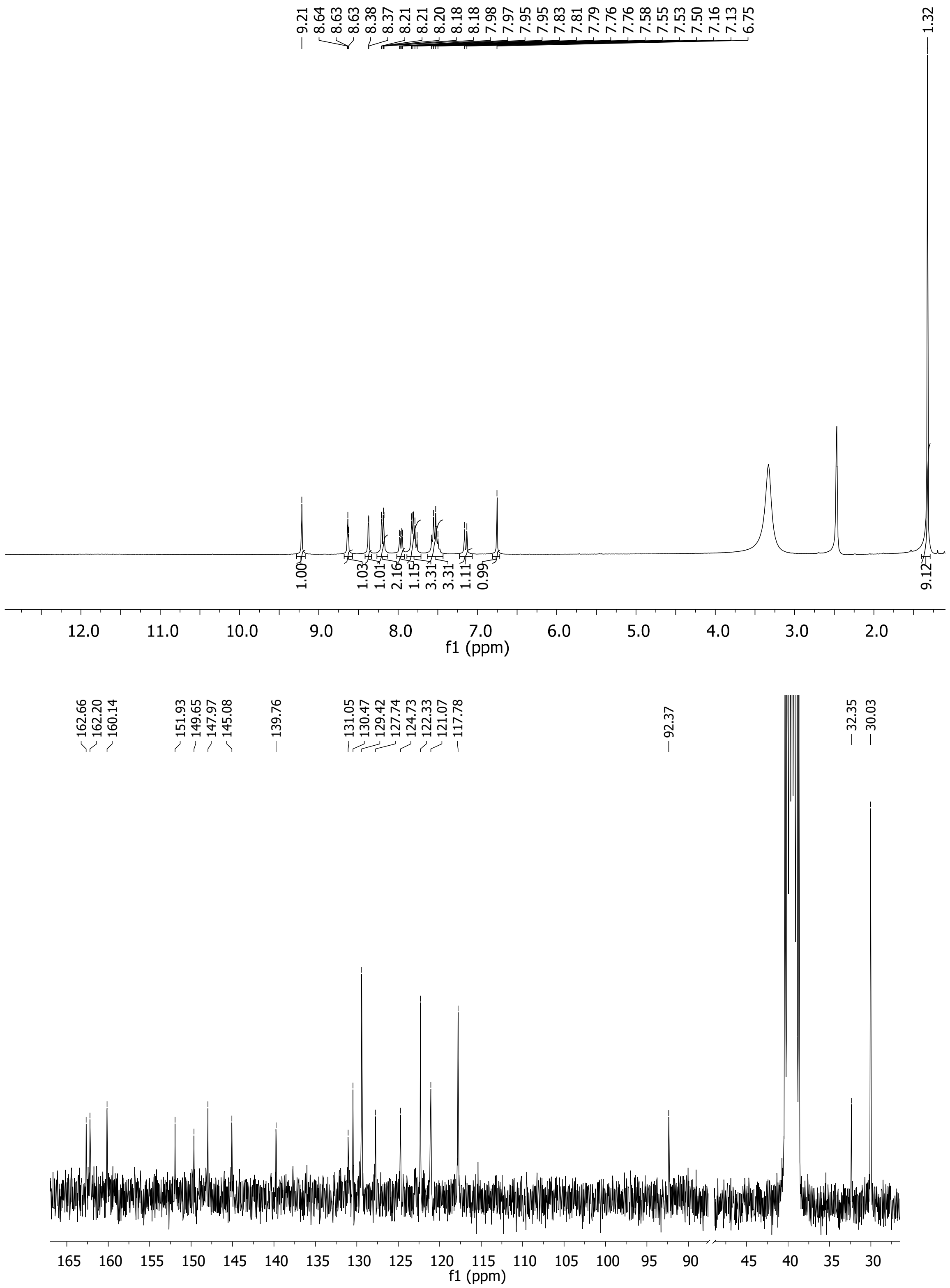

Figure S10. 1D-NMR spectra of compound **Azo3** in DMSO-*d6*: ^{1}H (top) and ^{13}C (botton).

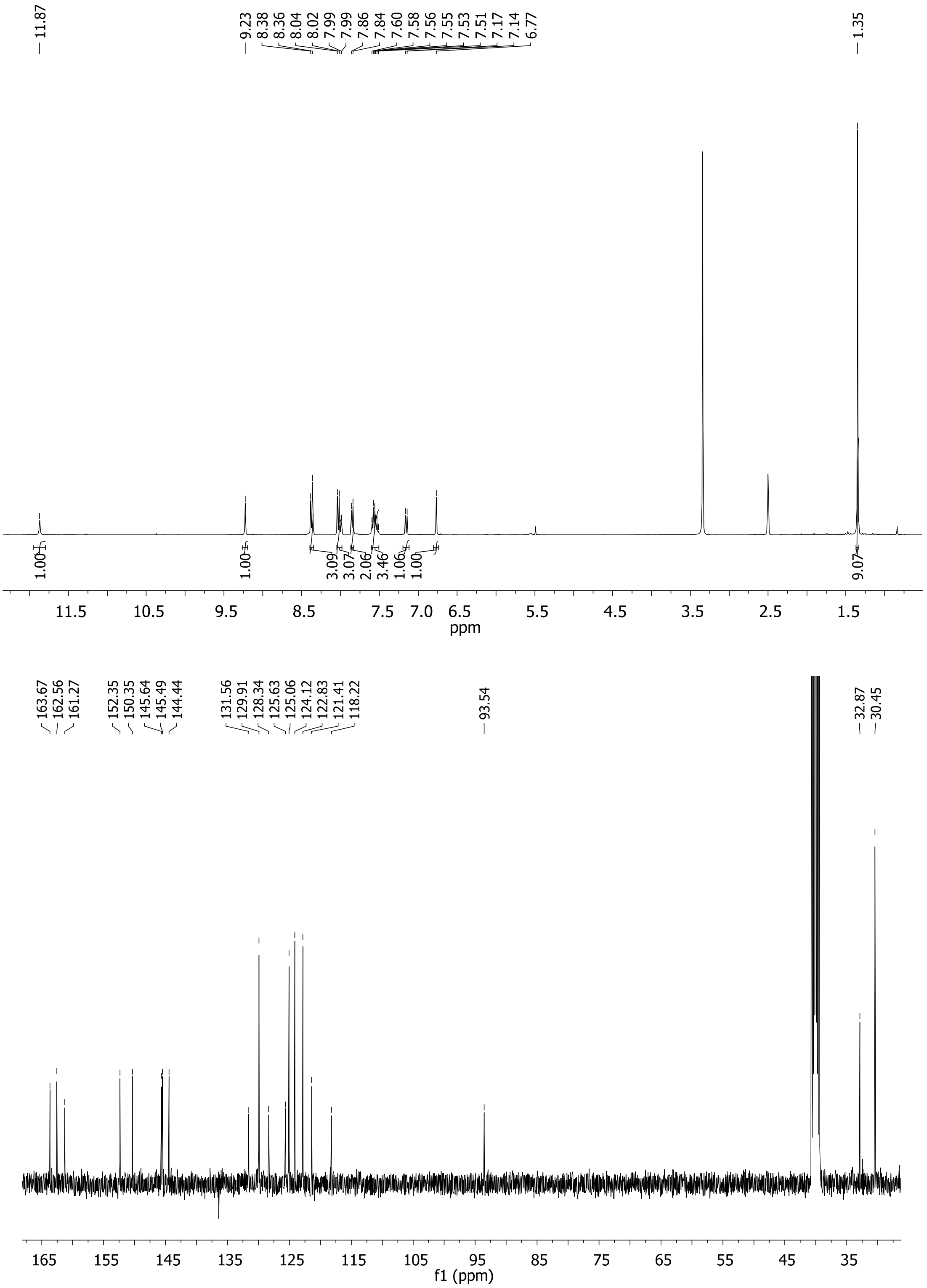

Figure S11. 1D-NMR spectra of compound **Azo4** in DMSO-*d6*: ^{1}H (top) and ^{13}C (botton).

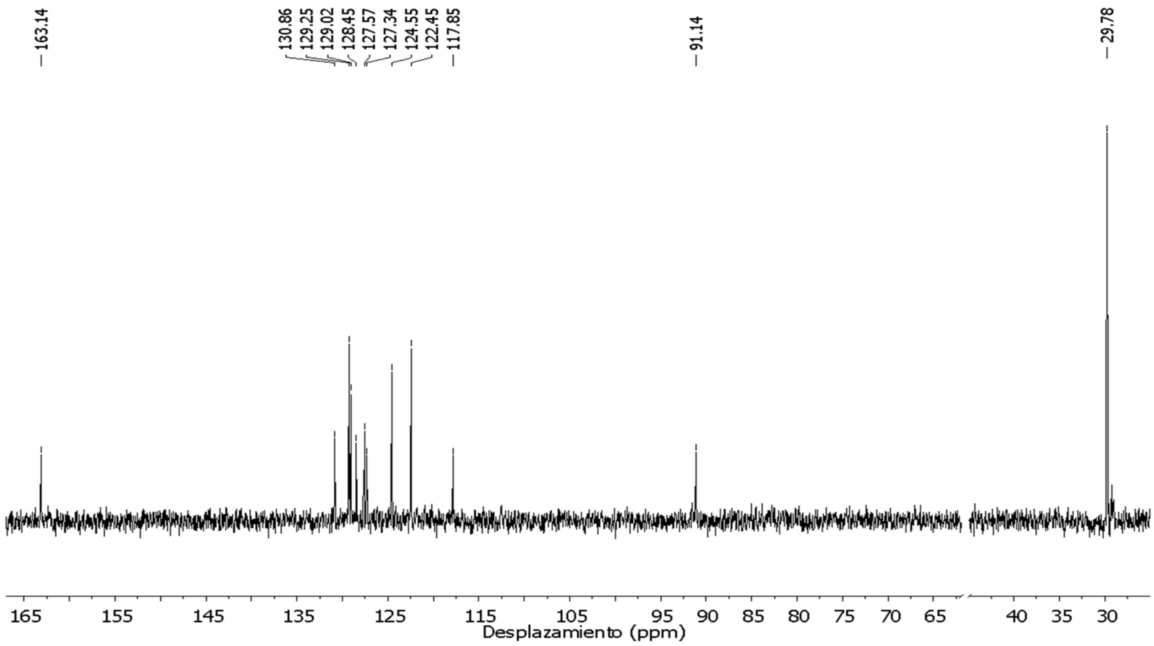

Figure S12. DEP-135-NMR spectra of compound **Azo1** in acetone-*d6*.

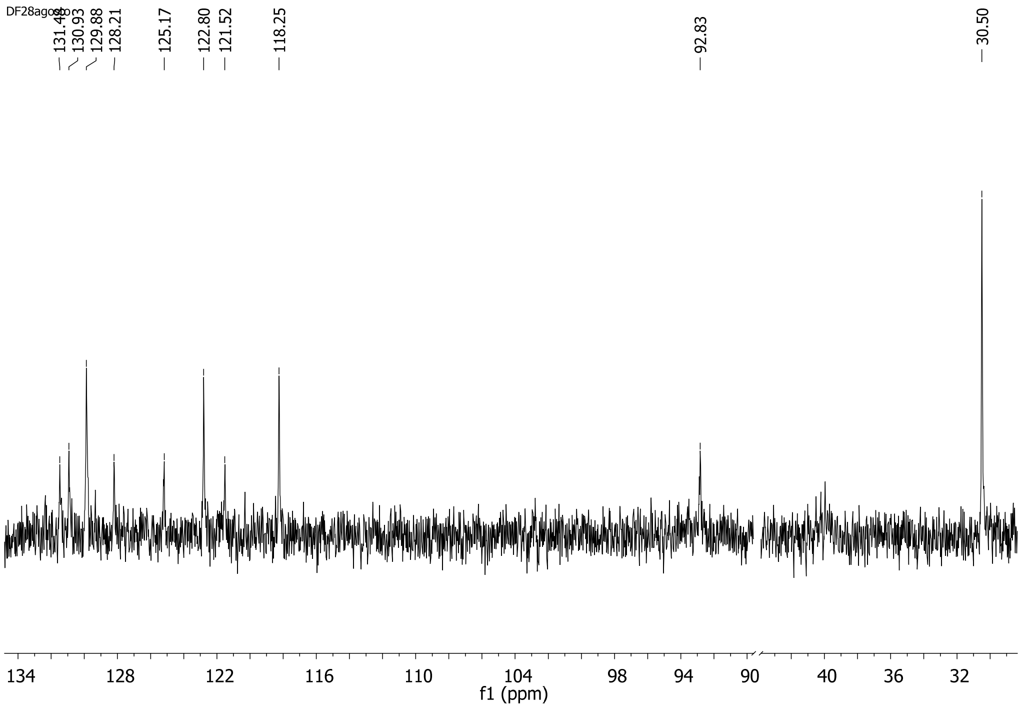

Figure S13. DEP-135-NMR spectra of compound **Azo3** in DMSO-*d6*.

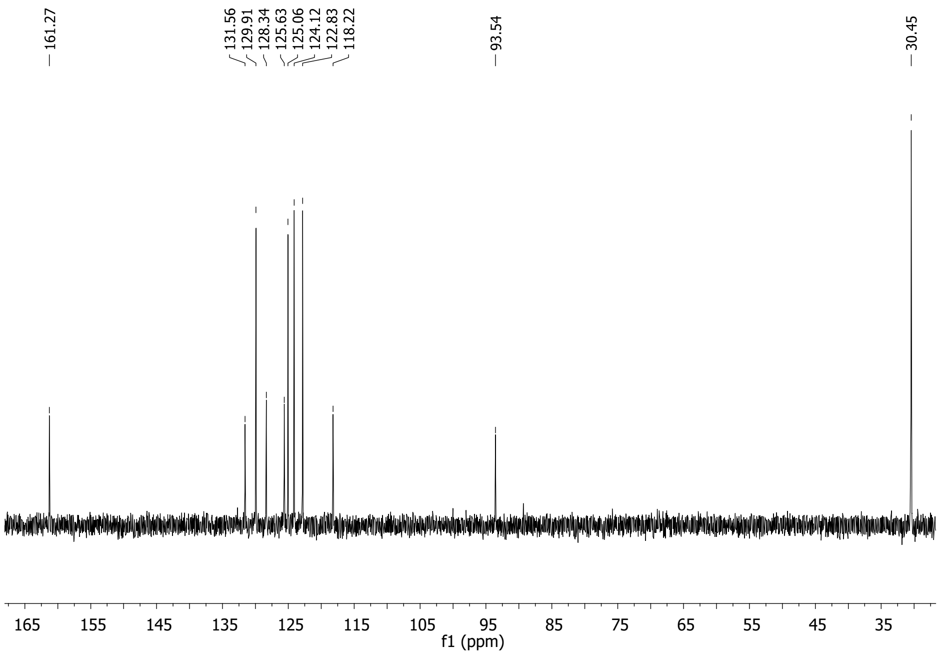

Figure S14. DEP-135-NMR spectra of compound **Azo4** in DMSO-*d6*.

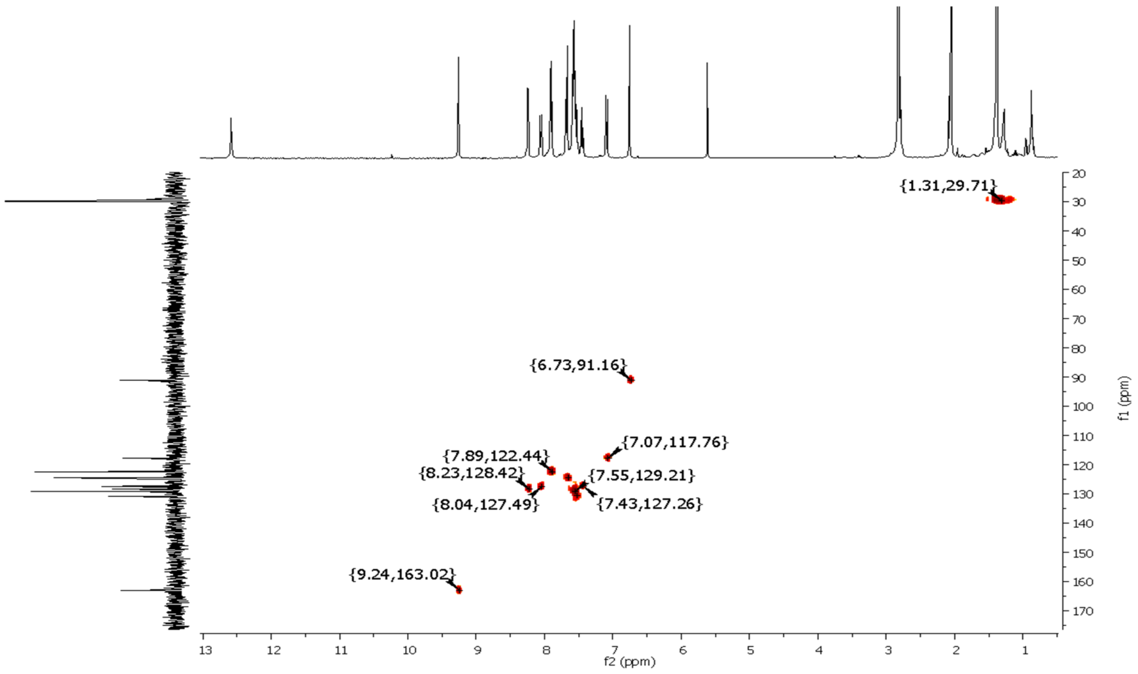

Figure S15. HSQC-NMR spectra of compound **Azo1** acetone-*d6*.

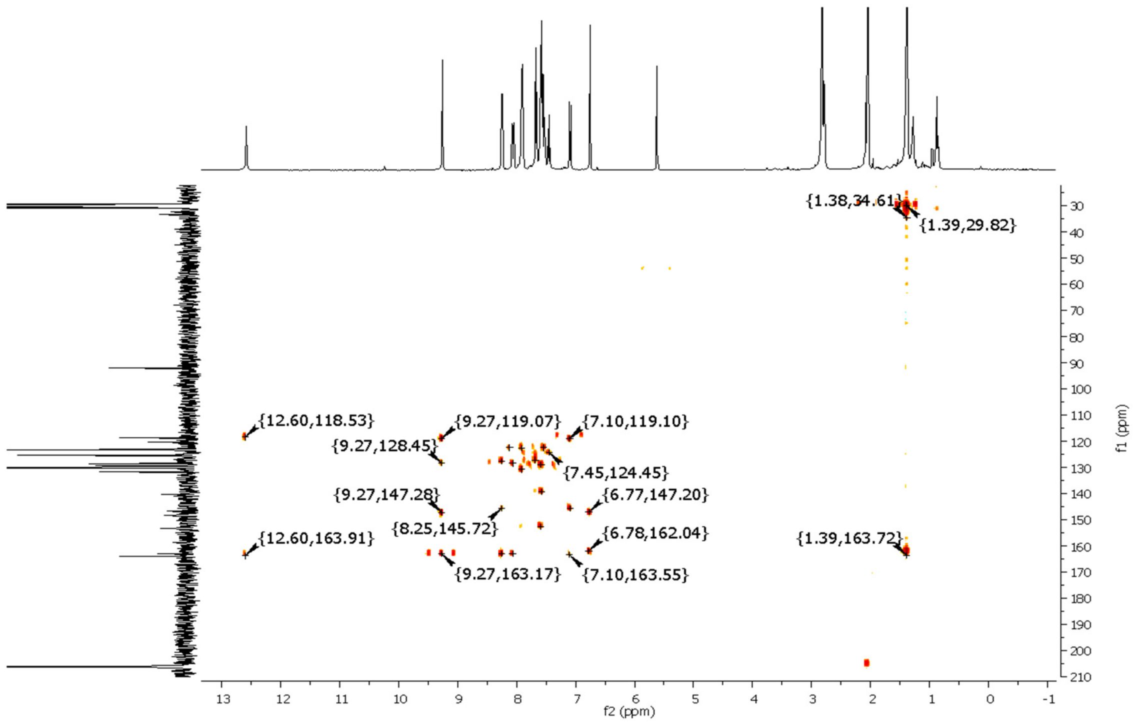

Figure S16. HMBC-NMR spectra of compound **Azo1** in acetone-*d6*.

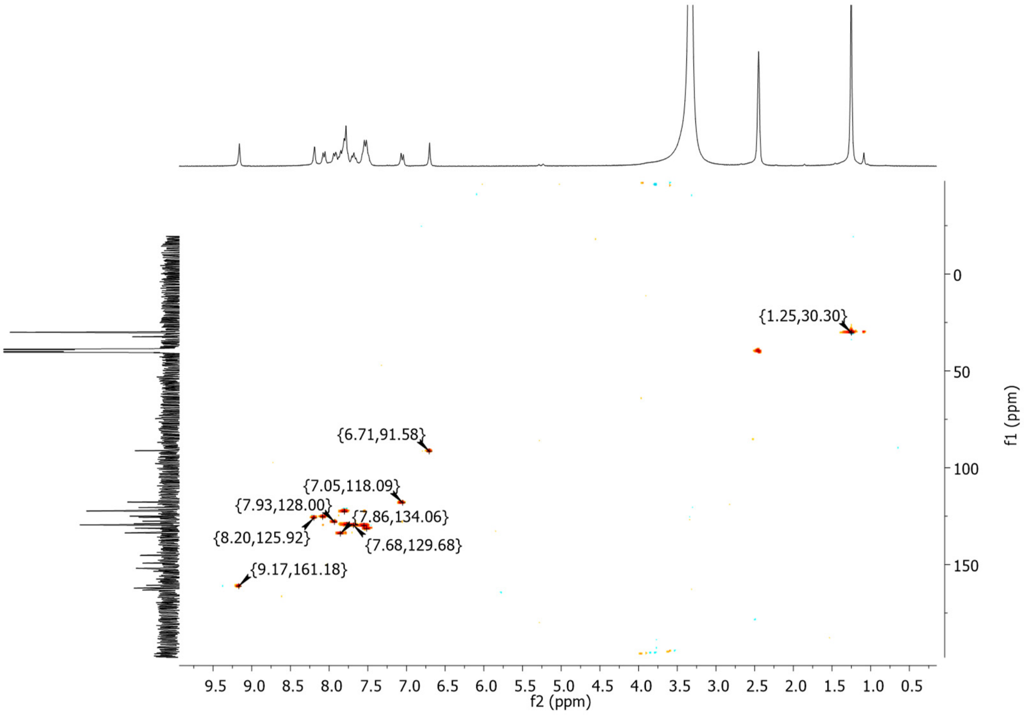

Figure S17. HSQC-NMR spectra of compound **Azo2** in DMSO-*d6*.

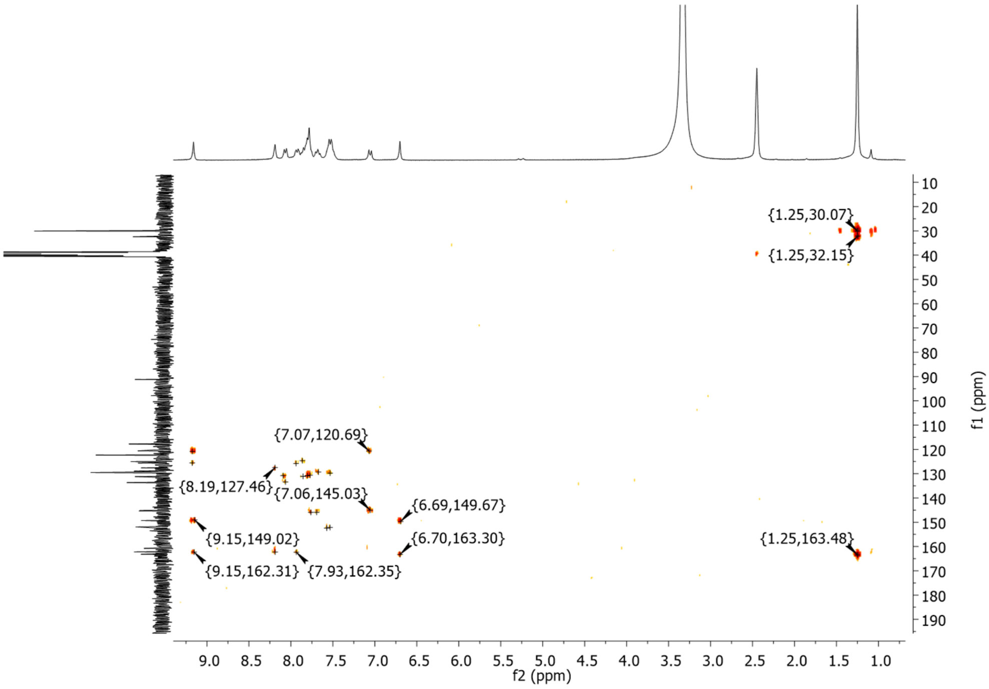

Figure S18. HMBC-NMR spectra of compound **Azo2** in DMSO-*d6*.

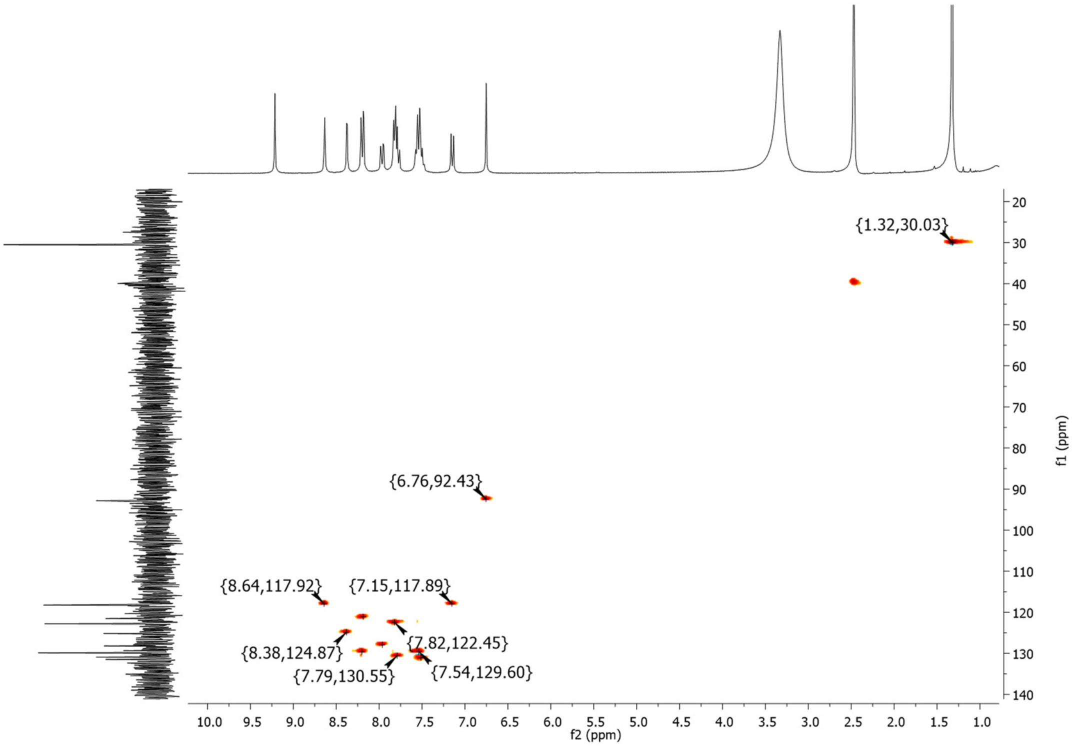

Figure S19. HSQC-NMR spectra of compound **Azo3** in DMSO-*d6*.

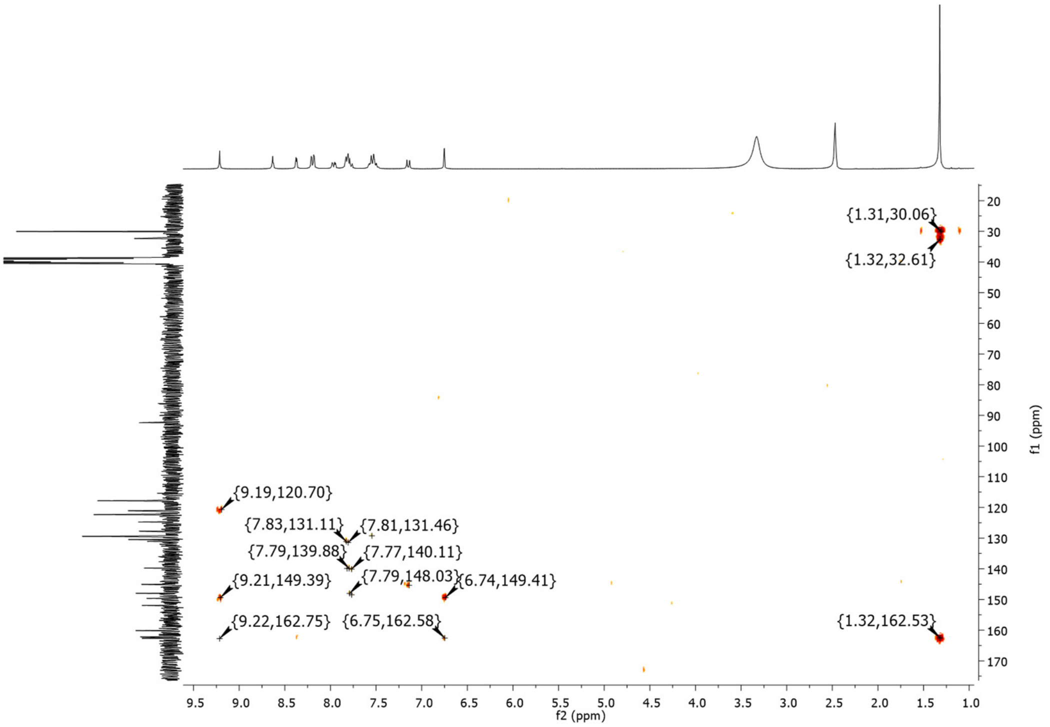

Figure S20. HMBC-NMR spectra of compound **Azo3** in DMSO-*d6*.

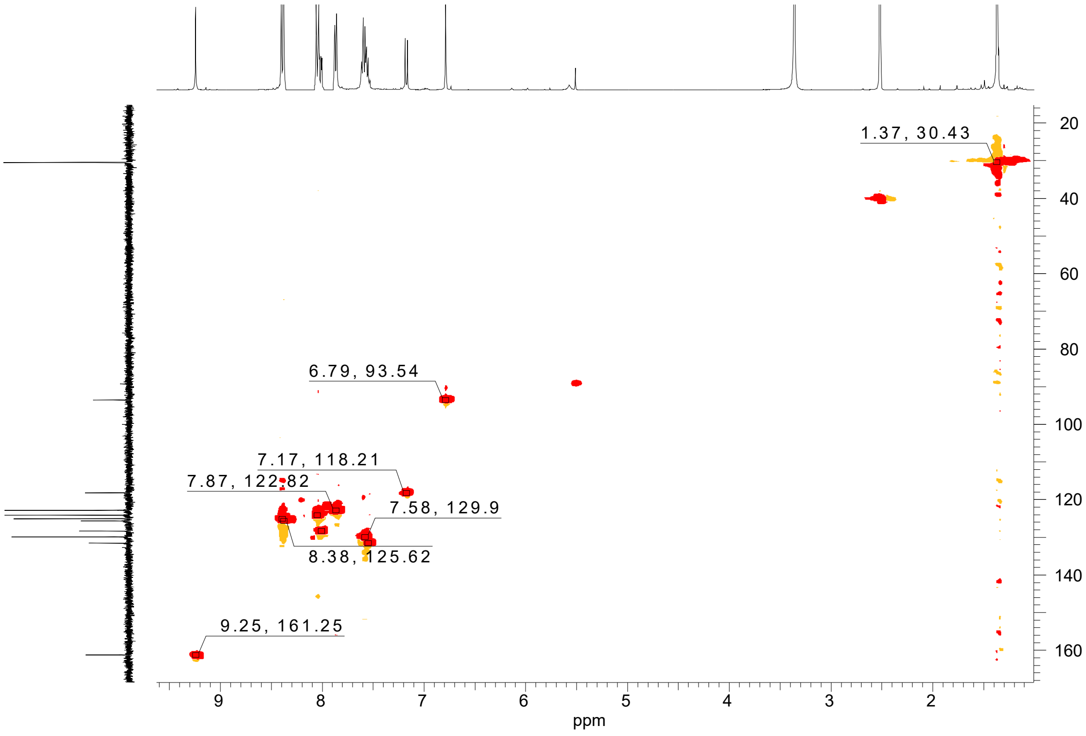

Figure S21. HSQC-NMR spectra of compound **Azo4** in DMSO-*d6*.

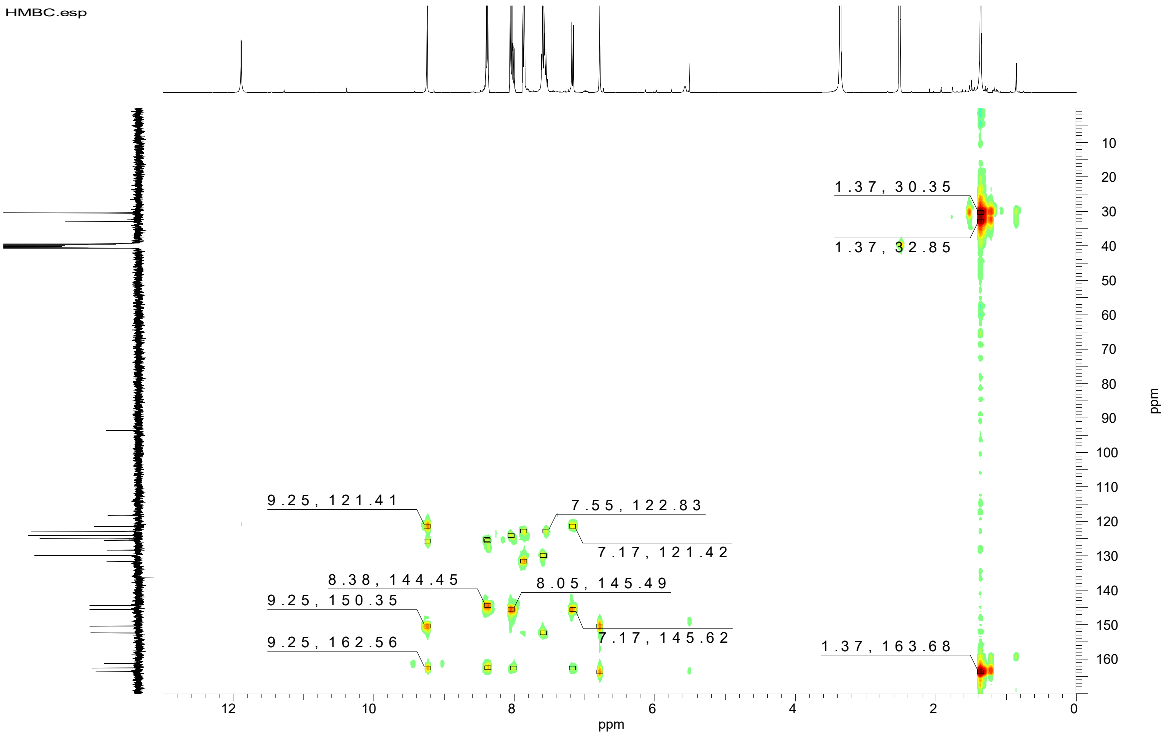

Figure S22. HMBC-NMR spectra of compound **Azo4** in DMSO-*d6*.

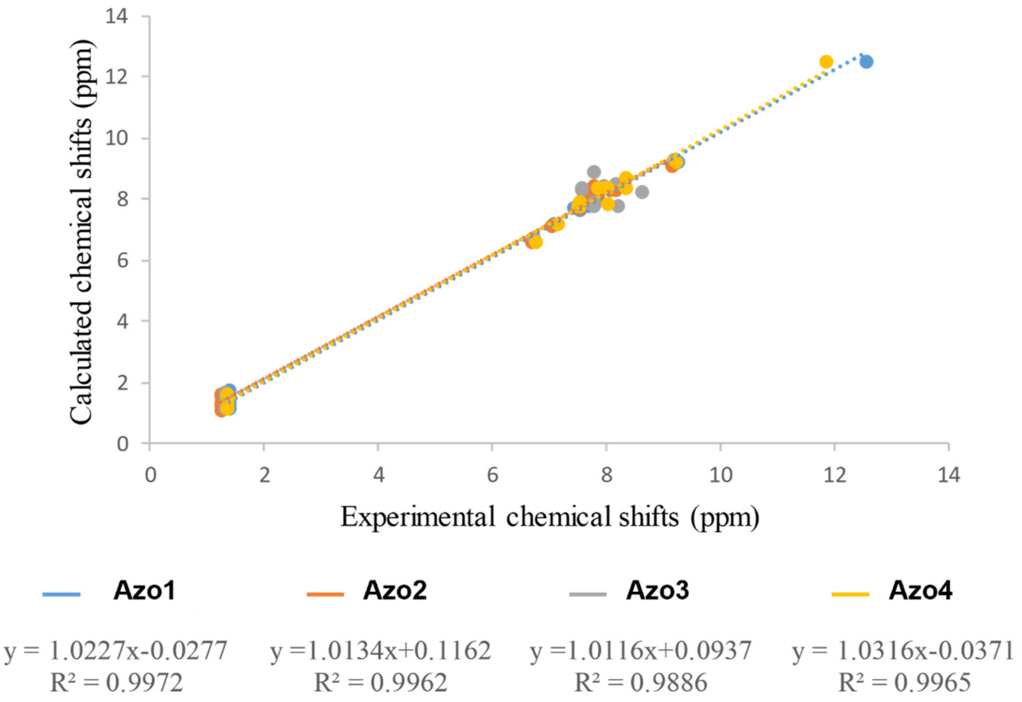

Figure S23. Correlation plots of calculated versus experimental ^{1}H NMR chemical displacements (in ppm) for the compounds **Azo1** to **Azo4**.

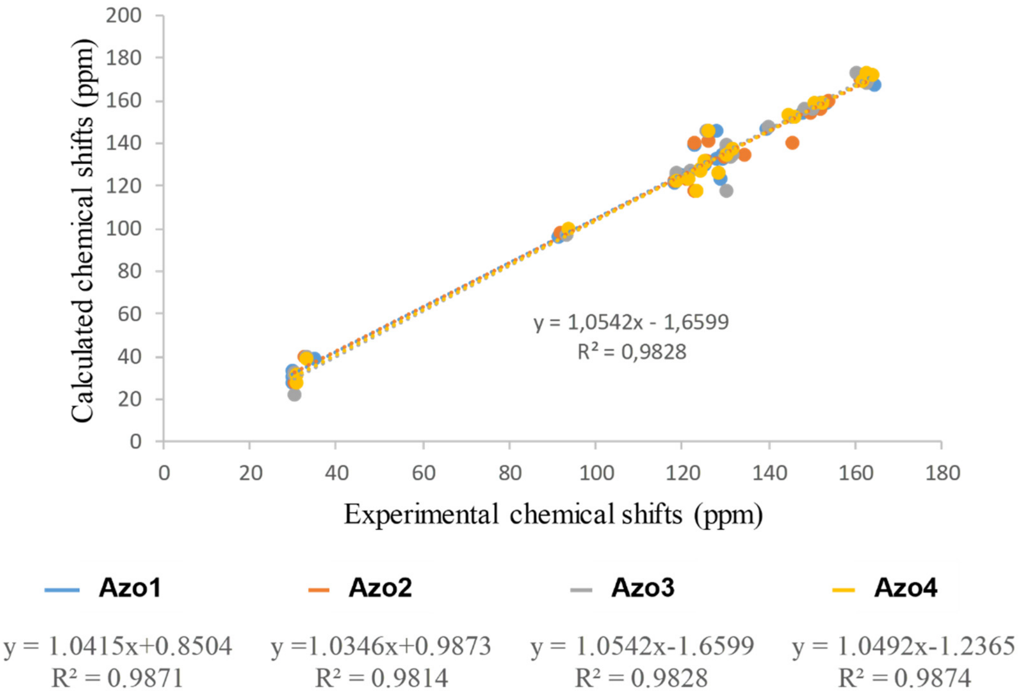

Figure S24. Correlation plots of calculated versus experimental ^{13}C NMR chemical displacements (in ppm) for the compounds **Azo1** to **Azo4**.

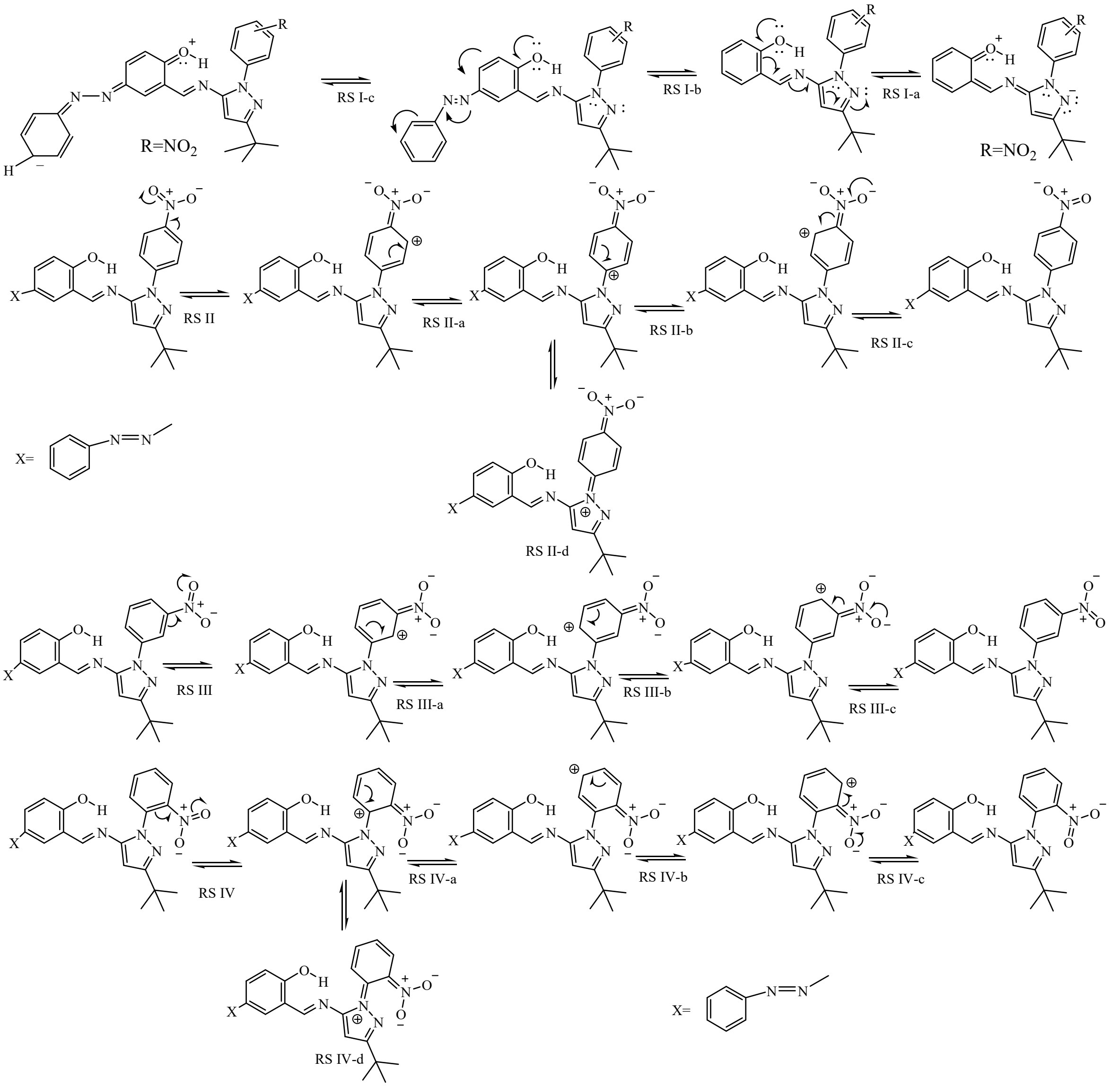

Figure S25. Resonance structures related to the pi delocalization aromatic system in **Azo 1** to **Azo 4** compounds.

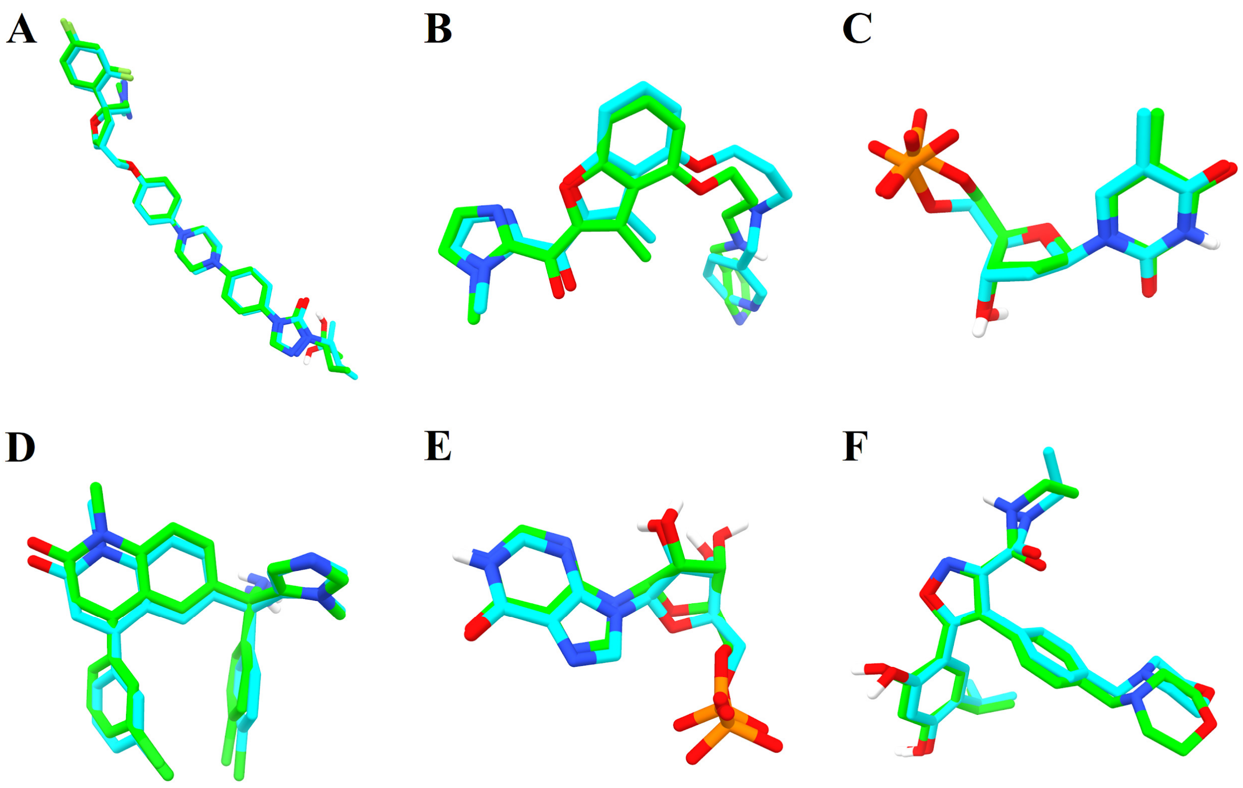

Figure S26. Redocking results. Co-crystallized ligands are shown in green and pose predicted in cyan. (A) Posaconazole inhibitor crystallized on the PDB 5FSA. (B) Inhibitor with imidazole fragment crystallized on the PDB 1IYL. (C) Thymidine-5'-phosphate natural substrate crystallized on the PDB 5UIV. (D) Inhibitor with imidazole fragment crystallized on the PDB 3SFX. (E) Crystallized inhibitor on the PDB 5I34. (F) Crystallized inhibitor on the PDB 7KS9.

Table S1. Experimental and theoretical electronic absorption wavelengths and main orbital contributions for compounds **Azo1** to **Azo4**.

Compound	Exp.	B3LYP/6-311++g(d,p)			
	λ (logε)	λ	E	*f*	Important contributions
**Azo1**	200 (4.366)	195	6.3739	0.0140	H-6→L+2(31%)
					H-12→L+1 (3%)
					H-9→L+2 (5%)
					H-8→L+2 (9%)
					H-7→L+2 (6%)
					H-4→L+5 (2%)
					H-2→L+7 (3%)
					H-2→L+9 (5%)
					H-1→L+7 (4%)
					H-1→L+10 (8%)
					HOMO→L+15 (3%)
					HOMO→L+16 (4%)
	243 (4.461)	243	5.0933	0.0724	HOMO→L+4 (77%)
					H-5→LUMO (3%)
					HOMO→L+5 (5%)
	-	283	4.3834	0.0792	H-6→LUMO (75%)
					H-7→LUMO (3%)
					H-4→LUMO (7%)
					H-4→L+1 (5%)
	335 (4.575)	350	3.5433	0.2735	H-2→LUMO (60%)
					H-1→L+1 (29%)
					H-1→LUMO (7%)
	362 (4.441)	406	3.0505	0.8966	HOMO→LUMO (83%)
					HOMO→L+1 (16%)
**Azo2**	194 (4.542)	-	-	-	-
	234 (4.623)	277	4.4696	0.0444	H-6→L+1 (22%)
					H-5→L+1 (22%)
					HOMO→L+3 (26%)
					H-10→L+2 (2%)
					H-7→L+1 (7%)
					H-7→L+2 (2%)
					H-6→L+2 (8%)
					H-2→L+3 (2%)
	335 (4.798)	373	3.3212	0.4620	HOMO→L+2 (87%)
					H-3→LUMO (4%)
					HOMO→L+1 (4%)
	362 (4.643)	-	-	-	-
**Azo3**	196 (4.382)	199	6.2450	0.0091	H-20→L+1 (40%)
					H-16→L+1 (31%)
					H-16→L+2 (8%)
					H-15→L+1 (2%)
	-	226	5.4867	0.0360	H-10→L+1 (10%)
					H-9→L+1 (13%)
					H-9→L+2 (50%)
					H-3→L+5 (10%)

					H-10→L+2 (4%)
					H-8→L+1 (2%)
					H-2→L+5 (3%)
					HOMO→L+7 (2%)
	234 (4.715)	248	5.0062	0.2758	H-3→L+3 (79%)
					H-7→L+1 (3%)
					H-2→L+3 (6%)
	275 (4.494)	284	4.3508	0.0109	H-7→LUMO (31%)
					H-6→LUMO (61%)
					H-8→LUMO (4%)
	335 (4.843)	372	3.2881	0.5116	HOMO→L+2 (86%)
					H-3→LUMO (2%)
					HOMO→L+1 (7%)
	362 (4.679)	-	-	-	-
**Azo4**	196 (5.273)	196	6.3355	0.0091	H-20→L+1 (40%)
					H-16→L+1 (31%)
					H-16→L+2 (8%)
					H-15→L+1 (2%)
	234 (5.051)	231	5.3722	0.0018	H-1→L+6 (78%)
					H-1→L+3 (7%)
					H-1→L+5 (3%)
					H-1→L+8 (8%)
	275 (4.902)	247	5.0026	0.0034	H-8→L+1 (14%)
					H-2→L+3 (52%)
					H-10→L+1 (3%)
					H-8→L+2 (3%)
					H-6→L+2 (7%)
					H-3→L+3 (4%)
					HOMO→L+3 (7%)
					HOMO→L+4 (3%)
					HOMO→L+5 (3%)
	335 (5.369)	375	3.3013	0.5116	HOMO→L+2 (86%)
					H-3→LUMO (2%)
					HOMO→L+1 (7%)
	362 (5.196)	-	-	-	-

λ: wavelengths (nm); f: oscillator strength; E: excitation energy (eV).

Table S2. Main crystallographic data and refinement parameters for the compounds **Azo1**, **Azo2**, **Azo3** and **Azo4**.

**Compuesto**	**Azo1**	**Azo2**	**Azo3**	**Azo4**
**Molcular formula**	$C_{26}H_{25}N_5O$	$C_{26}H_{24}N_6O_3$	$C_{26}H_{24}N_6O_3$	$C_{26}H_{24}N_6O_3$
**Mass**	423.51	468.51	468.51	468.51
**Crystalline system**	Monoclinic	Triclinic	Monoclinic	Monoclinic
a (Å)	10.4561 (9)	9.5873 (8)	17.0887 (15)	14.217 (2)
b (Å)	21.462 (2)	11.2315 (9)	7.3346 (7)	23.397(4)
c (Å)	10.3282 (9)	12.4374 (10)	19.741 (3)	15.917 (2)
α (°)	90	82.489 (2)	90	90
β (°)	94.416 (3)	73.1688 (2)	102.358 (8)	112.515 (3)
γ (°)	90	75.204 (2)	90	90
**Unit cell volumen**	2303.2 (4)	1237.04 (18)	2417.0 (5)	4891.0 (12)
**Temperature (K)**	298	298	293	298
**Spatial group**	Cc	P_{-1}	$P_{21/n}$	$P_{21/c}$
**Z**	4	2	4	8
**Number of measured reflections**	4246 (2129)	4436	4432	14344
**Final values for R_1 ($I > 2\sigma(I)$)**	0.0524	0.0678	0.0577	0.0903
**Final values for $wR(F^2)$ ($I > 2\sigma(I)$)**	0.1475	0.2322	0.1621	0.2511

molecules MDPI

Table S3. Main BCPs topological properties in **Azo1** molecule.

N°	Bond (x-y)	Rx(Å)	Ry(Å)	ρ_b	$\nabla^2\rho_b$	λ1	λ2	λ3	ε	Type	Group
1	O1-H2	0.699	0.147	4.8548E-01	-3.9223E+00	-3.1708E+00	-3.1272E+00	2.3756E+00	1.3957E-02	covalent	Hydroxyl
2	O1-C38	0.880	0.464	2.9415E-01	-4.5015E-01	-5.8027E-01	-5.6521E-01	6.9533E-01	2.6653E-02	covalent	Phenol
3	H2-N5	0.670	1.228	3.4031E-02	1.2396E-01	-4.7977E-02	-4.6642E-02	2.1858E-01	2.8603E-02	intramolecular	O-H···N=C
4	N3-N4	0.711	0.645	3.3534E-01	-4.8949E-01	-7.2990E-01	-6.5024E-01	8.9065E-01	1.2252E-01	covalent	Pyrazol
5	N3-C11	0.863	0.498	3.1129E-01	-7.8826E-01	-6.5879E-01	-5.2037E-01	3.9091E-01	2.6599E-01	covalent	Pyrazol
6	N3-C25	0.888	0.538	2.7030E-01	-6.6969E-01	-5.1885E-01	-4.9041E-01	3.3957E-01	5.7987E-02	covalent	Pyr-Ph
7	N4-C8	0.838	0.495	3.3931E-01	-9.4781E-01	-7.1955E-01	-6.0519E-01	3.7694E-01	1.8898E-01	covalent	Pyrazol
8	N5-C36	0.821	0.461	3.6966E-01	-9.4375E-01	-8.0518E-01	-7.1570E-01	5.7712E-01	1.2503E-01	covalent	Imide
9	N5-C11	0.823	0.568	3.0296E-01	-8.3689E-01	-6.3916E-01	-5.8688E-01	3.8915E-01	8.9078E-02	covalent	N-Pyr
10	N6-N7	0.620	0.617	4.4412E-01	-9.7967E-01	-1.0772E+00	-9.7273E-01	1.0702E+00	1.0736E-01	covalent	Azo
11	N6-C42	0.840	0.581	2.8850E-01	-7.4261E-01	-5.9188E-01	-5.5255E-01	4.0181E-01	7.1169E-02	covalent	N-Phenol
12	N7-C47	0.846	0.592	2.7876E-01	-6.8860E-01	-5.6334E-01	-5.3593E-01	4.1067E-01	5.1153E-02	covalent	N-Ph
13	C8-C9	0.709	0.695	3.0473E-01	-8.2465E-01	-6.2366E-01	-5.2001E-01	3.1901E-01	1.9931E-01	covalent	Pyrazol
14	C8-C12	0.794	0.715	2.5790E-01	-6.7386E-01	-5.0311E-01	-4.8416E-01	3.1341E-01	3.9147E-02	covalent	Ter-butyl-Pyr
15	C9-H10	0.560	0.371	3.8090E-01	-1.6699E+00	-1.1099E+00	-1.0855E+00	5.2554E-01	2.2439E-02	covalent	Pyrazol
16	C9-C11	0.642	0.729	3.2281E-01	-9.0917E-01	-6.7758E-01	-5.3847E-01	3.0688E-01	2.5833E-01	covalent	Pyrazol
17	C12-C17	0.765	0.745	2.5152E-01	-6.2581E-01	-4.7153E-01	-4.7078E-01	3.1651E-01	1.5996E-03	covalent	Ter-butyl
18	C12-C21	0.765	0.748	2.5126E-01	-6.2423E-01	-4.7142E-01	-4.6984E-01	3.1703E-01	3.3641E-03	covalent	Ter-butyl
19	C12-C13	0.776	0.743	2.4799E-01	-6.0755E-01	-4.6313E-01	-4.6084E-01	3.1641E-01	4.9744E-03	covalent	Ter-butyl
20	C13-H15	0.579	0.380	3.5984E-01	-1.4632E+00	-1.0070E+00	-9.8781E-01	5.3156E-01	1.9417E-02	covalent	Ter-butyl
21	C13-H16	0.580	0.380	3.5881E-01	-1.4554E+00	-1.0025E+00	-9.8253E-01	5.2960E-01	2.0330E-02	covalent	Ter-butyl
22	C13-H14	0.575	0.386	3.5605E-01	-1.4322E+00	-9.8074E-01	-9.5960E-01	5.0819E-01	2.2026E-02	covalent	Ter-butyl
23	C17-H20	0.575	0.384	3.5821E-01	-1.4497E+00	-9.9147E-01	-9.7246E-01	5.1422E-01	1.9544E-02	covalent	Ter-butyl
24	C17-H19	0.578	0.381	3.5907E-01	-1.4573E+00	-1.0005E+00	-9.8134E-01	5.2450E-01	1.9501E-02	covalent	Ter-butyl
25	C17-H18	0.576	0.384	3.5746E-01	-1.4435E+00	-9.8879E-01	-9.6937E-01	5.1466E-01	2.0039E-02	covalent	Ter-butyl
26	C21-H24	0.578	0.382	3.5867E-01	-1.4538E+00	-9.9709E-01	-9.7891E-01	5.2221E-01	1.8570E-02	covalent	Ter-butyl
27	C21-H22	0.575	0.385	3.5714E-01	-1.4401E+00	-9.8500E-01	-9.6663E-01	5.1149E-01	1.9006E-02	covalent	Ter-butyl
28	C21-H23	0.578	0.384	3.5617E-01	-1.4335E+00	-9.8433E-01	-9.6590E-01	5.1671E-01	1.9081E-02	covalent	Ter-butyl
29	C25-C26	0.710	0.658	3.2935E-01	-9.7737E-01	-7.0297E-01	-5.8623E-01	3.1183E-01	1.9913E-01	covalent	Phenyl

30	C25-C34	0.715	0.664	3.2244E-01	-9.3988E-01	-6.8392E-01	-5.7042E-01	3.1445E-01	1.9898E-01	covalent	Phenyl
31	C26-H27	0.562	0.368	3.8967E-01	-1.7520E+00	-1.1485E+00	-1.1443E+00	5.4074E-01	3.6648E-03	covalent	Phenyl
32	C26-C28	0.698	0.686	3.1637E-01	-9.0042E-01	-6.5340E-01	-5.6442E-01	3.1741E-01	1.5764E-01	covalent	Phenyl
33	C28-H29	0.559	0.371	3.8949E-01	-1.7538E+00	-1.1431E+00	-1.1389E+00	5.2820E-01	3.7286E-03	covalent	Phenyl
34	C28-C30	0.692	0.688	3.1959E-01	-9.2167E-01	-6.6289E-01	-5.7614E-01	3.1736E-01	1.5056E-01	covalent	Phenyl
35	C30-H31	0.556	0.373	3.8924E-01	-1.7474E+00	-1.1392E+00	-1.1320E+00	5.2371E-01	6.3557E-03	covalent	Phenyl
36	C30-C32	0.677	0.680	3.3396E-01	-9.9714E-01	-7.0269E-01	-6.0853E-01	3.1408E-01	1.5473E-01	covalent	Phenyl
37	C32-H33	0.558	0.372	3.8899E-01	-1.7476E+00	-1.1400E+00	-1.1340E+00	5.2647E-01	5.3085E-03	covalent	Phenyl
38	C32-C34	0.689	0.700	3.1317E-01	-8.8514E-01	-6.4447E-01	-5.5846E-01	3.1779E-01	1.5402E-01	covalent	Phenyl
39	C34-H35	0.563	0.366	3.9080E-01	-1.7653E+00	-1.1575E+00	-1.1544E+00	5.4664E-01	2.6594E-03	covalent	Phenyl
40	C36-H37	0.566	0.365	3.9589E-01	-1.8184E+00	-1.1910E+00	-1.1897E+00	5.6233E-01	1.0169E-03	covalent	Imide
41	C36-C39	0.735	0.716	2.8484E-01	-7.9167E-01	-5.8686E-01	-5.2473E-01	3.1992E-01	1.1839E-01	covalent	NC-Phenol
42	C38-C45	0.730	0.652	3.2362E-01	-9.5658E-01	-6.9063E-01	-5.7748E-01	3.1153E-01	1.9593E-01	covalent	Phenol
43	C38-C39	0.729	0.684	3.0585E-01	-8.7333E-01	-6.4573E-01	-5.4381E-01	3.1620E-01	1.8741E-01	covalent	Phenol
44	C39-C40	0.705	0.687	3.1202E-01	-8.7722E-01	-6.4296E-01	-5.4799E-01	3.1373E-01	1.7330E-01	covalent	Phenol
45	C40-H41	0.568	0.361	3.9201E-01	-1.7756E+00	-1.1756E+00	-1.1685E+00	5.6845E-01	6.0119E-03	covalent	Phenol
46	C40-C42	0.690	0.700	3.1500E-01	-8.9795E-01	-6.5888E-01	-5.5753E-01	3.1846E-01	1.8178E-01	covalent	Phenol
47	C42-C43	0.704	0.691	3.1593E-01	-9.1889E-01	-6.6870E-01	-5.6813E-01	3.1795E-01	1.7703E-01	covalent	Phenol
48	C43-H44	0.566	0.364	3.9134E-01	-1.7742E+00	-1.1707E+00	-1.1583E+00	5.5488E-01	1.0718E-02	covalent	Phenol
49	C43-C45	0.691	0.683	3.2203E-01	-9.2605E-01	-6.6807E-01	-5.7237E-01	3.1439E-01	1.6719E-01	covalent	Phenol
50	C45-H46	0.563	0.367	3.8806E-01	-1.7357E+00	-1.1428E+00	-1.1357E+00	5.4285E-01	6.3045E-03	covalent	Phenol
51	C47-C48	0.701	0.672	3.2869E-01	-9.8131E-01	-7.0249E-01	-5.9386E-01	3.1505E-01	1.8293E-01	covalent	Phenyl
52	C47-C56	0.712	0.676	3.1738E-01	-9.1670E-01	-6.6333E-01	-5.7002E-01	3.1664E-01	1.6369E-01	covalent	Phenyl
53	C48-H49	0.563	0.368	3.8857E-01	-1.7436E+00	-1.1475E+00	-1.1404E+00	5.4434E-01	6.2507E-03	covalent	Phenyl
54	C48-C50	0.695	0.687	3.1783E-01	-9.1068E-01	-6.5685E-01	-5.7054E-01	3.1670E-01	1.5127E-01	covalent	Phenyl
55	C50-H51	0.556	0.372	3.8993E-01	-1.7556E+00	-1.1418E+00	-1.1368E+00	5.2306E-01	4.4308E-03	covalent	Phenyl
56	C50-C52	0.686	0.686	3.2506E-01	-9.4923E-01	-6.7830E-01	-5.8813E-01	3.1720E-01	1.5332E-01	covalent	Phenyl
57	C52-H53	0.558	0.372	3.8874E-01	-1.7458E+00	-1.1399E+00	-1.1313E+00	5.2547E-01	7.5996E-03	covalent	Phenyl
58	C52-C54	0.687	0.687	3.2329E-01	-9.4425E-01	-6.7316E-01	-5.8786E-01	3.1678E-01	1.4510E-01	covalent	Phenyl
59	C54-H55	0.372	0.557	3.8957E-01	-1.7527E+00	-1.1417E+00	-1.1361E+00	5.2516E-01	4.9348E-03	covalent	Phenyl
60	C54-C56	0.688	0.694	3.1716E-01	-9.0108E-01	-6.5389E-01	-5.6365E-01	3.1646E-01	1.6009E-01	covalent	Phenyl
61	C56-H57	0.565	0.363	3.9265E-01	-1.7813E+00	-1.1729E+00	-1.1690E+00	5.6058E-01	3.3007E-03	covalent	Phenyl

Table S4. Main BCPs topological properties in **Azo2** molecule.

N°	Bond (x-y)	Rx(Å)	Ry(Å)	ρ_b	$\nabla^2\rho_b$	λ1	λ2	λ3	ε	Type	Group
1	N1-N7	0.718	0.647	3.2807E-01	-4.5830E-01	-7.0711E-01	-6.3137E-01	8.8018E-01	1.1996E-01	covalent	Pyrazol
2	N1-C15	0.869	0.505	3.0206E-01	-7.5156E-01	-6.2750E-01	-5.0056E-01	3.7650E-01	2.5358E-01	covalent	Pyrazol
3	N1-C11	0.868	0.544	2.8186E-01	-7.3899E-01	-5.6419E-01	-5.2345E-01	3.4865E-01	7.7836E-02	covalent	Pyr-Ph
4	O2-H3	0.678	0.142	5.2870E-01	-4.4799E+00	-3.6160E+00	-3.5674E+00	2.7035E+00	1.3635E-02	covalent	hydroxyl
5	O2-C17	0.878	0.459	2.9857E-01	-4.1704E-01	-5.9891E-01	-5.7704E-01	7.5890E-01	3.7891E-02	covalent	phenol
6	H3-N4	0.676	1.228	3.3346E-02	1.2746E-01	-4.6743E-02	-4.5194E-02	2.1940E-01	3.4266E-02	intramolecular	O-H···N=C
7	N4-C21	0.821	0.465	3.6677E-01	-9.6232E-01	-7.9401E-01	-7.1056E-01	5.4225E-01	1.1746E-01	covalent	imine
8	N4-C15	0.819	0.568	3.0618E-01	-8.5297E-01	-6.4970E-01	-5.9547E-01	3.9220E-01	9.1069E-02	covalent	N-Pyr
9	N5-N8	0.624	0.621	4.3550E-01	-9.3403E-01	-1.0470E+00	-9.4514E-01	1.0581E+00	1.0773E-01	covalent	Azo
10	N5-C14	0.839	0.577	2.9122E-01	-7.5998E-01	-5.9949E-01	-5.5970E-01	3.9920E-01	7.1089E-02	covalent	N-Phenol
11	O6-N10	0.635	0.579	4.7634E-01	-8.0406E-01	-1.2184E+00	-1.1027E+00	1.5170E+00	1.0490E-01	covalent	NO_2
12	O9-N10	0.638	0.586	4.6409E-01	-7.3214E-01	-1.1747E+00	-1.0638E+00	1.5064E+00	1.0424E-01	covalent	NO_2
13	N10-C12	0.892	0.570	2.5828E-01	-5.8946E-01	-5.0614E-01	-4.6125E-01	3.7793E-01	9.7328E-02	covalent	N-Ph
14	N7-C16	0.839	0.497	3.3701E-01	-9.3710E-01	-7.1199E-01	-5.9865E-01	3.7354E-01	1.8931E-01	covalent	Pyrazol
15	N8-C18	0.846	0.589	2.8006E-01	-6.9826E-01	-5.6599E-01	-5.3941E-01	4.0714E-01	4.9289E-02	covalent	N-Ph
16	C11-C31	0.720	0.655	3.2443E-01	-9.5005E-01	-6.8679E-01	-5.7460E-01	3.1134E-01	1.9525E-01	covalent	Ph
17	C11-C12	0.686	0.702	3.1844E-01	-9.2421E-01	-6.8929E-01	-5.5007E-01	3.1515E-01	2.5310E-01	covalent	Ph
18	C12-C29	0.724	0.653	3.2155E-01	-9.3048E-01	-6.7304E-01	-5.6491E-01	3.0747E-01	1.9139E-01	covalent	Ph
19	C13-C19	0.701	0.686	3.1511E-01	-8.9954E-01	-6.5243E-01	-5.6024E-01	3.1313E-01	1.6454E-01	covalent	Phenol
20	C13-C17	0.677	0.728	3.1124E-01	-8.9732E-01	-6.6029E-01	-5.5261E-01	3.1558E-01	1.9484E-01	covalent	Phenol
21	C13-C21	0.712	0.736	2.8617E-01	-7.9676E-01	-5.9000E-01	-5.2621E-01	3.1945E-01	1.2122E-01	covalent	C-Phenol
22	C14-C19	0.689	0.688	3.2557E-01	-9.5728E-01	-6.9569E-01	-5.7839E-01	3.1680E-01	2.0281E-01	covalent	Phenol
23	C14-C25	0.708	0.690	3.1241E-01	-8.9842E-01	-6.5149E-01	-5.6614E-01	3.1921E-01	1.5075E-01	covalent	Phenol
24	C15-C27	0.732	0.639	3.2290E-01	-9.0801E-01	-6.7710E-01	-5.3603E-01	3.0511E-01	2.6317E-01	covalent	Pyrazol
25	C16-C27	0.708	0.691	3.0767E-01	-8.4364E-01	-6.3192E-01	-5.3062E-01	3.1890E-01	1.9092E-01	covalent	Pyrazol
26	C16-C33	0.788	0.706	2.6521E-01	-7.1051E-01	-5.2134E-01	-5.0264E-01	3.1347E-01	3.7199E-02	covalent	Ter-butyl-Ph
27	C17-C23	0.733	0.655	3.2070E-01	-9.4700E-01	-6.8257E-01	-5.7765E-01	3.1323E-01	1.8163E-01	covalent	Phenol
28	C18-C38	0.700	0.670	3.3087E-01	-9.9336E-01	-7.0873E-01	-5.9997E-01	3.1534E-01	1.8127E-01	covalent	Phenyl
29	C18-C40	0.709	0.671	3.2205E-01	-9.4138E-01	-6.7550E-01	-5.8122E-01	3.1534E-01	1.6220E-01	covalent	Phenyl
30	C19-H20	0.564	0.366	3.9022E-01	-1.7582E+00	-1.1598E+00	-1.1488E+00	5.5049E-01	9.5921E-03	covalent	Phenol
31	C21-H22	0.566	0.364	3.9696E-01	-1.8298E+00	-1.1996E+00	-1.1961E+00	5.6590E-01	2.9776E-03	covalent	imine

32	C23-H24	0.563	0.367	3.8872E-01	-1.7419E+00	-1.1456E+00	-1.1405E+00	5.4419E-01	4.4168E-03	covalent	Phenol
33	C23-C25	0.678	0.683	3.2987E-01	-9.6170E-01	-6.8808E-01	-5.8657E-01	3.1295E-01	1.7306E-01	covalent	Phenol
34	C25-H26	0.570	0.360	3.9343E-01	-1.7925E+00	-1.1904E+00	-1.1786E+00	5.7642E-01	1.0026E-02	covalent	Phenol
35	C27-H28	0.560	0.369	3.8292E-01	-1.6886E+00	-1.1208E+00	-1.0973E+00	5.2953E-01	2.1500E-02	covalent	Pyrazol
36	C29-H30	0.570	0.361	3.9164E-01	-1.7742E+00	-1.1745E+00	-1.1700E+00	5.7022E-01	3.8058E-03	covalent	Phenyl
37	C29-C36	0.697	0.673	3.2503E-01	-9.4843E-01	-6.7616E-01	-5.8721E-01	3.1494E-01	1.5147E-01	covalent	Phenyl
38	C31-C34	0.697	0.689	3.1574E-01	-9.0028E-01	-6.5113E-01	-5.6646E-01	3.1731E-01	1.4948E-01	covalent	Phenyl
39	C33-C56	0.746	0.720	2.7373E-01	-7.3314E-01	-5.2849E-01	-5.2446E-01	3.1981E-01	7.6959E-03	covalent	ter-butyl
40	C33-C48	0.781	0.748	2.4357E-01	-5.8635E-01	-4.5298E-01	-4.4836E-01	3.1499E-01	1.0321E-02	covalent	ter-butyl
41	C33-C52	0.772	0.761	2.4171E-01	-5.7662E-01	-4.4784E-01	-4.4487E-01	3.1609E-01	6.6763E-03	covalent	ter-butyl
42	C34-H35	0.562	0.368	3.9025E-01	-1.7622E+00	-1.1563E+00	-1.1470E+00	5.4116E-01	8.0896E-03	covalent	Phenyl
43	C34-C36	0.686	0.682	3.2791E-01	-9.6694E-01	-6.8646E-01	-5.9733E-01	3.1685E-01	1.4921E-01	covalent	Phenyl
44	C31-H32	0.547	0.384	4.1093E-01	-5.3332E-01	-1.2635E+00	-1.2575E+00	3.8767E-01	4.7795E-03	covalent	Phenyl
45	C36-H37	0.561	0.369	3.9043E-01	-1.7600E+00	-1.1509E+00	-1.1467E+00	5.3762E-01	3.7031E-03	covalent	Phenyl
46	C38-H39	0.563	0.367	3.8937E-01	-1.7521E+00	-1.1519E+00	-1.1443E+00	5.4409E-01	6.6784E-03	covalent	Phenyl
47	C38-C44	0.694	0.688	3.1810E-01	-9.1120E-01	-6.5728E-01	-5.7072E-01	3.1681E-01	1.5168E-01	covalent	Phenyl
48	C40-H41	0.566	0.364	3.9100E-01	-1.7639E+00	-1.1646E+00	-1.1601E+00	5.6086E-01	3.8558E-03	covalent	Phenyl
49	C40-C46	0.683	0.676	3.3134E-01	-9.7715E-01	-6.9253E-01	-5.9897E-01	3.1434E-01	1.5619E-01	covalent	Phenyl
50	C42-H43	0.558	0.373	3.8841E-01	-1.7409E+00	-1.1380E+00	-1.1281E+00	5.2508E-01	8.7732E-03	covalent	Phenyl
51	C42-C46	0.675	0.674	3.4001E-01	-1.0358E+00	-7.1970E-01	-6.2991E-01	3.1384E-01	1.4253E-01	covalent	Phenyl
52	C42-C44	0.683	0.685	3.2718E-01	-9.6087E-01	-6.8348E-01	-5.9387E-01	3.1648E-01	1.5088E-01	covalent	Phenyl
53	C44-H45	0.557	0.373	3.8881E-01	-1.7449E+00	-1.1377E+00	-1.1324E+00	5.2509E-01	4.6841E-03	covalent	Phenyl
54	C46-H47	0.557	0.373	3.8851E-01	-1.7403E+00	-1.1372E+00	-1.1280E+00	5.2492E-01	8.1818E-03	covalent	Phenyl
55	C48-H49	0.580	0.379	3.5940E-01	-1.4608E+00	-1.0066E+00	-9.8451E-01	5.3033E-01	2.2441E-02	covalent	Ter.butyl
56	C48-H50	0.575	0.385	3.5762E-01	-1.4446E+00	-9.8837E-01	-9.6537E-01	5.0910E-01	2.3824E-02	covalent	Ter-butyl
57	C48-H51	0.580	0.380	3.5901E-01	-1.4569E+00	-1.0044E+00	-9.8472E-01	5.3222E-01	2.0018E-02	covalent	Ter-butyl
58	C52-H55	0.578	0.381	3.5921E-01	-1.4585E+00	-1.0011E+00	-9.8165E-01	5.2422E-01	1.9824E-02	covalent	Ter-butyl
59	C52-H53	0.575	0.386	3.5693E-01	-1.4384E+00	-9.8427E-01	-9.6469E-01	5.1053E-01	2.0302E-02	covalent	Ter-butyl
60	C52-H54	0.577	0.384	3.5758E-01	-1.4444E+00	-9.8964E-01	-9.6883E-01	5.1405E-01	2.1475E-02	covalent	Ter-butyl
61	C56-H57	0.577	0.382	3.5871E-01	-1.4522E+00	-9.9575E-01	-9.8024E-01	5.2377E-01	1.5826E-02	covalent	Ter-butyl
62	C56-H59	0.576	0.384	3.5779E-01	-1.4438E+00	-9.8765E-01	-9.7056E-01	5.1439E-01	1.7610E-02	covalent	Ter-butyl
63	C56-H58	0.577	0.384	3.5686E-01	-1.4370E+00	-9.8462E-01	-9.6873E-01	5.1634E-01	1.6409E-02	covalent	Ter-butyl

64	O2-O9	1.740	1.735	2.9580E-03	3.1296E-03	-2.0457E-03	-8.2219E-04	1.5386E-02	1.4881E+00	Intramolecular	Ph-O···O=N
65	N4-N10	1.516	1.425	9.2406E-03	9.4240E-03	-4.7189E-03	-2.1617E-03	4.4577E-02	1.1830E+00	Intramolecular	C=N···N=O
66	N1-O6	1.428	1.409	1.2999E-02	1.3994E-02	-8.5880E-03	-1.0091E-03	6.5572E-02	7.5109E+00	Intramolecular	N=O···N

Table S5. Main BCPs topological properties in **Azo3** molecule.

N°	Bond (x-y)	Rx(Å)	Ry(Å)	ρ_b	$\nabla^2\rho_b$	λ1	λ2	λ3	ε	Type	Group
1	O1-H2	0.709	0.150	4.6871E-01	-3.6910E+00	-2.9767E+00	-2.9351E+00	2.2207E+00	1.4159E-02	covalent	Hydroxyl
2	O1-C41	0.879	0.464	2.9565E-01	-4.5599E-01	-5.8545E-01	-5.6977E-01	6.9923E-01	2.7528E-02	covalent	Phenol
3	H2-N7	0.645	1.209	3.7337E-02	1.2856E-01	-5.5309E-02	-5.3548E-02	2.3741E-01	3.2887E-02	intramolecular	O-H···N=C
4	N7-H24	1.474	1.088	1.2608E-02	4.3507E-02	-9.5897E-03	-7.2359E-03	6.0333E-02	3.2529E-01	intramolecular	C-H···N=C
5	O3-N11	0.637	0.583	4.6900E-01	-7.6609E-01	-1.1906E+00	-1.0826E+00	1.5071E+00	9.9761E-02	covalent	NO_2
6	N7-C18	0.897	0.570	2.5417E-01	-5.6823E-01	-4.9538E-01	-4.4884E-01	3.7599E-01	1.0369E-01	covalent	C- NO_2
7	O4-N11	0.639	0.586	4.6260E-01	-7.3107E-01	-1.1694E+00	-1.0623E+00	1.5006E+00	1.0087E-01	covalent	NO_2
8	N5-N6	0.719	0.647	3.2786E-01	-4.5922E-01	-7.0527E-01	-6.3294E-01	8.7899E-01	1.1428E-01	covalent	Pyrazol
9	N5-C12	0.867	0.503	3.0499E-01	-7.6571E-01	-6.3756E-01	-5.0825E-01	3.8011E-01	2.5442E-01	covalent	Pyrazol
10	N5-C15	0.879	0.542	2.7438E-01	-6.9430E-01	-5.3542E-01	-5.0185E-01	3.4297E-01	6.6889E-02	covalent	Pyr-Ph’
11	N6-C8	0.840	0.486	3.4326E-01	-9.4445E-01	-7.2958E-01	-6.1835E-01	4.0348E-01	1.7987E-01	covalent	Pyrazol
12	N7-C38	0.820	0.459	3.7157E-01	-9.3984E-01	-8.1062E-01	-7.2695E-01	5.9774E-01	1.1510E-01	covalent	imine
13	N7-C12	0.822	0.573	3.0065E-01	-8.2044E-01	-6.3372E-01	-5.8140E-01	3.9468E-01	8.9988E-02	covalent	N-Pyr
14	C8-C9	0.709	0.692	3.0622E-01	-8.3717E-01	-6.2724E-01	-5.2840E-01	3.1847E-01	1.8705E-01	covalent	Pyrazol
15	C8-C25	0.803	0.715	2.5318E-01	-6.5126E-01	-4.9003E-01	-4.7303E-01	3.1180E-01	3.5939E-02	covalent	Ter-butyl-Py
16	C9-H10	0.560	0.370	3.8294E-01	-1.6879E+00	-1.1204E+00	-1.0970E+00	5.2956E-01	2.1312E-02	covalent	Pyrazol
17	C9-C12	0.638	0.728	3.2607E-01	-9.2524E-01	-6.8734E-01	-5.4228E-01	3.0438E-01	2.6749E-01	covalent	Pyrazol
18	N13-N14	0.623	0.620	4.3767E-01	-9.4419E-01	-1.0552E+00	-9.5135E-01	1.0623E+00	1.0914E-01	covalent	Azo
19	N13-C46	0.839	0.589	2.8515E-01	7.1895E-01	-5.8405E-01	-5.4610E-01	4.1120E-01	6.9483E-02	covalent	N-Phenol
20	N14-C49	0.844	0.576	2.8818E-01	-7.5053E-01	-5.8798E-01	-5.5856E-01	3.9602E-01	5.2676E-02	covalent	N-Phenyl
21	C15-C23	0.722	0.661	3.2015E-01	-9.3114E-01	-6.7543E-01	-5.6790E-01	3.1219E-01	1.8933E-01	covalent	Ph’
22	C15-C16	0.714	0.672	3.1814E-01	-9.1885E-01	-6.7128E-01	-5.6337E-01	3.1580E-01	1.9153E-01	covalent	Ph’
23	C16-H17	0.578	0.352	3.9405E-01	1.8023E+00	-1.2027E+00	-1.1995E+00	5.9995E-01	2.5978E-03	covalent	Ph’
24	C16-C18	0.658	0.717	3.2347E-01	-9.3972E-01	-6.8103E-01	-5.6826E-01	3.0957E-01	1.9846E-01	covalent	Ph’
25	C18-C19	0.724	0.653	3.2256E-01	-9.4203E-01	-6.7723E-01	-5.7287E-01	3.0807E-01	1.8216E-01	covalent	Ph’
26	C19-H20	0.569	0.360	3.9228E-01	-1.7805E+00	-1.1762E+00	-1.1739E+00	5.6960E-01	1.9471E-03	covalent	Ph’
27	C19-C21	0.693	0.681	3.2295E-01	-9.3511E-01	-6.7088E-01	-5.8045E-01	3.1621E-01	1.5580E-01	covalent	Ph’

28	C21-H22	0.564	0.367	3.9042E-01	-1.7628E+00	-1.1562E+00	-1.1519E+00	5.4543E-01	3.7378E-03	covalent	Ph'
29	C21-C23	0.684	0.696	3.1899E-01	-9.1638E-01	-6.6044E-01	-5.7206E-01	3.1611E-01	1.5450E-01	covalent	Ph'
30	C23-H24	0.566	0.363	3.9249E-01	-1.7759E+00	-1.1713E+00	-1.1656E+00	5.6091E-01	4.8798E-03	covalent	Ph'
31	C25-C26	0.768	0.746	2.5026E-01	-6.2002E-01	-4.6910E-01	-4.6707E-01	3.1615E-01	4.3329E-03	covalent	Ter-butyl
32	C25-C30	0.770	0.746	2.4855E-01	-6.1202E-01	-4.6480E-01	-4.6289E-01	3.1567E-01	4.1121E-03	covalent	Ter-butyl
33	C25-C34	0.785	0.748	2.4159E-01	-5.7714E-01	-4.4730E-01	-4.4483E-01	3.1499E-01	5.5644E-03	covalent	Ter-butyl
34	C26-H28	0.577	0.383	3.5829E-01	-1.4510E+00	-9.9452E-01	-9.7562E-01	5.1918E-01	1.9374E-02	covalent	Ter-butyl
35	C26-H27	0.578	0.381	3.5873E-01	-1.4548E+00	-9.9882E-01	-9.7952E-01	5.2352E-01	1.9700E-02	covalent	Ter-butyl
36	C26-H29	0.576	0.385	3.5654E-01	-1.4358E+00	-9.8390E-01	-9.6474E-01	5.1288E-01	1.9854E-02	covalent	Ter-butyl
37	C30-H33	0.579	0.381	3.5909E-01	-1.4581E+00	-1.0023E+00	-9.8179E-01	5.2597E-01	2.0899E-02	covalent	Ter-butyl
38	C30-H31	0.576	0.384	3.5772E-01	-1.4461E+00	-9.9057E-01	-9.7039E-01	5.1487E-01	2.0797E-02	covalent	Ter-butyl
39	C30-H32	0.577	0.383	3.5755E-01	-1.4451E+00	-9.9169E-01	-9.7161E-01	5.1816E-01	2.0669E-02	covalent	Ter-butyl
40	C34-H35	0.582	0.378	3.5990E-01	-1.4646E+00	-1.0114E+00	-9.9100E-01	5.3772E-01	2.0539E-02	covalent	Ter-butyl
41	C34-H36	0.575	0.385	3.5736E-01	-1.4438E+00	-9.8823E-01	-9.6515E-01	5.0962E-01	2.3912E-02	covalent	Ter-butyl
42	C34-H37	0.579	0.381	3.5875E-01	-1.4558E+00	-1.0022E+00	-9.8076E-01	5.2713E-01	2.1812E-02	covalent	Ter-butyl
43	C38-H39	0.565	0.364	3.9753E-01	-1.8339E+00	-1.2011E+00	-1.1974E+00	5.6465E-01	3.1455E-03	covalent	imine
44	C38-C40	0.737	0.713	2.8505E-01	-7.9068E-01	-5.8681E-01	-5.2278E-01	3.1891E-01	1.2248E-01	covalent	C-Phenol
45	C40-C47	0.704	0.688	3.1204E-01	-8.8371E-01	-6.4412E-01	-5.5342E-01	3.1383E-01	1.6391E-01	covalent	Phenol
46	C40-C41	0.679	0.728	3.0985E-01	-8.8909E-01	-6.5621E-01	-5.4802E-01	3.1514E-01	1.9741E-01	covalent	Phenol
47	C41-C42	0.734	0.655	3.1923E-01	-9.3807E-01	-6.7788E-01	-5.7305E-01	3.1286E-01	1.8295E-01	covalent	Phenol
48	C42-H43	0.563	0.366	3.8950E-01	-1.7499E+00	-1.1502E+00	-1.1446E+00	5.4486E-01	4.8788E-03	covalent	Phenol
49	C42-C44	0.683	0.687	3.2429E-01	-9.3207E-01	-6.7275E-01	-5.7360E-01	3.1428E-01	1.7286E-01	covalent	Phenol
50	C44-H45	0.571	0.359	3.9397E-01	-1.7987E+00	-1.1939E+00	-1.1828E+00	5.7799E-01	9.3491E-03	covalent	Phenol
51	C44-C46	0.690	0.708	3.1195E-01	-8.9563E-01	-6.4983E-01	-5.6443E-01	3.1863E-01	1.5132E-01	covalent	Phenol
52	C46-C47	0.687	0.684	3.2897E-01	-9.7477E-01	-7.0502E-01	-5.8562E-01	3.1587E-01	2.0388E-01	covalent	Phenol
53	C47-H48	0.563	0.366	3.9092E-01	-1.7642E+00	-1.1622E+00	-1.1515E+00	5.4951E-01	9.3087E-03	covalent	Phenol
54	C49-C58	0.705	0.677	3.2315E-01	-9.5144E-01	-6.8694E-01	-5.8118E-01	3.1668E-01	1.8197E-01	covalent	Phenyl
55	C49-C50	0.713	0.677	3.1601E-01	-9.0980E-01	-6.5967E-01	-5.6667E-01	3.1654E-01	1.6410E-01	covalent	Phenyl
56	C50-H51	0.566	0.364	3.9134E-01	-1.7685E+00	-1.1663E+00	-1.1616E+00	5.5944E-01	4.0470E-03	covalent	Phenyl
57	C50-C52	0.692	0.684	3.2168E-01	-9.2488E-01	-6.6663E-01	-5.7430E-01	3.1604E-01	1.6077E-01	covalent	Phenyl
58	C52-H53	0.559	0.372	3.8817E-01	-1.7409E+00	-1.1368E+00	-1.1317E+00	5.2764E-01	4.4637E-03	covalent	Phenyl
59	C52-C54	0.688	0.690	3.2165E-01	-9.3590E-01	-6.6867E-01	-5.8451E-01	3.1728E-01	1.4399E-01	covalent	Phenyl

60	C54-H55	0.558	0.372	3.8917E-01	-1.7492E+00	-1.1426E+00	-1.1333E+00	5.2673E-01	8.2056E-03	covalent	Phenyl
61	C54-C56	0.686	0.684	3.2658E-01	-9.5775E-01	-6.8222E-01	-5.9199E-01	3.1646E-01	1.5241E-01	covalent	Phenyl
62	C56-H57	0.558	0.372	3.8897E-01	-1.7468E+00	-1.1388E+00	-1.1339E+00	5.2595E-01	4.2904E-03	covalent	Phenyl
63	C56-C58	0.684	0.694	3.2071E-01	-9.2568E-01	-6.6489E-01	-5.7730E-01	3.1650E-01	1.5172E-01	covalent	Phenyl
64	C58-H59	0.562	0.368	3.9015E-01	-1.7583E+00	-1.1547E+00	-1.1466E+00	5.4295E-01	7.0738E-03	covalent	Phenyl

Table S6. Main BCPs topological properties in **Azo4** molecule.

N°	Bond (x-y)	Rx(Å)	Ry(Å)	ρ_b	$\nabla^2\rho_b$	λ1	λ2	λ3	ε	Type	Group
1	O1-H2	0.701	0.149	4.9539E-01	-4.3201E+00	-3.2966E+00	-3.2392E+00	2.2158E+00	1.7716E-02	covalent	OH
2	O1-C9	0.897	0.454	2.9061E-01	-1.7496E-01	-6.1733E-01	-6.1383E-01	1.0562E+00	5.6923E-03	covalent	Ph-OH
3	H2-N5	0.671	1.241	3.2651E-02	1.0257E-01	-4.3659E-02	-4.2439E-02	1.8867E-01	2.8740E-02	intramolecular	O-H···N=C
4	N5-C28	1.464	1.028	1.2832E-02	4.7211E-02	-1.1248E-02	-9.0694E-03	6.7528E-02	2.4020E-01	intramolecular	C=N···H- Ph’
5	O3-N6	0.637	0.581	5.0737E-01	-1.0717E+00	-1.2886E+00	-1.1610E+00	1.3779E+00	1.0994E-01	covalent	NO2
6	O4-N6	0.636	0.579	5.1101E-01	-1.0923E+00	-1.2978E+00	-1.1710E+00	1.3764E+00	1.0830E-01	covalent	NO2
7	N6-C31	0.952	0.510	2.6087E-01	-4.6442E-01	-4.9572E-01	-4.4119E-01	4.7249E-01	1.2359E-01	covalent	Ph-NO_2
8	N5-C18	0.834	0.448	3.7159E-01	-6.1732E-01	-8.6515E-01	-7.4579E-01	9.9361E-01	1.6005E-01	covalent	Imine
9	N5-C25	0.880	0.510	3.0844E-01	-7.7376E-01	-6.5987E-01	-5.9003E-01	4.7614E-01	1.1836E-01	covalent	N-Pyr
10	N7-N8	0.613	0.610	4.9795E-01	-1.3008E+00	-1.2288E+00	-1.0834E+00	1.0114E+00	1.3426E-01	covalent	Azo
11	N7-C13	0.905	0.530	2.8773E-01	-7.1142E-01	-5.7892E-01	-5.3638E-01	4.0388E-01	7.9299E-02	covalent	N-Phenol
12	N8-C49	0.913	0.522	2.8593E-01	-6.7785E-01	-5.7007E-01	-5.3707E-01	4.2928E-01	6.1446E-02	covalent	N-Ph
13	C9-C16	0.840	0.551	3.2793E-01	-1.0030E+00	-6.7141E-01	-5.3482E-01	2.0324E-01	2.5540E-01	covalent	Phenol
14	C9-C10	0.816	0.584	3.2363E-01	-1.0507E+00	-6.5998E-01	-5.2714E-01	1.3646E-01	2.5200E-01	covalent	Phenol
15	C10-C11	0.737	0.654	3.2533E-01	-1.1462E+00	-6.5152E-01	-5.2397E-01	2.9296E-02	2.4343E-01	covalent	Phenol
16	C10-C18	0.627	0.815	3.0177E-01	-1.0051E+00	-6.0357E-01	-5.1401E-01	1.1245E-01	1.7424E-01	covalent	Phenol-C
17	C11-H12	0.492	0.438	3.8711E-01	-1.4616E+00	-1.0442E+00	-1.0277E+00	6.1038E-01	1.6054E-02	covalent	Phenol
18	C11-C13	0.761	0.606	3.4372E-01	-1.2158E+00	-7.2107E-01	-5.5194E-01	5.7184E-02	3.0642E-01	covalent	Phenol
19	C13-C14	0.745	0.642	3.3047E-01	-1.1807E+00	-6.6871E-01	-5.4816E-01	3.6156E-02	2.1991E-01	covalent	Phenol
20	C14-H15	0.497	0.433	3.9078E-01	-1.5301E+00	-1.0679E+00	-1.0488E+00	5.8652E-01	1.8254E-02	covalent	Phenol
21	C14-C16	0.749	0.624	3.3450E-01	-1.1613E+00	-6.7534E-01	-5.1681E-01	3.0850E-02	3.0673E-01	covalent	Phenol

22	C16-H17	0.491	0.439	3.8566E-01	-1.4404E+00	-1.0321E+00	-1.0192E+00	6.1091E-01	1.2692E-02	covalent	Phenol
23	C18-H19	0.494	0.436	3.9563E-01	-1.5774E+00	-1.0990E+00	-1.0836E+00	6.0528E-01	1.4230E-02	covalent	CH
24	N20-N21	0.718	0.651	3.5281E-01	-6.3948E-01	-7.9011E-01	-7.0659E-01	8.5722E-01	1.1821E-01	covalent	Pyr
25	N20-C25	0.899	0.476	3.0522E-01	-4.9929E-01	-6.5711E-01	-5.2361E-01	6.8142E-01	2.5496E-01	covalent	Pyr
26	N20-C26	0.923	0.492	2.8015E-01	-5.1200E-01	-5.4923E-01	-5.2606E-01	5.6330E-01	4.4046E-02	covalent	Pyr-Ph’
27	N21-C22	0.865	0.459	3.4666E-01	-5.9345E-01	-7.6992E-01	-6.5027E-01	8.2673E-01	1.8400E-01	covalent	Pyr
28	C22-C23	0.743	0.653	3.2359E-01	-1.1136E+00	-6.4583E-01	-5.0644E-01	3.8682E-02	2.7525E-01	covalent	Pyr
29	C22-C36	0.844	0.663	2.7051E-01	-8.6260E-01	-5.1369E-01	-4.8994E-01	1.4102E-01	4.8466E-02	covalent	Pyr-terbutyl
30	C23-H24	0.488	0.442	3.7884E-01	-1.3452E+00	-1.0103E+00	-9.6882E-01	6.3387E-01	4.2781E-02	covalent	Pyr
31	C23-C25	0.533	0.832	3.3575E-01	-9.4318E-01	-6.8332E-01	-4.9960E-01	2.3974E-01	3.6773E-01	covalent	Pyr
32	C26-C27	0.823	0.553	3.3436E-01	-1.0504E+00	-6.8268E-01	-5.4991E-01	1.8223E-01	2.4145E-01	covalent	Ph’
33	C26-C34	0.823	0.565	3.2818E-01	-1.0418E+00	-6.6578E-01	-5.3773E-01	1.6175E-01	2.3813E-01	covalent	Ph’
34	C27-H28	0.495	0.435	3.8948E-01	-1.4913E+00	-1.0483E+00	-1.0379E+00	5.9498E-01	1.0034E-02	covalent	Ph’
35	C27-C29	0.615	0.762	3.3233E-01	-1.1450E+00	-6.7082E-01	-5.2341E-01	4.9226E-02	2.8163E-01	covalent	Ph’
36	C29-H30	0.553	0.377	4.128E-01	-5.4039E-01	-1.2833E+00	-1.2818E+00	4.0351E-01	1.1841E-03	covalent	Ph’
37	C29-C31	0.565	0.808	3.3658E-01	-1.0974E+00	-6.8455E-01	-5.6104E-01	1.4819E-01	2.2013E-01	covalent	Ph’
38	C31-C32	0.811	0.554	3.4061E-01	-1.0993E+00	-6.9544E-01	-5.7346E-01	1.6956E-01	2.1271E-01	covalent	Ph’
39	C32-C33	0.499	0.432	3.9082E-01	-1.5378E+00	-1.0620E+00	-1.0519E+00	5.7613E-01	9.6104E-03	covalent	Ph’
40	C32-C34	0.773	0.605	3.3182E-01	-1.1265E+00	-6.6979E-01	-5.2110E-01	6.4430E-02	2.8534E-01	covalent	Ph’
41	C34-H35	0.497	0.433	3.9047E-01	-1.5233E+00	-1.0538E+00	-1.0469E+00	5.7735E-01	6.6224E-03	covalent	Ph’
42	C36-C45	0.784	0.657	3.0589E-01	-1.1000E+00	-5.8364E-01	-5.8133E-01	6.4991E-02	3.9747E-03	covalent	terbutyl
43	C36-C37	0.746	0.766	2.6944E-01	-8.9649E-01	-5.0551E-01	-5.0086E-01	1.0987E-01	9.2801E-03	covalent	terbutyl
44	C36-C41	0.763	0.803	2.4497E-01	-7.4225E-01	-4.4959E-01	-4.4707E-01	1.5440E-01	5.6416E-03	covalent	terbutyl
45	C37-H40	0.505	0.454	3.6008E-01	-1.2448E+00	-8.8899E-01	-8.8299E-01	5.2720E-01	6.7963E-03	covalent	terbutyl
46	C37-H39	0.507	0.453	3.5941E-01	-1.2509E+00	-8.8709E-01	-8.8222E-01	5.1841E-01	5.5189E-03	covalent	terbutyl
47	C37-H38	0.508	0.452	3.5966E-01	-1.2553E+00	-8.9025E-01	-8.8574E-01	5.2065E-01	5.0969E-03	covalent	terbutyl
48	C41-H43	0.507	0.452	3.6041E-01	-1.2620E+00	-8.9462E-01	-8.8659E-01	5.1921E-01	9.0537E-03	covalent	terbutyl

49	C41-H42	0.507	0.453	3.5922E-01	-1.2498E+00	-8.8701E-01	-8.7915E-01	5.1640E-01	8.9466E-03	covalent	terbutyl
50	C41-H44	0.507	0.455	3.5853E-01	-1.2399E+00	-8.8504E-01	-8.7594E-01	5.2110E-01	1.0389E-02	covalent	terbutyl
51	C45-H47	0.506	0.454	3.5858E-01	-1.2258E+00	-8.8224E-01	-8.7853E-01	5.3493E-01	4.2288E-03	covalent	terbutyl
52	C45-H48	0.509	0.451	3.5959E-01	-1.2501E+00	-8.8895E-01	-8.8364E-01	5.2252E-01	6.0092E-03	covalent	terbutyl
53	C45-H46	0.513	0.448	3.6163E-01	-1.2891E+00	-8.9720E-01	-8.9279E-01	5.0087E-01	4.9308E-03	covalent	terbutyl
54	C49-C50	0.775	0.590	3.4528E-01	-1.2031E+00	-7.1415E-01	-5.6756E-01	7.8610E-02	2.5828E-01	covalent	Ph
55	C49-C58	0.792	0.599	3.2667E-01	-1.0964E+00	-6.5482E-01	-5.3505E-01	9.3503E-02	2.2386E-01	covalent	Ph
56	C50-H51	0.491	0.440	3.8514E-01	-1.4472E+00	-1.0337E+00	-1.0218E+00	6.0831E-01	1.1651E-02	covalent	Ph
57	C50-C52	0.744	0.683	3.0629E-01	-1.0408E+00	-6.0594E-01	-4.7895E-01	4.4080E-02	2.6514E-01	covalent	Ph
58	C52-H53	0.486	0.444	3.8403E-01	-1.4017E+00	-1.0205E+00	-1.0202E+00	6.3905E-01	3.3426E-04	covalent	Ph
59	C52-C54	0.758	0.617	3.3588E-01	-1.1722E+00	-6.7862E-01	-5.3406E-01	4.0502E-02	2.7070E-01	covalent	Ph
60	C54-C56	0.582	0.753	3.6218E-01	-1.3059E+00	-7.5157E-01	-6.0445E-01	5.0164E-02	2.4340E-01	covalent	Ph
61	C56-H57	0.486	0.444	3.8358E-01	-1.3825E+00	-1.0243E+00	-1.0120E+00	6.5380E-01	1.2084E-02	covalent	Ph
62	C56-C58	0.585	0.774	3.4413E-01	-1.1741E+00	-7.0089E-01	-5.5160E-01	7.8360E-02	2.7064E-01	covalent	Ph
63	C58-H59	0.494	0.437	3.8832E-01	-1.4761E+00	-1.0455E+00	-1.0371E+00	6.0647E-01	8.1722E-03	covalent	Ph
64	C54-H55	0.486	0.444	3.8339E-01	-1.3890E+00	-1.0278E+00	-1.0101E+00	6.4887E-01	1.7485E-02	covalent	Ph

Table S7. Molecular descriptors (I, A, η, σ, χ, μ, and ω) for **Azo1** to **Azo4** compounds calculated using the B3LYP/6-311++g(d,p) approximation level. Values are expressed in eV.

**Descriptor**	**Formula**	**Azo1**	**Azo2**	**Azo3**	**Azo4**
Ionization potential	$I = -E_{HOMO}$	6.227	6.292	6.275	6.303
Electron affinity	$A = -E_{LUMO}$	2.712	3.069	3.122	3.111
Hardness	$\eta = (I - A)/2$	1.757	1.612	1.576	1.596
Softness	$\sigma = 1/\eta$	0.285	0.310	0.317	0.313
Chemical potential	$\mu = -\chi = -(I + A)/2$	4.469	4.680	4.698	4.707
Electrophilicity index	$\omega = \mu^2/2\eta = \chi^2/2\eta$	-4.469	-4.680	-4.698	-4.707

As noted in Table S7, the compound **Azo1** has the lowest ionization potential, electron affinity, softness, and electrophilicity values, but the highest hardness value in this series, indicating that this compound is a poorer electrophile and electron acceptor agent compared to its nitro congeners (**Azo2**-**Azo4**). Within the group of nitro derivatives, the **Azo3** compound (*m*-NO_2 substituent) has the most negative electrophilicity and the highest softness value, which makes it the compound with the lowest kinetic stability and the highest reactivity and electrophilic character of the entire series.
